# Patient discharge from intensive care: an updated scoping review to identify tools and practices to inform high-quality care

**DOI:** 10.1186/s13054-021-03857-2

**Published:** 2021-12-17

**Authors:** Kara M. Plotnikoff, Karla D. Krewulak, Laura Hernández, Krista Spence, Nadine Foster, Shelly Longmore, Sharon E. Straus, Daniel J. Niven, Jeanna Parsons Leigh, Henry T. Stelfox, Kirsten M. Fiest

**Affiliations:** 1 Department of Critical Care Medicine, Cumming School of Medicine, University of Calgary and Alberta Health Services, 3134 Hospital Drive NW, Calgary, AB T2N 4Z6 Canada; 2 Knowledge Translation Program, Li Ka Shing Knowledge Institute, St. Michael’s Hospital, 209 Victoria Street, East Building, Toronto, ON M5B 1W8 Canada; 3 Department of Geriatric Medicine, Faculty of Medicine, University of Toronto, 6 Queen’s Park Crescent West, Third Floor, Toronto, ON M5S 3H2 Canada; 4 Department of Community Health Sciences and O’Brien Institute for Public Health, Cumming School of Medicine, University of Calgary, 3134 Hospital Drive NW, Calgary, AB T2N 4Z6 Canada; 5 Faculty of Health, School of Health Administration, Dalhousie University, Sir Charles Tupper Medical Building, 2nd Floor, 5850 College Street, Halifax, NS B3H 4R2 Canada; 6 Hotchkiss Brain Institute, Cumming School of Medicine, University of Calgary, 3134 Hospital Drive NW, Calgary, AB T2N 4Z6 Canada; 7 Department of Psychiatry, Cumming School of Medicine, University of Calgary and Alberta Health Services, 3134 Hospital Drive NW, Calgary, AB T2N 4Z6 Canada

**Keywords:** Critical care, Intensive care, Transitions in care, Quality of care, Patient discharge

## Abstract

**Background:**

Critically ill patients require complex care and experience unique needs during and after their stay in the intensive care unit (ICU). Discharging or transferring a patient from the ICU to a hospital ward or back to community care (under the care of a general practitioner) includes several elements that may shape patient outcomes and overall experiences. The aim of this study was to answer the question: what elements facilitate a successful, high-quality discharge from the ICU?

**Methods:**

This scoping review is an update to a review published in 2015. We searched MEDLINE, EMBASE, CINAHL, and Cochrane databases from 2013-December 3, 2020 including adult, pediatric, and neonatal populations without language restrictions. Data were abstracted using different phases of care framework models, themes, facilitators, and barriers to the ICU discharge process.

**Results:**

We included 314 articles from 11,461 unique citations. Two-hundred and fifty-eight (82.2%) articles were primary research articles, mostly cohort (118/314, 37.6%) or qualitative (51/314, 16.2%) studies. Common discharge themes across all articles included adverse events, readmission, and mortality after discharge (116/314, 36.9%) and patient and family needs and experiences during discharge (112/314, 35.7%). Common discharge facilitators were discharge education for patients and families (82, 26.1%), successful provider-provider communication (77/314, 24.5%), and organizational tools to facilitate discharge (50/314, 15.9%). Barriers to a successful discharge included patient demographic and clinical characteristics (89/314, 22.3%), healthcare provider workload (21/314, 6.7%), and the impact of current discharge practices on flow and performance (49/314, 15.6%). We identified 47 discharge tools that could be used or adapted to facilitate an ICU discharge.

**Conclusions:**

Several factors contribute to a successful ICU discharge, with facilitators and barriers present at the patient and family, health care provider, and organizational level. Successful provider-patient and provider-provider communication, and educating and engaging patients and families about the discharge process were important factors in a successful ICU discharge.

**Supplementary Information:**

The online version contains supplementary material available at 10.1186/s13054-021-03857-2.

## Background

Transitions in care occur when a patient is being moved between healthcare settings (e.g., intensive care unit [ICU] to hospital ward) or providers (e.g., changes in nursing shift) [1]. Transitions in care are complex, requiring communication and coordination of care between multiple healthcare providers [1]. Incomplete or inaccurate transfer of information between healthcare providers during transitions in care may result in unnecessary healthcare utilization (e.g., unnecessary medications[2] or low-value care[3]), adverse events[4, 5], medical errors[2, 4] and poor patient and family satisfaction of care [6–10].

Transitions in care of critically ill patients from the ICU are even more complex because they include a change of care setting and often include a change in health status[11] characterized by severe illness [12], exacerbation of chronic medical problems [13], and newly acquired physical[12] (e.g., weakness) and psychiatric[12] (e.g., delirium) injuries. While some institutions have ICU discharge guidelines, their consistent application in practice varies [14–17]. Differences between ICU and ward care may also make transitions in care challenging; this includes patients transitioning from a unit with specialized technology and monitoring and lower nurse to patient ratios (ICU) to a less acute environment with higher nurse to patient ratios (ward) [18–20].

Admission to an ICU and subsequent transitions in care impact many patients and caregivers each year [21–24]. By improving transitions in care, patients and families may feel more satisfied with care [25], and may have fewer adverse outcomes including re-hospitalizations [4, 5]. The quality of transitions in care is one metric used by the World Health Organization (WHO) and Joint Commission International (JCI) to evaluate hospital performance [26]. As such, the transitions in care literature has rapidly evolved over the past five years. A scoping review from our team published in 2015 reviewed the transitions in care literature and identified 224 articles that described discharge themes and patient, provider, and institutional factors that act as facilitators and/or barriers to patient care during transitions in care [27]. A recent scoping review of 37 articles described the transitions of adult ICU populations to inpatient wards [18]. The authors reported practices that had positive (e.g., adequate communication between ICU and ward staff) and negative (e.g., afterhours or weekend discharges) impacts on the transition in care from the ICU to a hospital ward [18]. Our review includes these elements, and adds to the literature by summarizing current evidence and practices around transitions in care of critically ill neonatal, pediatric, and adult populations. Our review also includes transitions in care from the ICU to a hospital ward, and transitions in care directly back to the community, a practice becoming increasingly common at some institutions [28–30]. We also provide an overview of tools used in these settings to facilitate successful transitions in care.

## Methods

We followed scoping review frameworks by Arksey and O’Malley[31] and Levac and colleagues[32] to update the previous scoping review by Stelfox and colleagues in 2015 [27]. We followed the Preferred Reporting Items for Systematic reviews and Meta-Analyses extension for Scoping Reviews (PRISMA-ScR; see Additional File 1) [33].

### Search strategy

We searched MEDLINE, EMBASE, CINAHL, and Cochrane Reviews Databases on December 3, 2020, basing our search strategy on the 2015 scoping review [27]. The search was restricted from 2013-present with no language restrictions. The MEDLINE search strategy is available in Additional file 2. Results were downloaded and imported into reference management software EndNote Version X9 (Clarivate Analytics, Philadelphia, PA, USA, 2013) and were managed using Microsoft Excel (Microsoft Corporation, 2016).

### Article selection

Articles were included if they were peer-reviewed, described adult, pediatric, or neonatal populations, and primarily focused on the structure, process, or outcome of discharge (e.g., the decision to discharge a patient on home mechanical ventilation or not, the use of a guideline or checklist during discharge, or evaluating patient outcomes based on differences in the time of day discharge occurred, respectively) as defined by the Donabedian model for evaluating quality of healthcare [34, 35]. Articles were excluded if they were included in the 2015 scoping review [27].

Populations in included studies could be patients, family members or other caregivers of patients, or healthcare providers (e.g., physicians, nurses, allied healthcare providers). We included both primary (e.g., cohort, qualitative, and cross-sectional studies) and secondary research articles (e.g., reviews, editorials, and consensus methodologies). Articles were excluded if they described transfers between ICUs, transfers to ICUs (e.g., transfers from a coronary care unit, intermediate unit, or step-down unit), did not primarily discuss the structure, process, or outcome of an ICU discharge, or if we were unable to find the full-text article. Two research assistants piloted the inclusion and exclusion criteria with 100 titles and abstracts to ensure the criteria were applied consistently. Six research assistants reviewed each title and abstract independently and in duplicate. If either reviewer indicated that the reference should move on to full-text review, it was included.

After piloting inclusion and exclusion criteria on 10% of full-text articles to ensure consistency across research assistants, all full-text articles were reviewed independently and in duplicate. If consensus could not be reached between the two research assistants, a third was consulted. If articles were not available in English, they were translated using Google Translate, [36] which has been shown to be a reliable tool for translating documents for systematic reviews [37].

### Data abstraction and analysis

**Fig. 1:**
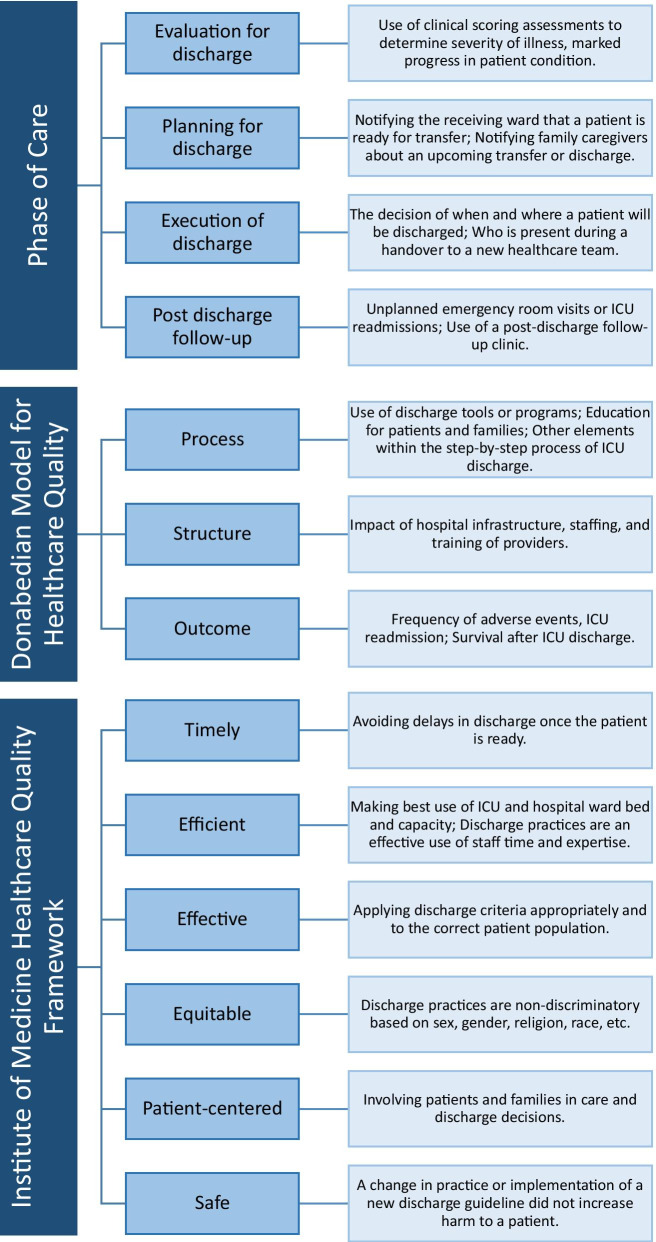
Framework elements and examples used for article classification

After piloting a standardized form in Excel, research assistants abstracted data from each article, which included study information (e.g., location, study dates, study design) and ICU characteristics (e.g., population, speciality). Articles were classified as primary research (e.g., cohort, qualitative, cross-sectional, randomized controlled trials [RCT], other non-RCT interventional studies, case study or series, scoping or systematic reviews including meta-analyses) or secondary and descriptive research (e.g., narrative or literature reviews, consensus methodologies using existing literature, opinion pieces). Each article was classified according to the phase of care examined, Donabedian framework stage (process, structure, or outcome of discharge) [34, 35], and the Institute of Medicine (IoM) Health Care Quality Framework (safe, effective, efficient, timely, patient-centered, equitable) [38]. Articles were assessed based on ICU discharge themes including adverse events, readmission, and mortality following discharge, patient and family needs and experiences during discharge, amongst others. Discharge facilitators and barriers were assessed at a patient and family level, a provider level, and at an organizational level. For themes, facilitators, and barriers, reviewers categorized each article from a pre-established framework, and could indicate if additional elements were present in the article. Figure 1 and Table 1 provide a summary and example for each framework and theme. Each article was screened for tools that could facilitate a successful ICU discharge. Tools could be guidelines or checklists, transfer tools, educational tools, discharge assessments, discharge letters, transfer brochures, prediction tools, triage models, or peer support programs. Descriptions of each type of tool is available in Additional File 3.

**Table 1:** Summary of data abstraction for themes, and facilitators and barriers to a successful transition in care

Theme	Example
*ICU Discharge Themes*	
Adverse events, readmission, and mortality following discharge	ICU or emergency department readmission rates; Mortality following ICU discharge in a given time frame (e.g., one year after discharge)
Patient and family needs and experiences during discharge	Patients or families desire for more information about the next steps in care; Appreciation for the attentiveness of nursing staff in ICU
Planning for discharge	Notifying subsequent care providers about the patient’s condition; Aligns with planning for discharge in the phase of care model
Continuity of patient care	Use of a transition program or follow-up clinics—patients understand where to seek care after ICU discharge
Discharge education for patients and families	Programs that provide information on what is to be expected after discharge and when to seek medical help
Standardizing the discharge process	Use of guidelines or protocols to ensure the discharge process is the same for all patients
Availability of complete and accurate discharge information	Use of medical records, checklists, or summaries to provide appropriate information to either healthcare providers, family members, or patients
Evaluating patient readiness for discharge	Use of clinical scoring assessments to determine severity of illness, marked progress in patient condition; Aligns with phase of care examined
Anxiety associated with discharge	Patient or family feelings of anxiousness about transitioning to a different level of care or worrying about leaving the ICU
Timeliness of discharge	Time of day discharge occurs (daytime versus nighttime), and if there is a delay in discharge (patient has been ready for discharge for several days but has not been transitioned out of ICU)
Resource use during discharge	Use of supplies, infrastructure, or staff time to facilitate the discharge
Critical care transition program	Presence of a dedicated team that works with ICU and the receiving care providers to improve the transition. May include a nurse liaison or outreach team
Medication reconciliation	Verifying that medications started in the ICU should be continued after discharge
Autonomy	Patients feeling like they have a say in their discharge and/or subsequent care
Discharge education for providers	Programs that teach ward staff what to expect from an ICU patient; Education for ICU providers about facilitating a successful ICU discharge
*Facilitators for a successful ICU discharge*	
Patients and family	Discharge education for patients and families; Family engagement/support system; Provider-patient communication; Patient demographic and clinical characteristics; Written communication for patients and families; Expectations of patients/family; Patient/family are treated as members of the healthcare team; Patient/family feelings of self-efficacy; Use of coping mechanisms; Excited, joyous to be leaving the ICU
Healthcare providers	Provider-provider communication; Critical care transition programs (e.g., outreach, liaison nurse); Collaboration between ICU and ward; Written documentation for providers; Knowledge/experience of provider; Clinical judgment or decision-making; Clear roles/responsibility for providers; Multidisciplinary team; Provider leadership; Provider empathy to patient and family
Organization	Tools to facilitate discharge; Impact of current discharge practices on flow and performance; Guidelines or policies; Use of best practices; Discharge location from ICU; Education/training of providers; Time of discharge (day of week or time of day); Availability of follow-up clinics or home support programs; Admission location before ICU; Hospital characteristics (e.g., trauma level);
*Barriers to a successful ICU discharge*	
Patients and family	Patient demographic and clinical characteristics; Feelings of patient and family anxiety, embarrassment; Expectations of patients/family; Physical and psychological effects of illness (e.g., pain, nightmares; Lack of provider-patient communication; ICU and hospital length of stay; Financial obstacles (lack of insurance, cost of care); Socioeconomic factors of patient/ family; Logistical barriers to providing support (e.g., family lives far from hospital); Lack of familial support; Feelings of lack of control
Healthcare providers	Provider workload; Lack of provider-provider communication; Lack of knowledge/experience of provider; Provider anxiety
Organization	Impact of current discharge practices on flow and performance; Delay in discharge; Time of discharge (day of week or time of day); Limited ICU and ward resources; Costs of healthcare provided; Hospital characteristics (e.g., trauma level); Hospital or ICU capacity; Admission location before ICU; Physical and technological infrastructure (small patient rooms, no electronic health records; Lack of education/training of providers; Reduction in the levels of technology and monitoring when transition from ICU to ward; Restricted visitation policies

Abstracted data was verified by another research assistant. Discrepancies between reviewers were discussed, and a third reviewer was consulted if needed. Data analyses were completed using STATA version 14.2 for Windows (StataCorp LP, College Station, Texas, USA, 2015). Aligning with scoping review methodology [31, 32], articles were not assessed for quality or risk of bias.

## Results

**Fig. 2:**
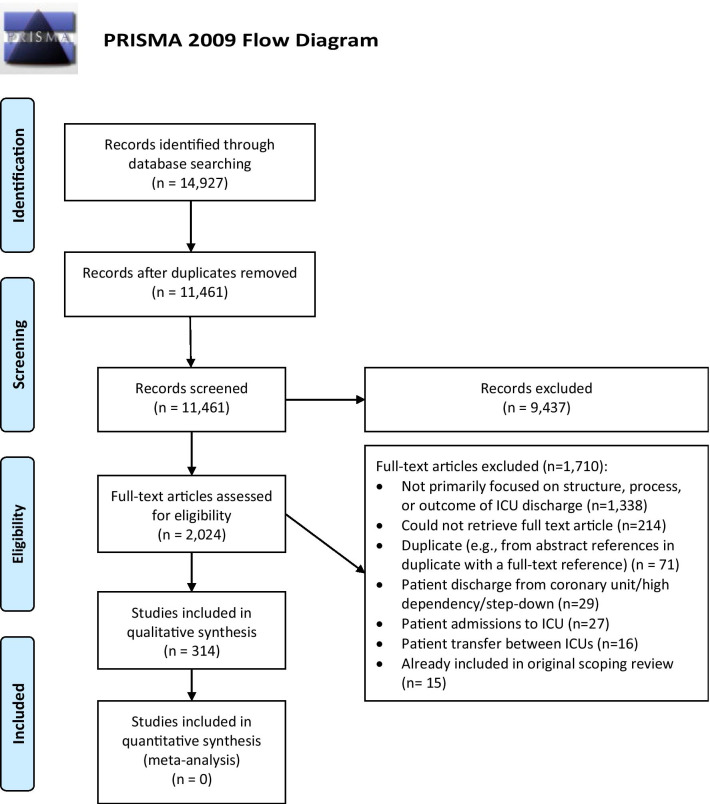
PRISMA flow diagram

The search identified 11,461 unique articles (Fig. 2). Of these, we reviewed 2,024 full-text articles; 314 articles were included in the review. The most common reason articles were excluded was because they did not focus primarily on the structure, process, or outcome of ICU discharge (n = 1,338, 78.2%) or we could not retrieve the full-text, English version of the article (includes one article that could not be translated from Persian to English[39], and 16 protocol registrations of which a subsequent full-text publication could not be found. The remaining were 184 citations where only conference abstracts were available, and 13 articles we were unable to obtain from foreign journals; n = 214, 12.5%; Fig. 2).

### Description of the articles

There were 269 (85.7%) primary research articles and 45 (14.3%) secondary or descriptive articles (Table 2). Most primary research articles were retrospective or prospective cohort studies (118/269, 43.9%) or qualitative studies (51/269, 19.0%). Most secondary research articles were literature or narrative reviews (35/45, 77.8%). Articles were mainly from North American and European countries (135/314, 43.0% and 80/314, 25.5%, respectively), and published in English (304/314, 96.8%). Articles focused on adult ICU (162/314, 51.6%), neonatal (97/314, 30.9%), or pediatric patients (34/314, 10.8%), with some studies including more than one population. Thirty-one studies (9.9%) did not state a specific age for the included population, nor could this be inferred from the included patient demographic information. Most adult ICUs were medical (79/314, 25.2%) or surgical (73/162, 23.3%). Article characteristics are described in Table 2.

**Table 2:** Characteristics of included articles

Characteristic	All studies, n (%) (N = 314)
*Type of study*	
Primary research	
Cohort studies	118 (37.6)
Qualitative study	51 (16.2)
Interventional (non-RCT)	39 (12.4)
Cross-sectional	28 (8.9)
Systematic and scoping reviews^a^	11 (3.5)
Randomized controlled trial	8 (2.6)
Mixed methods	8 (2.6)
Case study or series	6 (1.9)
Secondary research	
Literature and narrative reviews	23 (7.3)
Descriptive studies	7 (2.2)
Guidelines	6 (1.9)
Delphi methodology and consensus statements	5 (1.6)
Opinion	4 (1.3)
Continent of Origin	
North America	135 (43.0)
Europe	80 (25.5)
Asia	35 (11.2)
Oceania	23 (7.3)
Various (multiple) continents	16 (5.1)
South America	10 (3.2)
Africa	1 (0.3)
Not reported	14 (4.5)
Language	
Published in English	304 (96.8)
Non-English language publication	10 (3.2)
Year of publication	
2012–2015^b^	98 (31.2)
2016–2018	110 (35.0)
2019–2021	106 (33.8)
Study population^c^	
Adult	162 (51.6)
Pediatric	34 (10.8)
Neonatal	97 (30.9)
Not reported	31 (9.9)
Type of ICU^a^	
Medical	79 (25.2)
Surgical	73 (23.3)
General system	30 (9.6)
Cardiac	23 (7.3)
Neurological	21 (6.7)
Trauma	19 (6.1)
Oncologic	8 (2.6)
Burn	4 (1.3)
Mixed (with sub-types not specified)	2 (0.6)
Not reported	80 (25.5)

*ICU* intensive care unit, *RCT* randomized controlled trial

^a^Includes integrative reviews

^b^While the search was restricted to 2013, late indexing brought forward articles with an official publishing date of 2013

^c^Reponses are not mutually exclusive and add up to more than 100%

### Patient care frameworks

Phases of care examined, Donabedian framework elements, and IoM framework elements for each article are summarized in Table 3. Phases of care include execution of discharge (185/314, 58.9%), post-discharge follow-up (159/314, 50.6%), planning for discharge (93/314, 29.6%), and evaluation for discharge (i.e., patient readiness; 42/314, 13.4%). Most articles reported on the Donabedian framework element of process of discharge (199/314, 63.4%) followed by the outcome (159/314, 50.6%), and structure of discharge (94/314, 29.9%).The most common IoM framework element in included articles was safety (161/314, 51.3%), followed by patient-centeredness (153/314, 48.7%). Additional dimensions were effective (112/314, 35.7%), efficient (85/314, 27.1%) timely (54/314, 17.2%), or equitable (5/314, 1.6%) elements of care.

**Table 3:** Distribution of articles according to phase of care during discharge from ICU and quality of care frameworks

Characteristic, n(%)	All studies (n = 314)	Adult (n = 162)	Pediatric (n = 34)	Neonatal (n = 97)	Not reported (n = 31)
*Phase of care*					
Execution of discharge	185 (58.9)	95 (58.6)	25 (73.5)	50 (51.5)	24 (77.4)
Post-discharge follow-up	159 (50.6)	95 (58.6)	13 (38.2)	45 (46.4)	11 (35.5)
Planning for discharge	93 (29.6)	28 (17.3)	14 (41.2)	50 (51.5)	7 (22.6)
Evaluation for discharge	42 (13.4)	17 (10.5)	5 (14.7)	17 (17.5)	3 (9.7)
*Donabedian Framework*					
Process	199 (63.4)	86 (53.1)	21 (61.8)	77 (79.4)	24 (77.4)
Outcome	159 (50.6)	105 (64.8)	11 (32.4)	36 (37.1)	10 (32.3)
Structure	94 (29.9)	44 (27.2)	18 (52.9)	25 (25.8)	14 (45.2)
*Institute of medicine framework*					
Safe	161 (51.3)	93 (57.4)	16 (47.1)	40 (41.2)	16 (51.6)
Patient-centered	153 (48.7)	56 (34.6)	18 (52.9)	71 (73.2)	13 (41.9)
Effective	112 (35.7)	54 (33.3)	16 (47.1)	36 (37.1)	13 (41.9)
Efficient	85 (27.1)	48 (29.6)	13 (38.2)	18 (18.6)	13 (41.9)
Timely	54 (17.2)	37 (22.8)	7 (20.6)	6 (6.2)	9 (29.0)
Equitable	5 (1.6)	1 (0.6)	0 (0.0)	3 (3.1)	1 (3.2)

Responses are not mutually exclusive across and within categories and add up to more than 100%

There were differences in each of the frameworks’ elements when comparing adult, pediatric, and neonatal populations. For example, studies in adult populations more frequently reported on the outcome of discharge (105/162, 64.8%), when compared to pediatric (11/34, 32.4%) and neonatal populations (36/97, 37.1%). Neonatal populations reported on the execution of, and planning for, discharge (50/97, 51.5% for both), whereas only 17.3% of adult populations were classified as planning for discharge (28/162). The least commonly reported IoM Framework element across all groups was equity (adult: 1/162, 0.6%; pediatric: 0/34, 0%; neonatal: 3/97, 3.1%) (Table 3). A complete overview of phase of care and framework elements is available in Additional File 4.

### ICU discharge themes

The most common ICU discharge theme was adverse events (116/314, 36.9%), and patient and family needs and experiences during discharge (112/314, 35.7%). These were followed by planning for discharge (95/314, 30.3%), continuity of patient care (84/314, 26.8%), and discharge education for patients and families (72/314, 22.9%). The least common themes were medication reconciliation (24/314, 7.6%), autonomy (20/314, 6.4%), and discharge education for providers (17/314, 5.4%). Discharge themes are summarized in Additional File 5.

### Facilitators and barriers to discharge

Some elements of a successful transition in care were identified as being both a facilitator and a barrier. For example, patient demographic and clinical characteristics could be a facilitator due to an absence of co-morbidities (49/314, 15.6%), but also a barrier such as increased severity of illness and therefore, decreased likelihood of a “successful” ICU discharge (89/314, 28.3%; e.g., as measured by Acute Physiology and Chronic Health Evaluation II (APACHE II) scores). For healthcare providers, common facilitators for a successful ICU discharge were provider-provider communication (77/314, 24.5%; e.g., sufficient communication between ICU healthcare providers and those on the receiving ward), and critical care transition programs (55/314, 17.5%; e.g., use of a nurse liaison or transition team that works with providers and patients for a smooth transition experience). Common barriers were provider workload (i.e., overburdened; 21/314, 6.7%) and a lack of provider-provider communication (20/314, 6.4%). Organizational facilitators included tools to facilitate discharge (50/314, 15.9%; e.g., guidelines) and the impact of current discharge practices on flow and performance (36/314, 11.5%; e.g., a standardized workflow in place when handing a patient over from ICU to ward). Conversely, the impact of current discharge practices was also commonly identified as a barrier at the organizational level (49/314, 15.6%; e.g., lack of standardized processes to facilitate discharge), and delays in discharge (32/314, 10.2%; e.g., when a patient is ready to leave the ICU, but the discharge is delayed). All facilitators and barriers are summarized in Table 4, and the facilitators and barriers for each study are reported in Additional File 4.

**Table 4:** Facilitators and barriers to care during discharge from the ICU

Factor	Facilitator/ Barrier	All studies, n(%) (N = 314)
*Patient/family*		
Facilitators		
Discharge education for patients and families	Facilitator	82 (26.1)
Family engagement/support system	Facilitator	80 (25.5)
Provider-patient communication	Facilitator	77 (24.5)
Patient demographic and clinical characteristics	Facilitator	49 (15.6)
Written communication for patients and families	Facilitator	26 (8.3)
Expectations of patients/family	Facilitator	25 (8.0)
Patient/family are treated as members of the healthcare team	Facilitator	4 (1.3)
Patient/family feelings of self-efficacy	Facilitator	4 (1.3)
Use of coping mechanisms	Facilitator	3 (1.0)
Excited, joyous to be leaving the ICU	Facilitator	3 (1.0)
Barriers		
Patient demographic and clinical characteristics	Barrier	89 (28.3)
Feelings of patient and family anxiety, embarrassment	Barrier	70 (22.3)
Expectations of patients/family	Barrier	25 (8.0)
Physical and psychological effects of illness (e.g., pain, nightmares)	Barrier	23 (7.3)
Lack of provider-patient communication	Barrier	11 (3.5)
ICU and hospital length of stay	Barrier	9 (2.9)
Financial obstacles (e.g., lack of insurance, cost of care)	Barrier	5 (1.6)
Socioeconomic factors of patient/family	Barrier	5 (1.6)
Logistical barriers to providing support (e.g., family lives far from hospital)	Barrier	5 (1.6)
Lack of familial support	Barrier	2 (0.6)
Feelings of lack of control	Barrier	2 (0.6)
*Provider*		
Facilitators		
Provider-provider communication	Facilitator	77 (24.5)
Critical care transition programs (e.g., outreach, liaison nurse)	Facilitator	55 (17.5)
Collaboration between ICU and ward	Facilitator	44 (14.0)
Written documentation for providers	Facilitator	42 (13.4)
Knowledge/experience of provider	Facilitator	31 (9.9)
Clinical judgment or decision-making	Facilitator	30 (9.6)
Clear roles/responsibility for providers	Facilitator	17 (5.4)
Multidisciplinary team	Facilitator	8 (2.6)
Provider leadership	Facilitator	1 (0.3)
Provider empathy to patient and family	Facilitator	1 (0.3)
*Barriers*		
Provider workload	Barrier	21 (6.7)
Lack of provider-provider communication	Barrier	20 (6.4)
Lack of knowledge/experience of provider	Barrier	11 (3.5)
Provider anxiety	Barrier	6 (1.9)
*Organizational*		
Facilitators		
Tools to facilitate discharge	Facilitator	50 (15.9)
Impact of current discharge practices on flow and performance	Facilitator	36 (11.5)
Guidelines or policies	Facilitator	31 (9.9)
Use of best practices	Facilitator	30 (9.6)
Discharge location from ICU	Facilitator	30 (9.6)
Education/training of providers	Facilitator	29 (9.2)
Time of discharge (day of week or time of day)	Facilitator	15 (4.8)
Availability of follow-up clinics or home support programs	Facilitator	15 (4.8)
Admission location before ICU	Facilitator	7 (2.2)
Hospital characteristics (e.g., trauma level)	Facilitator	5 (1.6)
*Barriers*		
Impact of current discharge practices on flow and performance	Barrier	49 (15.6)
Delay in discharge	Barrier	32 (10.2)
Time of discharge (day of week or time of day)	Barrier	27 (8.6)
Discharge location from ICU	Barrier	25 (8.0)
Limited ICU and ward resources	Barrier	24 (7.6)
Costs of healthcare provided	Barrier	20 (6.4)
Hospital characteristics (e.g., trauma level)	Barrier	13 (4.1)
Hospital or ICU capacity	Barrier	10 (3.2)
Admission location before ICU	Barrier	5 (1.6)
Physical and technological infrastructure (e.g., small patient rooms, no electronic health records)	Barrier	5 (1.6)
Lack of education/training of providers	Barrier	4 (1.3)
Staffing (e.g., change in nurse-to-patient ratios, not enough staff)	Barrier	4 (1.3)
Reduction in the levels of technology and monitoring when transition from ICU to ward	Barrier	2 (0.6)
Restricted visitation policies	Barrier	1 (0.3)

Responses are not mutually exclusive within or across categories and add up to more than 100%

### Tools to facilitate ICU discharge

Forty-seven studies included tools to facilitate a successful ICU discharge (15.0%). Of discharge tools described, most were guidelines or checklists (21/47, 44.7%; procedures to standardize discharge planning and ensuring all steps are completed). Other tools identified include transfer tools (7/47, 14.9%; procedures to facilitate an effective ICU to ward transfer), educational tools (4/7, 8.5%; for patients and/ or family prior to discharge to prepare them), discharge assessments (4/47, 8.5%; evaluating readiness for discharge and may include calculating risk for adverse events), discharge letters (3/47, 6.4%; summarized information about the patient’s ICU stay), transfer brochures (2/47, 4.3%; information for patients and families about the transfer process), prediction tools (2/47, 4.3%; to identify patients who may have adverse outcomes after discharge), triage models (1/47, 2.1%; to identify patients with the greatest need of continued ICU care), and peer support programs (1/47, 2.1%; facilitating space for family members and patients to connect about a shared experience). Of these 47 tools, 18 (38.3%) were in adult populations, 16 (34.0%) in neonatal populations, and six (12.7%) in pediatric populations. A complete breakdown of available tools and study populations is available in Additional File 3.

## Discussion

In this scoping review, we evaluated 314 articles that described a successful discharge from the ICU according to the phase of care examined, and the relevant Donabedian [34, 35] and IoM[38] framework elements. We identified facilitators and barriers to a successful ICU discharge at the patient and family, healthcare provider, and organizational levels. These include discharge education for patients and family members, communication between patients and healthcare providers and between healthcare providers themselves, and the use of tools to facilitate a successful discharge. Forty-seven articles described a discharge tool, where the majority of tools were guidelines or checklists, which institutions could adapt according to their institutional practices and unique patient populations.

Communication between patients and providers was an important facilitator for a successful ICU discharge as indicated by approximately one-quarter of included studies. Some studies had similar findings about provider-patient communication, where patients and families valued summaries about the patient’s stay in the ICU [40] and information about next steps [25, 41]. Patients and families also appreciated being an active member of the healthcare team when deciding if the patient was ready to transfer out of the ICU [25, 40]. Communication between healthcare providers was also a facilitator to a successful ICU discharge; this included communication between the ICU care team and the hospital ward team, or the ICU care team and a patient’s primary care provider. Communication between members of the healthcare team could be verbal (e.g., face-to-face, telephone) [42, 43], or written (e.g., a summary of the patients ICU stay in the medical chart) [42–44]. One study described the experience of nursing staff on a hospital ward, and how these nurses desired complete and coordinated information about an ICU patient [42]. The nurses on the ward described the benefits of having pre-planned transfers and open lines of communication so they could ask relevant follow-up questions to best care for the patient [42]. Other articles described the challenges that patients and primary care providers may encounter when a patient is discharged into the community [44, 45]. Primary care providers expressed interest in being provided information about the patient’s ICU stay, and acknowledged that they do not have the same level of knowledge about the associated conditions that an intensivist would have [44, 45]. Former patients echoed that it would be beneficial for both them and their primary care providers to have the information so they could address next steps for care together [44].

Approximately one-third of articles studied patient and family needs and experiences during the ICU discharge process, including their desire for consistent communication from the healthcare team, the experience of transitioning to a hospital ward with different staffing ratios, and the most common facilitator for a successful ICU discharge being education for patients and families. Ingram et al. [46] found that education about the discharge process reduced post-discharge emergency room visits and overall costs associated with care. Others have found that addressing the needs of patients, based in Maslow’s hierarchy of needs framework (e.g., physical, emotional) [47] can influence a successful ICU discharge process [48]. By anticipating a patient’s concerns and involving their family in support and care for the patient, ICUs can promote a patient- and family- centered approach [49]. Burns and colleagues provided suggestions to incorporate successful patient- and family- centered care and improve engagement in the ICU; these included offering opportunities for patients and families to provide feedback (ranging from short, anonymous surveys to being members of a committee) and encouraging flexibility from care providers and researchers when responding to patients and families in these settings [50]. These reviews and our data demonstrate that patient and family needs should be considered when designing effective discharge criteria and guidelines.

## Strengths and limitations

Our scoping review has several strengths. Our search strategy was developed with an academic librarian who has experience in systematic and scoping reviews and included multiple databases. We used established review methodology where appropriate, and our search was not restricted by language. By translating articles, we were able to capture more tools and discussions about the discharge process from several global areas, versus English-speaking countries alone. Another strength of our study is the inclusion of secondary research, including reviews and editorials. We screened conference proceedings to identify additional relevant full-text articles that may not have been indexed to appear in the database search. Despite our comprehensive search strategy there is still the chance that we missed articles. There were cases where full-text articles were unavailable, and the translation of some non-English articles may have left out pertinent information. We did not directly email authors of articles where we were unable to retrieve a full-text copy of a publication, whether this was a conference abstract or a publication in a non-English language journal. We reached out to some authors via ResearchGate, and utilized two institutional access databases and inter-facility loans to retrieve as many articles as possible without purchasing additional accesses. The categorization of the articles while conducting the review is subjective; not all studies clearly define which framework (phases of care, Donabedian, and IoM) elements were the focus of their study. Despite team members’ training and review of these frameworks prior to data abstraction, and verification by a second reviewer, it is possible articles may have been misclassified. Finally, as this is a scoping review, the results reported are high-level information about a successful ICU discharge and may benefit from a systematic review to further describe the impacts of certain practices on ICU discharges.

## Conclusions

Using IoM [38] and Donabedian [34, 35] frameworks for high-quality care, we identified several themes, facilitators, and barriers to successful ICU discharges across adult, pediatric, and neonatal populations. Commonly reported facilitators to a successful ICU discharge included the education and engagement of patients and family members in the process, and communication between healthcare providers. Future reviews could provide more insight on the impacts of patient and family needs and experiences. Tools to facilitate discharge could utilize elements from these tools and adapt them to their own circumstances to provide discharge tools that facilitate successful transitions in care from the ICU to the hospital ward or home.

## Supplementary Information

** Additional file 1**. Preferred Reporting Items for Systematic reviews and Meta-Analyses extension for Scoping Reviews (PRISMA-ScR) Checklist

** Additional file 2**. MEDLINE Search Strategy

** Additional file 3**. Tools to facilitate a successful ICU discharge

** Additional file 4**. Article classification according to study population, describing framework elements, themes, and facilitators and barriers

** Additional file 5**. Discharge themes of articles included in the review

## Data Availability

Data sharing is not applicable to this article as no datasets were generated or analysed during the current study.

## Abbreviations

APACHE-II: Acute Physiology and Chronic Health Evaluation II
IoM: Institute of medicine
ICU: Intensive care unit
JCI: Joint Commission International
NICU: Neonatal Intensive care unit
PICU: Pediatric intensive care unit
PRISMA-ScR: Preferred reporting items for systematic reviews and meta-analyses: scoping review
RCT: Randomized control trial
WHO: World Health Organization
