# Supplementary material for: Patient discharge from intensive care: an updated scoping review to identify tools and practices to inform high-quality care
Source: Crit Care. 2021 Dec 17;25:438. doi: 10.1186/s13054-021-03857-2 (PMC8684123; doi:10.1186/s13054-021-03857-2)
Supplement: Supplementary file 2 — Additional file 2. MEDLINE Search Strategy [file 13054_2021_3857_MOESM2_ESM.pdf]

Additional File 2 - MEDLINE Search Strategy

Vendor: OVID
Resource: Ovid MEDLINE(R) and Epub Ahead of Print, In-Process & Other Non-Indexed Citations and Daily 1946 to December 03, 2020
Search strategy ran on: December 3, 2020

1. (critical adj care).tw.
2. (critical$ adj ill$).tw.
3. (intensive adj care).mp.
4. ICU?.tw.
5. (cardiovascular adj unit?).tw.
6. (coronary adj care).tw.
7. CCU?.tw.
8. (step-down adj unit?).tw.
9. (burn adj unit?).tw.
10. "high dependency unit?".tw.
11. (neurosurgical adj unit?).tw.
12. (observation adj unit?).tw.
13. exp Intensive Care Units/
14. exp Critical Care/
15. Critical Illness/
16. or/1-15
17. (discharg$ adj1 plan$).tw.
18. (discharg$ adj1 process$).tw.
19. (discharg$ adj1 protocol?).tw.
20. (discharg$ adj1 method$).tw.
21. (discharg$ adj1 transition$).tw.
22. "discharg$ of patient?".tw.
23. (patient$ adj1 transition$).tw.
24. (patient$ adj1 discharg$).tw.
25. (patient$ adj1 transfer$).tw.
26. (transfer$ adj1 process$).tw.
27. (transfer$ adj1 plan$).tw.
28. (transfer$ adj3 ward$).tw.
29. "transfer$ of patient?".tw.
30. Patient Discharge/
31. Patient Transfer/
32. or/17-31
33. 16 and 32
34. Animals/ not (Animals/ and Humans/)

35. 33 not 34
36. limit 35 to yr="2013 -Current"
37. (critical adj care).tw.
38. (critical$ adj ill$).tw.
39. (intensive adj care).mp.
40. ICU?.tw.
1. 41 (cardiovascular adj unit?).tw.
41. (coronary adj care).tw.
42. CCU?.tw.
43. (step-down adj unit?).tw.
44. (burn adj unit?).tw.
45. "high dependency unit?".tw.
46. (neurosurgical adj unit?).tw.
47. (observation adj unit?).tw.
48. exp Intensive Care Units/
49. exp Critical Care/
50. Critical Illness/
51. or/37-51
52. (discharg$ adj1 plan$).tw.
53. (discharg$ adj1 process$).tw.
54. (discharg$ adj1 protocol?).tw.
55. (discharg$ adj1 method$).tw.
56. (discharg$ adj1 transition$).tw.
57. "discharg$ of patient?".tw.
58. (patient$ adj1 transition$).tw.
59. (patient$ adj1 discharg$).tw.
60. (patient$ adj1 transfer$).tw.
61. (transfer$ adj1 process$).tw.
62. (transfer$ adj1 plan$).tw.
63. (transfer$ adj3 ward$).tw.
64. "transfer$ of patient?".tw.
65. Patient Discharge/
66. Patient Transfer/
67. or/53-67
68. 52 and 68
69. Animals/ not (Animals/ and Humans/)
70. 69 not 70
71. limit 71 to yr="2013 -Current"
