# Supplementary material for: Patient discharge from intensive care: an updated scoping review to identify tools and practices to inform high-quality care
Source: Crit Care. 2021 Dec 17;25:438. doi: 10.1186/s13054-021-03857-2 (PMC8684123; doi:10.1186/s13054-021-03857-2)
Supplement: Supplementary file 3 — Additional file 3. Tools to facilitate a successful ICU discharge [file 13054_2021_3857_MOESM3_ESM.pdf]

Additional File 3 - Tools to facilitate a successful ICU discharge

Tool	Purpose	All studies (n=47)	Adult (n=18)	Pediatric (n=6)	Neonatal (n=16)	Not reported (n=7)
Guideline or checklist	To standardize discharge planning and ensure all necessary steps are completed [1-21]	21 (44.7)	5 (27.8)	3 (50.0)	8 (50.0)	5 (71.4)
Transfer tool	A procedure used to facilitate an effective ICU transfer to a hospital ward [22-28]	7 (14.9)	4 (22.2)	1 (16.7)	1 (6.3)	1 (14.3)
Educational tool	Education for patients and/ or family prior to discharge [29-33]	4 (8.5)	1 (5.6)	1 (16.7)	3 (18.8)	0 (0)
Discharge assessment	Evaluate readiness for discharge; risk assessment [34-38]	4 (8.5)	2 (11.1)	0 (0)	3 (18.5)	0 (0)
Discharge letter	Provide summarized information about the patient's stay in the ICU [39-41]	3 (6.4)	2 (11.1)	0 (0)	0 (0)	1 (14.3)
Transfer brochure	Provide information for family/patient about the transfer process [42, 43]	2 (4.3)	1 (5.6)	1 (16.7)	0 (0)	0 (0)
Prediction tool	Identify patients who may have adverse outcomes after discharge [44, 45]	2 (4.3)	2 (11.1)	0 (0)	0 (0)	0 (0)
Triage model	Identify patients with greatest need of continued ICU care [46]	1 (2.1)	1 (5.6)	0 (0)	0 (0)	0 (0)
Peer support program	Facilitate a space for family members and patients to connect about a shared experience [47]	1 (2.1)	0 (0)	0 (0)	1 (6.3)	0 (0)

Note: Data are listed as n(%)

References

1. Coon EA, Kramer NM, Fabris RR, Burkholder DB, Klaas JP, Graff-Radford J, Arthur Moore S, Wijdicksv EFM, Britton JW, Jones LK: **Structured handoff checklists improve clinical measures in patients discharged from the neurointensive care unit**. *Neurol: Clin Pract* 2015, **5**(1):42-49.
2. D'Angelo RG, Rincavage M, Tata AL, Millstein LS, Gulati MS, Flurie RW, Gonzales JP: **Impact of an antipsychotic discontinuation bundle during transitions of care in critically ill patients**. *J Intensive Care Med* 2019, **34**(1):40-47.
3. Murray NM, Joshi AN, Kronfeld K, Hobbs K, Bernier E, Hirsch KG, Gold CA: **A standardized checklist improves the transfer of stroke patients from the neurocritical care unit to hospital ward**. *Neurohospitalist* 2020, **10**(2):100-108.
4. Powell M, Brown D, Davis C, Walsham J, Calleja P, Nielsen S, Mitchell M: **Handover practices of nurses transferring trauma patients from intensive care units to the ward: A multimethod observational study**. *Aust Crit Care* 2020, **33**(6):538-545.

5. Yun SH, Oh EG, Yoo YS, Kim SS, Jang YS: **Development and effects of a transition nursing program for patients and family caregivers at a neurological ICU in Korea**. *Clin Nurs Res* 2017, **26**(1):27-46.
6. de la Oliva P, Cambra-Lasaosa FJ, Quintana-Diaz M, Rey-Galan C, Sanchez-Diaz JI, Martin-Delgado MC, de Carlos-Vicente JC, Hernandez-Rastrollo R, Holanda-Pena MS, Pilar-Orive FJ *et al*: **Admission, discharge and triage guidelines for paediatric intensive care units in Spain**. *Med Intensiva* 2018, **42**(4):235-246.
7. Kulesa JT, Balsara SL, Ghebremariam ET, Colyer J: **Designing a process for cardiology patient transfers: A quality improvement, descriptive study on interprovider communication and resident education**. *Pediatr Qual Saf* 2020, **5**(3):e300.
8. Neupane B, McFeeters M, Johnson E, Hickey H, Pandya H: **Transitioning children requiring long-term ventilation from hospital to home: A practical guide**. *Paediatr Child Health* 2015, **25**(4):187-191.
9. Bapat R, McClead R, Shepherd E, Ryshen G, Bartman T: **Challenges, successes and opportunities for reducing readmissions in a referral-based children's hospital NICU**. *J Neonatal-Perinatal Med* 2016, **9**(4):433-440.
10. Bathie J, Shaw J: **Early discharge home from the neonatal unit with the support of naso-gastric tube feeding**. *J Neonatal Nurs* 2013, **19**(4):213-216.
11. Bowles JD, Jnah AJ, Newberry DM, Hubbard CA, Roberston T: **Infants with technology dependence: Facilitating the road to home**. *Adv Neonatal Care* 2016, **16**(6):424-429.
12. Brodsgaard A, Zimmermann R, Petersen M: **A preterm lifeline: Early discharge programme based on family-centred care**. *J Spec Pediatr Nurs* 2015, **20**(4):232-243.
13. Fleming PJ, Ingram J, Johnson D, Blair PS: **Estimating discharge dates using routinely collected data: improving the preparedness of parents of preterm infants for discharge home**. *Arch Dis Child Fetal Neonatal Ed* 2017, **102**(2):F170-F172.
14. Giudici L, Rodriguez D, Alonso MM, Bertani G, Cattaino A, Fernandez P, Aguilera NM, Muniagurria G, Marzan G, Pose G: **Guidelines for discharge planning of the high risk newborn**. *Arch Argent Pediatr* 2018, **116**(4):S77-S81.
15. Noble LM, Okogbule-Wonodi AC, Young MA: **ABM Clinical Protocol #12: Transitioning the breastfeeding preterm infant from the neonatal intensive care unit to home, revised 2018**. *Breastfeed Med* 2018, **13**(4):230-236.
16. Reichert J, Eulerich-Gyamerah S, Poets C, Kribs A, Roth B, Kuhn T, Rossi R, Bindt C, von der Wense A, Rudiger M: **Psychological sociomedical care in neonatology**. *Monatsschr Kinderheilkd* 2014, **162**(11):1010-1017.
17. Attfield E, Swankhuizen MP, Bruchet N, Slavik R, Gorman SK: **Improving the clinical pharmacist handover process in the intensive care unit with a pharmacotherapy-specific tool: The I-HAPPY study**. *Can J Hosp Pharm* 2018, **71**(2):111-118.
18. Busico M, das Neves A, Carini F, Pedace M, Villalba D, Foster C, Garcia Urrutia J, Garbarini M, Jereb S, Sacha V *et al*: **Follow-up program after intensive care unit discharge**. *Med Instensiva* 2019, **43**(4):243-254.
19. Guest M: **Patient transfer from the intensive care unit to a general ward**. *Nurs Stand* 2017, **32**(10):45-51.
20. Hall W, Keane P, Wang S, Debell F, Allana A, Karia P: **Intensive care discharges: Improving the quality of clinical handover through changes to discharge documentation**. *BMJ Qual Improv Rep* 2015, **4**(1).
21. Nates JL, Nunnally M, Kleinpell R, Blosser S, Goldner J, Birriel B, Fowler CS, Byrum D, Miles WS, Bailey H *et al*: **ICU admission, discharge, and triage guidelines: A framework to enhance clinical operations, development of institutional oolicies, and further research**. *Crit Care Med* 2016, **44**(8):1553-1602.
22. Halvorson S, Wheeler B, Willis M, Watters J, Eastman J, O'Donnell R, Merkel M: **A multidisciplinary initiative to standardize intensive care to acute care transitions**. *Int J Qual Health Care* 2016, **28**(5):615-625.
23. Heselmans A, Krieken J, Cootjans S, Nagels K, Filliers D, Dillen K, De Broe S, Ramaekers D: **Medication review by a clinical pharmacist at the transfer point from ICU to ward: A randomized controlled trial**. *J Clin Pharm Ther* 2015, **40**(5):578-583.
24. Hoffman RL, Saucier J, Dasani S, Collins T, Holena DN, Fitzpatrick M, Tsypenyuk B, Martin ND: **Development and implementation of a risk identification tool to facilitate critical care transitions for high-risk surgical patients**. *Int J Qual Health Care* 2017, **29**(3):412-419.
25. Martin ND, Pisa MA, Collins TA, Robertson MP, Sicoutris CP, Bushan N, Saucier J, Martin A, Reilly PM, Lane-Fall M *et al*: **Advanced practitioner-driven critical care outreach to reduce intensive care unit readmission mortality**. *Int J Acad Med* 2015, **1**(1):3-8.
26. Sheth S, McCarthy E, Kipps AK, Wood M, Roth SJ, Sharek PJ, Shin AY: **Changes in efficiency and safety culture after integration of an I-PASS-supported handoff process**. *Pediatrics* 2016, **137**(2):e20150166.
27. Feehan K, Kehinde F, Sachs K, Mossabeb R, Berhane Z, Pachter LM, Brody S, Turchi RM: **Development of a multidisciplinary medical home program for NICU graduates**. *Matern Child Health J* 2020, **24**(1):11-21.
28. Messing J: **Improving handover from intensive care to ward medical teams with simple changes to paperwork**. *BMJ Qual Improv Rep* 2015, **4**(1).
29. Lee S, Oh H, Suh Y, Seo W: **A tailored relocation stress intervention programme for family caregivers of patients transferred from a surgical intensive care unit to a general ward**. *J Clin Nurs* 2017, **26**(5-6):784-794.

30. Baker CD, Martin S, Thrasher J, Moore HM, Baker J, Abman SH, Gien J: **A standardized discharge process decreases length of stay for ventilator-dependent children**. *Pediatrics* 2016, **137**(4).
31. Chen Y, Zhang J, Bai J: **Effect of an educational intervention on parental readiness for premature infant discharge from the neonatal intensive care units**. *J Adv Nurs* 2016, **72**(1):135-146.
32. Raines DA: **Simulation as part of discharge teaching for parents of infants in the neonatal intensive care unit**. *Am J Matern Child Nurs* 2017, **42**(2):95-100.
33. Walter L, Robb M: **Promoting discharge readiness through staff education: A family-centered approach**. *J Nurses Prof Dev* 2019, **35**(3):132-136.
34. Fabes J, Seligman W, Barrett C, McKechnie S, Griffiths J: **Does the implementation of a novel intensive care discharge risk score and nurse-led inpatient review tool improve outcome? A prospective cohort study in two intensive care units in the UK**. *BMJ Open* 2017, **7**(12):e018322.
35. Goldstein A, Shahar Y, Orenbuch E, Cohen MJ: **Evaluation of an automated knowledge-based textual summarization system for longitudinal clinical data, in the intensive care domain**. *Artif Intell Med* 2017, **82**:20-33.
36. Buck CO, Tucker R, Vohr B, McGowan EC: **Predictors of parenting readiness in fathers of high-risk infants in the neonatal intensive care unit**. *J Pediatr* 2020, **217**:192-195.e191.
37. Cresi F, Cocchi E, Maggiora E, Pirra A, Logrippo F, Ariotti MC, Peila C, Bertino E, Coscia A: **Pre-discharge cardiorespiratory monitoring in preterm infants.: The CORE study**. *Front Pediatr* 2020, **8**:234.
38. Ingram JC, Powell JE, Blair PS, Pontin D, Redshaw M, Manns S, Beasant L, Burden H, Johnson D, Rose C *et al*: **Does family-centred neonatal discharge planning reduce healthcare usage? A before and after study in South West England**. *BMJ Open* 2016, **6**(3):e010752.
39. Bench SD, Heelas K, White C, Griffiths P: **Providing critical care patients with a personalised discharge summary: A questionnaire survey and retrospective analysis exploring feasibility and effectiveness**. *Intensive Crit Care Nurs* 2014, **30**(2):69-76.
40. Kram BL, Schultheis JM, Kram SJ, Cox CE: **A pharmacy-based electronic handoff tool to reduce discharge prescribing of atypical antipsychotics initiated in the intensive care unit: A quality improvement initiative**. *J Pharm Pract* 2019, **32**(4):434-441.
41. Kraus S, Castellanos I, Albermann M, Schuettler C, Prokosch H-U, Staudigel M, Toddenroth D: **Using Arden Syntax for the generation of intelligent intensive care discharge letters**. *Stud Health Technol Inform* 2016, **228**:471-475.
42. Bench S, Day T, Heelas K, Hopkins P, White C, Griffiths P: **Evaluating the feasibility and effectiveness of a critical care discharge information pack for patients and their families: a pilot cluster randomised controlled trial**. *BMJ Open* 2015, **5**(11):e006852.
43. Manente L, McCluskey T, Shaw R: **Transitioning patients from the intensive care unit to the general pediatric unit: A piece of the puzzle in family-centered care**. *Pediatr Nurs* 2017, **43**(2):77-82.
44. Milton A, Schandl A, Soliman I, Joelsson-Alm E, van den Boogaard M, Wallin E, Brorsson C, Ostberg U, Latocha K, Savilampi J *et al*: **ICU discharge screening for prediction of new-onset physical disability-A multinational cohort study**. *Acta Anaesthesiol Scan* 2020, **64**(6):789-797.
45. Szubski CR, Tellez A, Klika AK, Xu M, Kattan MW, Guzman JA, Barsoum WK: **Predicting discharge to a long-term acute care hospital after admission to an intensive care unit**. *Am J Crit Care* 2014, **23**(4):e46-e53.
46. Milton A, Schandl A, Soliman IW, Meijers K, van den Boogaard M, Larsson IM, Brorsson C, Ostberg U, Oxenboll-Collet M, Savilampi J *et al*: **Development of an ICU discharge instrument predicting psychological morbidity: A multinational study**. *Intensive Care Med* 2018, **44**(12):2038-2047.
47. Carty CL, Soghier LM, Kritikos KI, Tuchman LK, Jiggetts M, Glass P, Streisand R, Fratantoni KR: **The Giving Parents Support Study: A randomized clinical trial of a parent navigator intervention to improve outcomes after neonatal intensive care unit discharge**. *Contemp Clin Trials* 2018, **70**:117-134.
