# Supplementary material for: Patient discharge from intensive care: an updated scoping review to identify tools and practices to inform high-quality care
Source: Crit Care. 2021 Dec 17;25:438. doi: 10.1186/s13054-021-03857-2 (PMC8684123; doi:10.1186/s13054-021-03857-2)
Supplement: Supplementary file 4 — Additional file 4. Article classification according to study population, describing framework elements, themes, and facilitators and barriers [file 13054_2021_3857_MOESM4_ESM.pdf]

Additional File 4 – Article classification according to study population, describing framework elements, themes, and facilitators and barriers.

Author (Year)	Study Population	Phase(s) of Care	Donabedian[a]	IoM[b]	Themes	Patient & Family Facilitators (F) & Barriers (B)	Provider Facilitators (F) & Barriers (B)	Organizational Facilitators (F) & Barriers (B)	Tool
Abitağaoğlu (2019)[1]	Adult	Follow-up	Outcome	Safe	Adverse events, readmission, and mortality following discharge; Continuity of patient care;	(B) Patient demographic and clinical characteristics;	(None)	(F) Use of best practices;	(None)
Academia (2020)[2]	Adult	Execution	Process	Efficient; Safe	Adverse events, readmission, and mortality following discharge; Medication reconciliation;	(B) Patient demographic and clinical characteristics;	(B) Lack of knowledge/ experience of provider;	(F) Use of best practices; (B) Hospital characteristics;	(None)
Agard (2019)[3]	Adult	Execution	Process	Effective; Patient centered	Patient and family needs and experiences during discharge; Availability of complete and accurate discharge information;	(F) Family engagement/ support; (F&B) (Lack of) Provider-patient communication; (B) Feelings of anxiety or embarrassment; Expectations;	(F) Critical care transition program; Knowledge/ experience of provider;	(F) Follow-up clinic or program; (B) Impact of current discharge practices; Hospital characteristics; Infrastructure;	(None)
Alberto (2014)[4]	Adult	Execution	Process	Efficient; Patient centered	Planning for discharge; Standardizing the discharge process; Critical care transition program (nurse liaison, outreach team);	(F) Discharge education; Family engagement/ support;	(F) Critical care transition program;	(F) Guidelines or policies; Education/ training of providers;	(None)
Allum (2017)[5]	Adult	Follow-up	Outcome	Effective; Patient centered	Patient and family needs and experiences during discharge; Anxiety associated with discharge; Continuity of patient care;	(F) Provider-patient communication; Discharge education; Family engagement/ support; Written communication; (F&B)	(F) Provider-provider communication; Critical care transition program;	(F) Follow-up clinic or program;	(None)

Author (Year)	Study Population	Phase(s) of Care	Donabedian[a]	IoM[b]	Themes	Patient & Family Facilitators (F) & Barriers (B)	Provider Facilitators (F) & Barriers (B)	Organizational Facilitators (F) & Barriers (B)	Tool
						Expectations; (B) Feelings of anxiety or embarrassment;			
Amass (2020)[6]	Adult	Follow-up	Outcome	Effective; Safe; Patient centered	Patient and family needs and experiences during discharge; Discharge education for patients and families; Adverse events, readmission, and mortality following discharge;	(F) Provider-patient communication; Family engagement/ support; Written communication; Patient/ family treated as a member of healthcare team; (B) Feelings of anxiety or embarrassment;	(F) Written documentation;	(F) Impact of current discharge practices;	(None)
Anthes (2013)[7]	Adult	Execution	Process	Safe	Availability of complete and accurate discharge information; Adverse events, readmission, and mortality following discharge; Medication reconciliation;	(None)	(F) Written documentation;	(F) Use of best practices; Tools to facilitate discharge;	(None)
Azevedo (2015)[8]	Adult	Execution	Structure; Outcome	Safe	Planning for discharge; Adverse events, readmission, and mortality following discharge; Timeliness of discharge (time of day, delay);	(B) Patient demographic and clinical characteristics;	(None)	(F&B) Admission location before ICU; (B) Time of discharge;	(None)
Bagshaw (2020)[9]	Adult	Execution	Process; Structure; Outcome	Efficient; Safe; Timely	Adverse events, readmission, and mortality following discharge; Timeliness of discharge (time of	(None)	(None)	(F) Discharge location; (B) Limited ICU and ward resources; Impact of	(None)

Author (Year)	Study Population	Phase(s) of Care	Donabedian[a]	IoM[b]	Themes	Patient & Family Facilitators (F) & Barriers (B)	Provider Facilitators (F) & Barriers (B)	Organizational Facilitators (F) & Barriers (B)	Tool
					day, delay); Resource use during discharge;			current discharge practices; Costs of healthcare provided; Delay in discharge; Hospital or ICU capacity;	
Baldwin (2013)[10]	Adult	Follow-up	Outcome	Safe	Adverse events, readmission, and mortality following discharge;	(F&B) Patient demographic and clinical characteristics; (B) Physical and psychological effects of illness; ICU or hospital length of stay;	(B) Lack of knowledge/ experience of provider;	(None)	(None)
Balshi (2020)[11]	Adult	Execution; Follow-up	Outcome	Safe	Adverse events, readmission, and mortality following discharge;	(B) Patient demographic and clinical characteristics;	(None)	(None)	(None)
Basmaji (2019)[12]	Adult	Execution; Follow-up	Process; Outcome	Safe	Adverse events, readmission, and mortality following discharge;	(F&B) Patient demographic and clinical characteristics;	(None)	(F&B) Discharge location;	(None)
Bench (2013)[13]	Adult	Planning; Execution; Follow-up	Process; Outcome	Safe; Patient centered	Patient and family needs and experiences during discharge; Availability of complete and accurate discharge information; Discharge education for patients and families; Planning for discharge; Standardizing the discharge process;	(F) Discharge education; Written communication; (B) Feelings of anxiety or embarrassment;	(None)	(None)	(None)

Author (Year)	Study Population	Phase(s) of Care	Donabedian[a]	IoM[b]	Themes	Patient & Family Facilitators (F) & Barriers (B)	Provider Facilitators (F) & Barriers (B)	Organizational Facilitators (F) & Barriers (B)	Tool
					Anxiety associated with discharge;				
Bench (2014)[14]	Adult	Execution; Follow-up	Process; Structure	Effective; Patient centered	Patient and family needs and experiences during discharge; Availability of complete and accurate discharge information; Planning for discharge; Anxiety associated with discharge; Continuity of patient care;	(F) Provider-patient communication; Discharge education; Family engagement/ support; (B) Feelings of anxiety or embarrassment; Expectations; Physical and psychological effects of illness;	(F) Provider-provider communication; Knowledge/ experience of provider; Clear roles and responsibilities;	(B) Lack of education/ training of providers;	Discharge letter
Bench (2015)[15]	Adult	Execution	Process; Structure	Patient centered	Patient and family needs and experiences during discharge; Availability of complete and accurate discharge information; Discharge education for patients and families;	(F) Provider-patient communication; Discharge education; Written communication;	(F) Collaboration between ICU and ward; (B) Workload;	(None)	Transfer brochure
Bench (2016)[16]	Adult	Execution	Process; Structure	Efficient	Patient and family needs and experiences during discharge; Discharge education for patients and families; Resource use during discharge;	(F) Provider-patient communication; Discharge education; Family engagement/ support;	(None)	(F) Impact of current discharge practices;	(None)
Bloom (2019)[17]	Adult	Execution; Follow-up	Process; Outcome	Effective; Safe	Adverse events, readmission, and mortality following discharge; Critical care transition program (nurse liaison, outreach team);	(F) Discharge education;	(F) Critical care transition program;	(F&B) Discharge location;	(None)

Author (Year)	Study Population	Phase(s) of Care	Donabedian[a]	IoM[b]	Themes	Patient & Family Facilitators (F) & Barriers (B)	Provider Facilitators (F) & Barriers (B)	Organizational Facilitators (F) & Barriers (B)	Tool
Bose (2019)[18]	Adult	Execution; Follow-up	Outcome	Efficient; Patient centered ; Timely	Adverse events, readmission, and mortality following discharge; Timeliness of discharge (time of day, delay);	(None)	(F) Provider-provider communication;	(F) Impact of current discharge practices; (B) Limited ICU and ward resources; Delay in discharge; Hospital or ICU capacity;	(None)
Bosma (2018)[19]	Adult	Execution	Process	Efficient; Safe	Planning for discharge; Resource use during discharge; Medication reconciliation;	(None)	(F) Provider-provider communication; Collaboration between ICU and ward;	(F) Use of best practices; Impact of current discharge practices; (B) Costs of healthcare provided;	(None)
Bosma (2019)[20]	Adult	Follow-up	Process; Outcome	Efficient; Safe; Patient centered	Availability of complete and accurate discharge information; Adverse events, readmission, and mortality following discharge; Continuity of patient care; Medication reconciliation;	(None)	(F) Clear roles and responsibilities;	(F&B) Impact of current discharge practices;	(None)
Boyd (2018)[21]	Adult	Execution	Structure	Effective; Efficient; Patient centered	Patient and family needs and experiences during discharge; Availability of complete and accurate discharge information; Standardizing the discharge process;	(F) Provider-patient communication; Family engagement/ support; Written communication;	(F) Provider-provider communication; Collaboration between ICU and ward; Written documentation;	(F) Tools to facilitate discharge;	(None)

Author (Year)	Study Population	Phase(s) of Care	Donabedian[a]	IoM[b]	Themes	Patient & Family Facilitators (F) & Barriers (B)	Provider Facilitators (F) & Barriers (B)	Organizational Facilitators (F) & Barriers (B)	Tool
					Medication reconciliation;				
Brown (2013)[22]	Adult	Follow-up	Outcome	Safe	Adverse events, readmission, and mortality following discharge; Timeliness of discharge (time of day, delay);	(F&B) Patient demographic and clinical characteristics;	(None)	(B) Time of discharge; Impact of current discharge practices;	(None)
Brown (2018)[23]	Adult	Execution	Process	Patient centered	Availability of complete and accurate discharge information; Evaluating patient readiness for discharge; Continuity of patient care;	(F&B) (Lack of) Provider-patient communication;	(F) Provider-provider communication; (B) Lack of knowledge/ experience of provider;	(None)	(None)
Chaboyer (2012)[24]	Adult	Execution	Process	Efficient; Timely	Standardizing the discharge process; Adverse events, readmission, and mortality following discharge; Timeliness of discharge (time of day, delay); Evaluating patient readiness for discharge; Resource use during discharge;	(F) Patient demographic and clinical characteristics;	(F) Provider-provider communication; Collaboration between ICU and ward; Written documentation;	(B) Time of discharge; Delay in discharge;	(None)
Chatterjee (2019)[25]	Adult	Execution	Process; Outcome	Safe; Timely	Adverse events, readmission, and mortality following discharge; Timeliness of discharge (time of day, delay);	(B) Patient demographic and clinical characteristics; Physical and psychological effects of illness;	(B) Workload;	(F&B) Time of discharge; (B) Limited ICU and ward resources; Staffing;	(None)
Choi (2016)[26]	Adult	Follow-up	Outcome	Effective; Efficient	Adverse events, readmission, and mortality following discharge; Critical care transition program	(F&B) Patient demographic and clinical characteristics;	(F) Critical care transition program; Knowledge/	(None)	(None)

Author (Year)	Study Population	Phase(s) of Care	Donabedian[a]	IoM[b]	Themes	Patient & Family Facilitators (F) & Barriers (B)	Provider Facilitators (F) & Barriers (B)	Organizational Facilitators (F) & Barriers (B)	Tool
					(nurse liaison, outreach team);		experience of provider;		
Choi (2018)[27]	Adult	Follow-up	Outcome	Patient centered	Patient and family needs and experiences during discharge; Discharge education for patients and families; Planning for discharge; Anxiety associated with discharge;	(F) Family engagement/ support; (F&B) Expectations; (B) Feelings of anxiety or embarrassment;	(None)	(None)	(None)
Choi (2020)[28]	Adult	Execution	Process	Patient centered	Patient and family needs and experiences during discharge; Anxiety associated with discharge;	(F) Discharge education; Family engagement/ support; (B) Patient demographic and clinical characteristics; Feelings of anxiety or embarrassment; Expectations; Physical and psychological effects of illness;	(None)	(None)	(None)
Churpek (2013)[29]	Adult	Execution	Outcome	Effective; Efficient; Timely	Adverse events, readmission, and mortality following discharge; Timeliness of discharge (time of day, delay); Resource use during discharge;	(B) Patient demographic and clinical characteristics;	(None)	(B) Impact of current discharge practices; Delay in discharge;	(None)
Coombs (2015)[30]	Adult	Planning; Execution	Process; Structure	Patient centered	Patient and family needs and experiences during discharge; Planning for discharge; Continuity of patient care; Autonomy;	(F) Provider-patient communication;	(F) Provider-provider communication;	(F) Follow-up clinic or program;	(None)

Author (Year)	Study Population	Phase(s) of Care	Donabedian[a]	IoM[b]	Themes	Patient & Family Facilitators (F) & Barriers (B)	Provider Facilitators (F) & Barriers (B)	Organizational Facilitators (F) & Barriers (B)	Tool
Coombs (2017)[31]	Adult	Evaluation	Process	Patient centered	Planning for discharge; Evaluating patient readiness for discharge;	(F) Patient demographic and clinical characteristics;	(None)	(F) Impact of current discharge practices;	(None)
Coon (2015)[32]	Adult	Execution	Process	Efficient	Adverse events, readmission, and mortality following discharge; Continuity of patient care; Medication reconciliation;	(None)	(F) Provider-provider communication; Written documentation; (B) Workload;	(None)	Guideline/ checklist
Correa (2018)[33]	Adult	Planning; Follow-up	Process	Effective	Evaluating patient readiness for discharge;	(F&B) Patient demographic and clinical characteristics;	(F) Clinical judgement or decision making;	(None)	(None)
Coughlin (2018)[34]	Adult	Execution; Follow-up	Process; Outcome	Safe; Timely	Adverse events, readmission, and mortality following discharge; Timeliness of discharge (time of day, delay);	(None)	(None)	(B) Time of discharge; Impact of current discharge practices; Discharge location;	(None)
Corner (2014)[35]	Adult	Follow-up	Outcome	Efficient; Safe	Adverse events, readmission, and mortality following discharge;	(B) Patient demographic and clinical characteristics;	(F) Clear roles and responsibilities;	(None)	(None)
Cypress (2013)[36]	Adult	Execution	Process	Effective	Patient and family needs and experiences during discharge;	(F) Discharge education; Expectations; (B) Patient demographic and clinical characteristics; Feelings of anxiety or embarrassment; Physical and psychological effects of illness;	(F) Collaboration between ICU and ward;	(None)	(None)

Author (Year)	Study Population	Phase(s) of Care	Donabedian[a]	IoM[b]	Themes	Patient & Family Facilitators (F) & Barriers (B)	Provider Facilitators (F) & Barriers (B)	Organizational Facilitators (F) & Barriers (B)	Tool
D'Angelo (2019)[37]	Adult	Execution	Process	Efficient; Safe	Standardizing the discharge process; Medication reconciliation;	(B) ICU or hospital length of stay;	(F) Clinical judgement or decision making;	(F) Guidelines or policies;	Guideline/ checklist
Darlington (2015)[38]	Adult	Evaluation; Planning	Process	Effective; Patient centered	Planning for discharge; Autonomy;	(F&B) Patient demographic and clinical characteristics;	(None)	(F) Use of best practices; Tools to facilitate discharge; (B) Impact of current discharge practices;	(None)
de Grood (2018)[39]	Adult	Planning; Execution	Structure	Efficient; Safe; Patient centered	Availability of complete and accurate discharge information; Discharge education for patients and families; Planning for discharge; Timeliness of discharge (time of day, delay); Resource use during discharge; Continuity of patient care;	(F) Provider-patient communication; Discharge education;	(F) Provider-provider communication; Critical care transition program; Collaboration between ICU and ward; Knowledge/ experience of provider; Written documentation; Clear roles and responsibilities; Provider leadership;	(F) Tools to facilitate discharge; (B) Limited ICU and ward resources; Time of discharge; Delay in discharge;	(None)
Detsky (2015)[40]	Adult	Execution	Process; Structure; Outcome	Effective; Safe	Availability of complete and accurate discharge information; Standardizing the discharge process; Adverse events, readmission, and mortality following	(None)	(F) Provider-provider communication; Collaboration between ICU and ward;	(F) Tools to facilitate discharge; (B) Impact of current discharge practices;	(None)

Author (Year)	Study Population	Phase(s) of Care	Donabedian[a]	IoM[b]	Themes	Patient & Family Facilitators (F) & Barriers (B)	Provider Facilitators (F) & Barriers (B)	Organizational Facilitators (F) & Barriers (B)	Tool
					discharge; Resource use during discharge; Continuity of patient care;				
Ebrahimian (2018)[41]	Adult	Evaluation	Process; Outcome	Effective; Safe	Planning for discharge; Standardizing the discharge process; Adverse events, readmission, and mortality following discharge; Evaluating patient readiness for discharge;	(F&B) Patient demographic and clinical characteristics;	(F) Clinical judgement or decision making;	(F) Tools to facilitate discharge;	(None)
Edenharter (2019)[42]	Adult	Execution	Process; Structure	Efficient; Timely	Timeliness of discharge (time of day, delay); Resource use during discharge;	(None)	(None)	(B) Limited ICU and ward resources; Impact of current discharge practices; Costs of healthcare provided; Delay in discharge; Hospital characteristics; Hospital or ICU capacity;	(None)
El Hadidi (2020)[43]	Adult	Execution	Process	Safe	Medication reconciliation;	(F) Patient demographic and clinical characteristics;	(F) Multidisciplinary team;	(F) Guidelines or policies; Use of best practices; (B) Impact of current discharge practices;	(None)
Enger (2018)[44]	Adult	Execution; Follow-up	Process; Structure; Outcome	Effective; Safe	Availability of complete and accurate discharge	(B) Feelings of anxiety or embarrassment;	(F) Provider-provider communication;	(B) Limited ICU and ward resources; (B)	(None)

Author (Year)	Study Population	Phase(s) of Care	Donabedian[a]	IoM[b]	Themes	Patient & Family Facilitators (F) & Barriers (B)	Provider Facilitators (F) & Barriers (B)	Organizational Facilitators (F) & Barriers (B)	Tool
					information; Planning for discharge; Anxiety associated with discharge; Continuity of patient care;		Collaboration between ICU and ward; Knowledge/ experience of provider; (B) Workload; Provider anxiety;	Impact of current discharge practices;	
Fabes (2017)[45]	Adult	Planning; Execution; Follow-up	Structure; Outcome	Efficient; Safe	Planning for discharge; Standardizing the discharge process; Adverse events, readmission, and mortality following discharge; Evaluating patient readiness for discharge;	(B) Patient demographic and clinical characteristics;	(None)	(F) Guidelines or policies; Tools to facilitate discharge; (B) Impact of current discharge practices;	Discharge assessment
Fergusson (2020)[46]	Adult	Execution; Follow-up	Structure; Outcome	Effective; Safe	Availability of complete and accurate discharge information; Adverse events, readmission, and mortality following discharge;	(None)	(B) Workload;	(F&B) Time of discharge; (B) Hospital or ICU capacity;	(None)
Forster (2020)[47]	Adult	Follow-up	Outcome	Safe; Timely	Availability of complete and accurate discharge information; Adverse events, readmission, and mortality following discharge; Timeliness of discharge (time of day, delay);	(None)	(None)	(B) Time of discharge; Delay in discharge; Hospital characteristics; Hospital or ICU capacity;	(None)
Gantner (2014)[48]	Adult	Execution; Follow-up	Structure; Outcome	Safe; Timely	Adverse events, readmission, and mortality following discharge; Timeliness	(B) Patient demographic and clinical characteristics;	(None)	(F) Impact of current discharge practices; (F&B)	(None)

Author (Year)	Study Population	Phase(s) of Care	Donabedian[a]	IoM[b]	Themes	Patient & Family Facilitators (F) & Barriers (B)	Provider Facilitators (F) & Barriers (B)	Organizational Facilitators (F) & Barriers (B)	Tool
					of discharge (time of day, delay);			Time of discharge; (B) Costs of healthcare provided; Delay in discharge;	
Garland and Connors (2013)[49]	Adult	Follow-up	Outcome	Safe; Timely	Adverse events, readmission, and mortality following discharge; Timeliness of discharge (time of day, delay);	(B) Patient demographic and clinical characteristics;	(None)	(B) Delay in discharge; Discharge location; Hospital characteristics;	(None)
Gilbert (2017)[50]	Adult	Execution; Follow-up	Outcome	Safe	Continuity of patient care; Medication reconciliation;	(None)	(None)	(None)	(None)
Gimpel (2019)[51]	Adult	Execution; Follow-up	Outcome	Effective; Safe; Timely	Availability of complete and accurate discharge information; Planning for discharge; Timeliness of discharge (time of day, delay);	(None)	(F) Collaboration between ICU and ward; Clinical judgement or decision making;	(B) Delay in discharge;	(None)
Goldstein (2017)[52]	Adult	Planning; Execution	Structure	Efficient; Safe	Availability of complete and accurate discharge information; Planning for discharge; Resource use during discharge;	(None)	(F&B) (Lack of) Provider-provider communication;	(None)	Discharge assessment
Gotur (2018)[53]	Adult	Evaluation; Follow-up	Outcome	Efficient; Timely	Adverse events, readmission, and mortality following discharge; Evaluating patient readiness for discharge;	(B) Patient demographic and clinical characteristics;	(None)	(None)	(None)
Häggström (2014)[54]	Adult	Execution	Process	Patient centered	Patient and family needs and experiences during	(F) Provider-patient communication;	(F) Provider-provider communication;	(None)	(None)

Author (Year)	Study Population	Phase(s) of Care	Donabedian[a]	IoM[b]	Themes	Patient & Family Facilitators (F) & Barriers (B)	Provider Facilitators (F) & Barriers (B)	Organizational Facilitators (F) & Barriers (B)	Tool
					discharge; Availability of complete and accurate discharge information; Discharge education for patients and families;	Discharge education; Family engagement/ support; Written communication; Expectations;	Collaboration between ICU and ward; (B) Workload;		
Häggström (2018)[55]	Adult	Execution	Process; Structure	Effective; Safe; Patient centered	Availability of complete and accurate discharge information; Critical care transition program (nurse liaison, outreach team); Evaluating patient readiness for discharge; Continuity of patient care; Discharge education for providers;	(None)	(F) Provider-provider communication; Critical care transition program; Collaboration between ICU and ward; Written documentation; Clinical judgement or decision making; Clear roles and responsibilities;	(F) Impact of current discharge practices; (B) Limited ICU and ward resources;	(None)
Hajalizadeh (2020)[56]	Adult	Planning; Execution; Follow-up	Process; Structure	Patient centered	Patient and family needs and experiences during discharge; Discharge education for patients and families; Autonomy;	(F) Provider-patient communication; Discharge education; Family engagement/ support;	(None)	(None)	(None)
Halvorson (2016)[57]	Adult	Execution	Process; Structure	Efficient; Timely	Standardizing the discharge process; Timeliness of discharge (time of day, delay); Resource use during discharge;	(None)	(None)	(F) Guidelines or policies; Tools to facilitate discharge; (B) Impact of current discharge practices; Delay in discharge;	Transfer tool

**Author (Year)**	**Study Population**	**Phase(s) of Care**	**Donabedian[a]**	**IoM[b]**	**Themes**	**Patient & Family Facilitators (F) & Barriers (B)**	**Provider Facilitators (F) & Barriers (B)**	**Organizational Facilitators (F) & Barriers (B)**	**Tool**
Haraldsson (2015)[58]	Adult	Follow-up	Outcome	Patient centered	Anxiety associated with discharge; Continuity of patient care;	(B) Anxiety; (B) Feelings of anxiety or embarrassment; Physical and psychological effects of illness;	(None)	(None)	(None)
Harlan (2020)[59]	Adult	Execution; Follow-up	Outcome	Effective; Safe; Timely	Adverse events, readmission, and mortality following discharge; Evaluating patient readiness for discharge;	(B) Patient demographic and clinical characteristics;	(F) Knowledge/ experience of provider; Clinical judgement or decision making;	(F) Impact of current discharge practices; (B) Hospital or ICU capacity;	(None)
Herling (2019)[60]	Adult	Follow-up	Outcome	Patient centered	Continuity of patient care; Autonomy;	(F) Excited, joyous to be leaving ICU; (B) Feelings of anxiety or embarrassment;	(None)	(None)	(None)
Herve (2020)[61]	Adult	Evaluation; Planning; Execution; Follow-up	Structure; Outcome	Effective; Efficient; Timely	Patient and family needs and experiences during discharge; Availability of complete and accurate discharge information; Planning for discharge; Standardizing the discharge process; Adverse events, readmission, and mortality following discharge; Continuity of patient care; Medication reconciliation;	(F) Family engagement/ support; Written communication;	(F) Provider-provider communication; Written documentation; Multidisciplinary team; (B) Lack of knowledge/ experience of provider;	(F) Guidelines or policies; Use of best practices; Impact of current discharge practices; Tools to facilitate discharge; Follow-up clinic or program; (B) Limited ICU and ward resources; Time of discharge; Delay in discharge; Discharge location; Staffing; Reduction in	(None)

Author (Year)	Study Population	Phase(s) of Care	Donabedian[a]	IoM[b]	Themes	Patient & Family Facilitators (F) & Barriers (B)	Provider Facilitators (F) & Barriers (B)	Organizational Facilitators (F) & Barriers (B)	Tool
								technology and monitoring;	
Heselmans (2015)[62]	Adult	Execution	Process; Outcome	Effective; Safe	Adverse events, readmission, and mortality following discharge; Medication reconciliation;	(None)	(F) Clear roles and responsibilities;	(None)	Transfer tool
Hoffman (2017)[63]	Adult	Planning; Execution; Follow-up	Process; Outcome	Effective; Safe	Planning for discharge; Standardizing the discharge process; Adverse events, readmission, and mortality following discharge;	(None)	(F) Provider-provider communication; Collaboration between ICU and ward; Written documentation;	(F) Guidelines or policies; Tools to facilitate discharge;	Transfer tool
Holland (2012)[64]	Adult	Planning	Process	Patient centered	Availability of complete and accurate discharge information; Planning for discharge; Standardizing the discharge process;	(F) Patient demographic and clinical characteristics;	(F) Provider-provider communication; Written documentation;	(None)	(None)
James (2013)[65]	Adult	Evaluation; Execution	Process; Structure	Effective; Efficient	Availability of complete and accurate discharge information; Standardizing the discharge process; Anxiety associated with discharge; Critical care transition program (nurse liaison, outreach team); Discharge education for providers;	(None)	(F) Provider-provider communication; Critical care transition program; Collaboration between ICU and ward; Clinical judgement or decision making; Clear roles and responsibilities; (B) Provider anxiety;	(None)	(None)
Jensen (2019)[66]	Adult	Follow-up	Outcome	Effective; Safe	Adverse events, readmission, and	(None)	(F) Critical care transition	(None)	(None)

Author (Year)	Study Population	Phase(s) of Care	Donabedian[a]	IoM[b]	Themes	Patient & Family Facilitators (F) & Barriers (B)	Provider Facilitators (F) & Barriers (B)	Organizational Facilitators (F) & Barriers (B)	Tool
					mortality following discharge; Critical care transition program (nurse liaison, outreach team); Continuity of patient care;		program; Collaboration between ICU and ward; Written documentation; (F&B) (Lack of) Provider-provider communication; (Lack of) Knowledge/ experience of provider;		
Jeong (2019)[67]	Adult	Follow-up	Outcome	Effective; Safe	Adverse events, readmission, and mortality following discharge;	(B) Patient demographic and clinical characteristics; ICU or hospital length of stay;	(None)	(B) Time of discharge; Impact of current discharge practices;	(None)
Jolley (2019)[68]	Adult	Execution; Follow-up	Outcome	Effective; Safe	Adverse events, readmission, and mortality following discharge; Continuity of patient care;	(B) Patient demographic and clinical characteristics;	(F) Clinical judgement or decision making;	(F&B) Discharge location;	(None)
Jonasdottir (2015)[69]	Adult	Follow-up	Outcome	Efficient	Critical care transition program (nurse liaison, outreach team); Continuity of patient care;	(None)	(F) Critical care transition program;	(None)	(None)
Jonasdottir (2018)[70]	Adult	Follow-up	Outcome	Effective; Efficient	Adverse events, readmission, and mortality following discharge; Critical care transition program (nurse liaison, outreach team); Continuity of patient care;	(B) Patient demographic and clinical characteristics;	(F) Critical care transition program; Collaboration between ICU and ward; (B) Workload; Provider anxiety;	(None)	(None)

Author (Year)	Study Population	Phase(s) of Care	Donabedian[a]	IoM[b]	Themes	Patient & Family Facilitators (F) & Barriers (B)	Provider Facilitators (F) & Barriers (B)	Organizational Facilitators (F) & Barriers (B)	Tool
Kastrup (2013)[71]	Adult	Follow-up	Outcome	Safe; Equal	Adverse events, readmission, and mortality following discharge;	(B) Patient demographic and clinical characteristics;	(None)	(F) Admission location before ICU;	(None)
Kheir (2016)[72]	Adult	Follow-up	Process; Outcome	Effective; Safe	Adverse events, readmission, and mortality following discharge; Critical care transition program (nurse liaison, outreach team);	(None)	(F) Critical care transition program;	(None)	(None)
King (2019)[73]	Adult	Planning; Execution; Follow-up	Process; Outcome	Patient centered ; Timely	Patient and family needs and experiences during discharge; Availability of complete and accurate discharge information; Discharge education for patients and families; Planning for discharge; Anxiety associated with discharge; Continuity of patient care;	(F) Discharge education; Family engagement/ support; (B) Feelings of anxiety or embarrassment; Expectations; Physical and psychological effects of illness;	(None)	(F) Impact of current discharge practices; Discharge location;	(None)
Klepstad (2019)[74]	Adult	Follow-up	Outcome	Safe	Adverse events, readmission, and mortality following discharge;	(B) Patient demographic and clinical characteristics;	(None)	(None)	(None)
Kohn (2019)[75]	Adult	Execution; Follow-up	Outcome	Safe; Timely	Timeliness of discharge (time of day, delay); Resource use during discharge; Continuity of patient care;	(None)	(None)	(B) Limited ICU and ward resources; Delay in discharge; Discharge location;	(None)
Kram (2019)[76]	Adult	Planning	Structure; Outcome	Efficient; Safe;	Planning for discharge; Resource use during discharge;	(None)	(F) Written documentation;	(F) Tools to facilitate discharge;	Discharge letter

Author (Year)	Study Population	Phase(s) of Care	Donabedian[a]	IoM[b]	Themes	Patient & Family Facilitators (F) & Barriers (B)	Provider Facilitators (F) & Barriers (B)	Organizational Facilitators (F) & Barriers (B)	Tool
				Patient centered	Continuity of patient care; Medication reconciliation;				
Kram (2015)[77]	Adult	Follow-up	Outcome	Effective; Safe	Adverse events, readmission, and mortality following discharge; Medication reconciliation;	(None)	(F) Clear roles and responsibilities;	(None)	(None)
Kuang-Ming (2020)[78]	Adult	Follow-up	Outcome	Patient centered ; Timely	Adverse events, readmission, and mortality following discharge; Timeliness of discharge (time of day, delay); Continuity of patient care;	(B) Patient demographic and clinical characteristics; Feelings of anxiety or embarrassment;	(None)	(F&B) Hospital characteristics; (B) Time of discharge;	(None)
Küçükosman (2020)[79]	Adult	Follow-up	Outcome	Safe	Adverse events, readmission, and mortality following discharge; Resource use during discharge;	(B) Patient demographic and clinical characteristics;	(None)	(None)	(None)
Lam (2017)[80]	Adult	Follow-up	Outcome	Patient centered	Patient and family needs and experiences during discharge; Adverse events, readmission, and mortality following discharge;	(F) Family engagement/ support; Expectations;	(F) Clinical judgement or decision making;	(F&B) Discharge location;	(None)
Lau (2018)[81]	Adult	Execution	Process; Structure	Efficient; Patient centered	Standardizing the discharge process; Timeliness of discharge (time of day, delay);	(None)	(F) Collaboration between ICU and ward;	(F) Guidelines or policies; (B) Delay in discharge;	(None)
Lau (2018)[82]	Adult	Follow-up	Outcome	Safe	Adverse events, readmission, and mortality following discharge;	(B) Patient demographic and clinical characteristics; ICU or hospital length of stay;	(None)	(B) Discharge location;	(None)

Author (Year)	Study Population	Phase(s) of Care	Donabedian[a]	IoM[b]	Themes	Patient & Family Facilitators (F) & Barriers (B)	Provider Facilitators (F) & Barriers (B)	Organizational Facilitators (F) & Barriers (B)	Tool
Lau (2018)[83]	Adult	Follow-up	Structure; Outcome	Safe	Planning for discharge; Adverse events, readmission, and mortality following discharge; Evaluating patient readiness for discharge;	(None)	(F) Critical care transition program;	(F) Follow-up clinic or program; (F&B) Discharge location;	(None)
Lee (2017)[84]	Adult	Planning	Process	Patient centered	Patient and family needs and experiences during discharge; Availability of complete and accurate discharge information; Discharge education for patients and families; Anxiety associated with discharge;	(F) Discharge education; Written communication; (B) Feelings of anxiety or embarrassment;	(None)	(None)	Educational tool
Lekwijit (2020)[85]	Adult	Planning; Follow-up	Outcome	Efficient	Planning for discharge; Adverse events, readmission, and mortality following discharge; Continuity of patient care;	(B) Patient demographic and clinical characteristics;	(None)	(F) Use of best practices; Discharge location;	(None)
Lin (2017)[86]	Adult	Execution	Process; Structure	Effective; Efficient	Planning for discharge;	(F) Patient demographic and clinical characteristics;	(F) Provider-provider communication; Clinical judgement or decision making;	(F) Education/ training of providers;	(None)
Lin (2019)[87]	Adult	Follow-up	Outcome	Safe; Timely	Adverse events, readmission, and mortality following discharge; Critical care transition program	(None)	(None)	(F) Tools to facilitate discharge;	(None)

Author (Year)	Study Population	Phase(s) of Care	Donabedian[a]	IoM[b]	Themes	Patient & Family Facilitators (F) & Barriers (B)	Provider Facilitators (F) & Barriers (B)	Organizational Facilitators (F) & Barriers (B)	Tool
					(nurse liaison, outreach team);				
Major (2019)[88]	Adult	Follow-up	Process; Outcome	Patient centered	Patient and family needs and experiences during discharge; Availability of complete and accurate discharge information;	(F) Family engagement/ support; (F&B) (Lack of) Provider-patient communication; (B) Feelings of anxiety or embarrassment;	(None)	(B) Discharge location;	(None)
Martin (2020)[89]	Adult	Planning; Follow-up	Process; Outcome	Patient centered	Planning for discharge;	(B) Patient demographic and clinical characteristics;	(None)	(F) Discharge location; Admission location before ICU; (B) Hospital or ICU capacity;	(None)
Martin (2015)[90]	Adult	Execution; Follow-up	Structure; Outcome	Effective; Safe	Adverse events, readmission, and mortality following discharge; Critical care transition program (nurse liaison, outreach team);	(None)	(F) Provider-provider communication; Critical care transition program; Written documentation;	(F) Tools to facilitate discharge; (F&B) Impact of current discharge practices;	Transfer tool
McCairn (2014)[91]	Adult	Execution; Follow-up	Process; Outcome	Safe; Timely	Patient and family needs and experiences during discharge; Anxiety associated with discharge; Timeliness of discharge (time of day, delay);	(B) Feelings of anxiety or embarrassment;	(None)	(B) Time of discharge;	(None)
McWilliams (2019)[92]	Adult	Evaluation; Planning; Follow-up	Process; Outcome	Effective; Safe; Patient centered	Planning for discharge; Adverse events, readmission, and mortality following discharge;	(F&B) Patient demographic and clinical characteristics;	(F) Clinical judgement or decision making;	(F) Tools to facilitate discharge;	(None)

Author (Year)	Study Population	Phase(s) of Care	Donabedian[a]	IoM[b]	Themes	Patient & Family Facilitators (F) & Barriers (B)	Provider Facilitators (F) & Barriers (B)	Organizational Facilitators (F) & Barriers (B)	Tool
					Evaluating patient readiness for discharge;				
Milton (2020)[93]	Adult	Execution; Follow-up	Outcome	Patient centered	Adverse events, readmission, and mortality following discharge; Evaluating patient readiness for discharge; Resource use during discharge;	(F&B) Patient demographic and clinical characteristics; Socioeconomic factors;	(F) Clinical judgement or decision making;	(F) Use of best practices; Tools to facilitate discharge;	Prediction tool
Milton (2018)[94]	Adult	Evaluation; Follow-up	Outcome	Patient centered	Adverse events, readmission, and mortality following discharge; Continuity of patient care;	(B) Patient demographic and clinical characteristics; Feelings of anxiety or embarrassment; Physical and psychological effects of illness;	(None)	(F) Tools to facilitate discharge;	Triage model
Mion (2020)[95]	Adult	Follow-up	Process; Outcome	Safe; Patient centered	Patient and family needs and experiences during discharge; Discharge education for patients and families; Adverse events, readmission, and mortality following discharge; Continuity of patient care;	(F) Provider-patient communication; Discharge education; (B) Feelings of anxiety or embarrassment; Physical and psychological effects of illness;	(F) Provider-provider communication; Critical care transition program;	(None)	(None)
Moore (2013)[96]	Adult	Follow-up	Outcome	Safe	Adverse events, readmission, and mortality following discharge; Timeliness of discharge (time of day, delay);	(B) Patient demographic and clinical characteristics;	(None)	(F&B) Time of discharge;	(None)
Moreira (2017)[97]	Adult	Execution	Structure	Safe; Timely	Adverse events, readmission, and mortality following	(B) Patient demographic and	(None)	(F&B) Time of discharge;	(None)

Author (Year)	Study Population	Phase(s) of Care	Donabedian[a]	IoM[b]	Themes	Patient & Family Facilitators (F) & Barriers (B)	Provider Facilitators (F) & Barriers (B)	Organizational Facilitators (F) & Barriers (B)	Tool
					discharge; Timeliness of discharge (time of day, delay);	clinical characteristics;			
Morris (2016)[98]	Adult	Execution	Process	Timely	Adverse events, readmission, and mortality following discharge; Timeliness of discharge (time of day, delay);	(None)	(None)	(B) Limited ICU and ward resources; Delay in discharge;	(None)
Morton B. (2020)[99]	Adult	Follow-up	Outcome	Safe	Planning for discharge; Adverse events, readmission, and mortality following discharge;	(B) Patient demographic and clinical characteristics;	(None)	(F) Tools to facilitate discharge;	(None)
Murray (2020)[100]	Adult	Evaluation; Planning	Process; Outcome	Efficient; Safe	Planning for discharge; Evaluating patient readiness for discharge; Resource use during discharge;	(B) ICU or hospital length of stay;	(F) Provider-provider communication; Collaboration between ICU and ward; Written documentation;	(F) Tools to facilitate discharge;	Guideline/ checklist
Ng (2018)[101]	Adult	Follow-up	Outcome	Safe; Timely	Adverse events, readmission, and mortality following discharge; Timeliness of discharge (time of day, delay);	(B) Patient demographic and clinical characteristics;	(None)	(B) Admission location before ICU;	(None)
Niven (2014)[102]	Adult	Follow-up	Outcome	Safe	Adverse events, readmission, and mortality following discharge; Critical care transition program (nurse liaison, outreach team);	(None)	(F) Critical care transition program;	(None)	(None)
Ofoma (2014)[103]	Adult	Execution	Outcome	Efficient; Safe	Adverse events, readmission, and mortality following discharge; Resource use during discharge;	(B) Lack of provider-patient communication;	(None)	(B) Impact of current discharge practices;	(None)

**Author (Year)**	**Study Population**	**Phase(s) of Care**	**Donabedian[a]**	**IoM[b]**	**Themes**	**Patient & Family Facilitators (F) & Barriers (B)**	**Provider Facilitators (F) & Barriers (B)**	**Organizational Facilitators (F) & Barriers (B)**	**Tool**
Ofoma (2018)[104]	Adult	Execution	Process; Structure	Effective; Efficient	Evaluating patient readiness for discharge; Resource use during discharge; Continuity of patient care;	(B) Patient demographic and clinical characteristics; Lack of provider-patient communication;	(F) Clinical judgement or decision making; (F&B) (Lack of) Provider-provider communication;	(B) Limited ICU and ward resources; Impact of current discharge practices; Discharge location; Hospital characteristics;	(None)
Ofoma (2020)[105]	Adult	Execution; Follow-up	Process; Outcome	Efficient; Timely	Adverse events, readmission, and mortality following discharge; Timeliness of discharge (time of day, delay);	(None)	(None)	(B) Impact of current discharge practices; Costs of healthcare provided; Delay in discharge;	(None)
Oh (2015)[106]	Adult	Execution	Process	Patient centered	Patient and family needs and experiences during discharge; Anxiety associated with discharge;	(F) Family engagement/ support; (B) Feelings of anxiety or embarrassment;	(None)	(None)	(None)
Ostergaard (2019)[107]	Adult	Execution	Process	Effective	Availability of complete and accurate discharge information; Standardizing the discharge process; Resource use during discharge;	(None)	(B) Workload;	(F) Tools to facilitate discharge; (B) Limited ICU and ward resources;	(None)
Oud & Chan (2018)[108]	Adult	Execution	Structure	Safe	Standardizing the discharge process;	(F&B) Patient demographic and clinical characteristics; (B) Feelings of anxiety or embarrassment; Logistical barriers;	(None)	(B) Impact of current discharge practices; Discharge location;	(None)

Author (Year)	Study Population	Phase(s) of Care	Donabedian[a]	IoM[b]	Themes	Patient & Family Facilitators (F) & Barriers (B)	Provider Facilitators (F) & Barriers (B)	Organizational Facilitators (F) & Barriers (B)	Tool
								Hospital characteristics;	
Parenmark (2019)[109]	Adult	Execution	Process; Outcome	Safe	Adverse events, readmission, and mortality following discharge; Timeliness of discharge (time of day, delay);	(None)	(None)	(F&B) Time of discharge;	(None)
Patel (2020)[110]	Adult	Planning; Execution; Follow-up	Process; Outcome	Efficient; Safe; Patient centered	Planning for discharge; Adverse events, readmission, and mortality following discharge; Timeliness of discharge (time of day, delay);	(None)	(F) Provider-provider communication; Collaboration between ICU and ward; (B) Workload;	(F) Discharge location; (B) Delay in discharge;	(None)
Pattison (2015)[111]	Adult	Follow-up	Outcome	Effective; Patient centered	Anxiety associated with discharge;	(B) Feelings of anxiety or embarrassment; Physical and psychological effects of illness;	(None)	(None)	(None)
Peltonen (2015)[112]	Adult	Execution	Process; Structure	Efficient; Timely	Timeliness of discharge (time of day, delay); Resource use during discharge;	(None)	(B) Workload; Lack of provider-provider communication;	(B) Limited ICU and ward resources; Time of discharge; Impact of current discharge practices; Discharge location;	(None)
Peters (2017)[113]	Adult	Execution	Process; Structure	Effective; Efficient	Patient and family needs and experiences during discharge; Continuity of patient care; Medication reconciliation;	(F) Provider-patient communication;	(F) Provider-provider communication; Collaboration between ICU and ward; Written documentation;	(F) Guidelines or policies;	(None)

Author (Year)	Study Population	Phase(s) of Care	Donabedian[a]	IoM[b]	Themes	Patient & Family Facilitators (F) & Barriers (B)	Provider Facilitators (F) & Barriers (B)	Organizational Facilitators (F) & Barriers (B)	Tool
Powell (2020)[114]	Adult	Execution	Process	Effective; Safe	Availability of complete and accurate discharge information; Standardizing the discharge process; Resource use during discharge; Continuity of patient care;	(None)	(F) Provider-provider communication; Collaboration between ICU and ward; Knowledge/ experience of provider; Written documentation; (B) Workload;	(F) Guidelines or policies; Tools to facilitate discharge; (F&B) Impact of current discharge practices; (B) Delay in discharge; Hospital characteristics; Infrastructure;	Guideline/ checklist
Rai (2019)[115]	Adult	Follow-up	Outcome	Safe; Patient centered	Anxiety associated with discharge; Discharge education for providers;	(B) Patient demographic and clinical characteristics; Physical and psychological effects of illness;	(F) Collaboration between ICU and ward; Knowledge/ experience of provider;	(F) Education/ training of providers; (B) Limited ICU and ward resources; Discharge location;	(None)
Ramos (2014)[116]	Adult	Execution	Process; Outcome	Effective; Safe	Medication reconciliation;	(None)	(None)	(F) Use of best practices; (F&B) Impact of current discharge practices;	(None)
Ramsay (2013)[117]	Adult	Follow-up	Process	Patient centered	Patient and family needs and experiences during discharge; Availability of complete and accurate discharge information; Continuity of patient care; Autonomy;	(F) Provider-patient communication; Self-efficacy; (B) Feelings of anxiety or embarrassment; Expectations; Physical and psychological effects of illness;	(B) Workload;	(F) Follow-up clinic or program;	(None)
Ramsay (2016)[118]	Adult	Execution	Process	Effective	Patient and family needs and	(F) Provider-patient	(None)	(None)	(None)

Author (Year)	Study Population	Phase(s) of Care	Donabedian[a]	IoM[b]	Themes	Patient & Family Facilitators (F) & Barriers (B)	Provider Facilitators (F) & Barriers (B)	Organizational Facilitators (F) & Barriers (B)	Tool
					experiences during discharge; Continuity of patient care;	communication; Family engagement/ support; Written communication;			
Ranzani (2014)[119]	Adult	Follow-up	Outcome	Safe	Adverse events, readmission, and mortality following discharge; Evaluating patient readiness for discharge;	(F&B) Patient demographic and clinical characteristics;	(None)	(F) Discharge location; Admission location before ICU;	(None)
Rath (2020)[120]	Adult	Execution; Follow-up	Process; Outcome	Patient centered	Patient and family needs and experiences during discharge; Availability of complete and accurate discharge information; Discharge education for patients and families; Continuity of patient care; Medication reconciliation;	(F) Provider-patient communication; Discharge education; Family engagement/ support; (B) Patient demographic and clinical characteristics; Feelings of anxiety or embarrassment; Expectations; Physical and psychological effects of illness; ICU or hospital length of stay; Financial obstacles;	(None)	(F&B) Discharge location; (B) Time of discharge; Impact of current discharge practices; Costs of healthcare provided;	(None)
Reineck (2015)[121]	Adult	Follow-up	Outcome	Efficient; Safe	Adverse events, readmission, and mortality following discharge;	(None)	(None)	(F) Impact of current discharge practices; Hospital characteristics;	(None)
Rice (2020)[122]	Adult	Execution	Process; Outcome	Safe; Timely	Availability of complete and	(None)	(F&B) (Lack of) Provider-	(F) Education/ training of	(None)

Author (Year)	Study Population	Phase(s) of Care	Donabedian[a]	IoM[b]	Themes	Patient & Family Facilitators (F) & Barriers (B)	Provider Facilitators (F) & Barriers (B)	Organizational Facilitators (F) & Barriers (B)	Tool
					accurate discharge information; Standardizing the discharge process; Adverse events, readmission, and mortality following discharge; Discharge education for providers; Medication reconciliation;		provider communication;	providers; (B) Costs of healthcare provided;	
Rizvi (2019)[123]	Adult	Follow-up	Outcome	Safe; Patient centered	Medication reconciliation;	(None)	(F) Clinical judgement or decision making;	(None)	(None)
Rojas (2018)[124]	Adult	Evaluation; Follow-up	Outcome	Safe	Adverse events, readmission, and mortality following discharge;	(F&B) Patient demographic and clinical characteristics;	(None)	(B) Impact of current discharge practices;	(None)
Safavi (2019)[125]	Adult	Execution	Process	Timely	Availability of complete and accurate discharge information; Timeliness of discharge (time of day, delay);	(None)	(F) Clinical judgement or decision making;	(F&B) Impact of current discharge practices; (B) Delay in discharge;	(None)
Sanson (2020)[126]	Adult	Evaluation; Execution; Follow-up	Process; Outcome	Safe	Adverse events, readmission, and mortality following discharge; Evaluating patient readiness for discharge; Continuity of patient care;	(B) Patient demographic and clinical characteristics;	(F) Collaboration between ICU and ward; Written documentation;	(B) Limited ICU and ward resources; Impact of current discharge practices;	(None)
Santamaria (2015)[127]	Adult	Execution	Structure; Outcome	Effective; Safe; Timely	Planning for discharge; Adverse events, readmission, and mortality following discharge; Timeliness of	(B) Patient demographic and clinical characteristics;	(None)	(F&B) Time of discharge;	(None)

Author (Year)	Study Population	Phase(s) of Care	Donabedian[a]	IoM[b]	Themes	Patient & Family Facilitators (F) & Barriers (B)	Provider Facilitators (F) & Barriers (B)	Organizational Facilitators (F) & Barriers (B)	Tool
					discharge (time of day, delay);				
Santhosh (2019)[128]	Adult	Execution	Process; Outcome	Effective; Efficient; Safe	Patient and family needs and experiences during discharge; Availability of complete and accurate discharge information; Standardizing the discharge process; Adverse events, readmission, and mortality following discharge; Resource use during discharge; Continuity of patient care;	(F) Written communication;	(F) Collaboration between ICU and ward; Knowledge/ experience of provider; Written documentation; (F&B) (Lack of) Provider-provider communication; (B) Workload;	(F) Guidelines or policies; Education/ training of providers; (F&B) Impact of current discharge practices; (B) Limited ICU and ward resources; Staffing;	(None)
Sauro (2020)[129]	Adult	Execution; Follow-up	Process; Outcome	Safe	Adverse events, readmission, and mortality following discharge; Continuity of patient care;	(None)	(F) Provider-provider communication; (B) Lack of knowledge/ experience of provider;	(B) Impact of current discharge practices; Hospital or ICU capacity;	(None)
Sayde (2020)[130]	Adult	Execution; Follow-up	Process; Outcome	Effective; Patient centered	Patient and family needs and experiences during discharge; Availability of complete and accurate discharge information; Adverse events, readmission, and mortality following discharge; Anxiety associated with discharge;	(F) Provider-patient communication; Family engagement/ support; (B) Patient demographic and clinical characteristics; Feelings of anxiety or embarrassment; Physical and	(None)	(F) Education/ training of providers; Use of best practices;	(None)

Author (Year)	Study Population	Phase(s) of Care	Donabedian[a]	IoM[b]	Themes	Patient & Family Facilitators (F) & Barriers (B)	Provider Facilitators (F) & Barriers (B)	Organizational Facilitators (F) & Barriers (B)	Tool
						psychological effects of illness;			
Sevin (2018)[131]	Adult	Follow-up	Process; Structure; Outcome	Effective; Patient centered	Discharge education for patients and families; Adverse events, readmission, and mortality following discharge; Critical care transition program (nurse liaison, outreach team); Continuity of patient care;	(F) Discharge education; (F&B) Patient demographic and clinical characteristics; (B) Physical and psychological effects of illness;	(F) Critical care transition program;	(None)	(None)
Sevin & Jackson (2019)[132]	Adult	Follow-up	Structure	Effective; Efficient; Patient centered	Adverse events, readmission, and mortality following discharge; Continuity of patient care; Discharge education for providers;	(F) Provider-patient communication; (B) Patient demographic and clinical characteristics; Feelings of anxiety or embarrassment; Physical and psychological effects of illness;	(F) Knowledge/ experience of provider; (B) Lack of provider-provider communication;	(F) Follow-up clinic or program; (F&B) (Lack of) Education/ training of providers; (B) Costs of healthcare provided; Discharge location;	(None)
Shimogai (2019)[133]	Adult	Evaluation; Planning; Follow-up	Process; Outcome	Effective; Safe; Patient centered	Planning for discharge; Evaluating patient readiness for discharge; Autonomy;	(F&B) Patient demographic and clinical characteristics; (B) Physical and psychological effects of illness;	(None)	(F) Follow-up clinic or program;	(None)
Siddiqui (2013)[134]	Adult	Follow-up	Outcome	Safe	Adverse events, readmission, and mortality following discharge;	(B) Patient demographic and clinical characteristics;	(None)	(None)	(None)
Sirvent (2016)[135]	Adult	Planning; Execution	Process	Efficient; Timely	Standardizing the discharge process; Timeliness of	(None)	(None)	(F) Time of discharge; Discharge	(None)

**Author (Year)**	**Study Population**	**Phase(s) of Care**	**Donabedian[a]**	**IoM[b]**	**Themes**	**Patient & Family Facilitators (F) & Barriers (B)**	**Provider Facilitators (F) & Barriers (B)**	**Organizational Facilitators (F) & Barriers (B)**	**Tool**
					discharge (time of day, delay); Resource use during discharge;			location; Hospital characteristics;	
Smith (2018)[136]	Adult	Execution	Outcome	Patient centered	Patient and family needs and experiences during discharge; Continuity of patient care;	(F) Family engagement/ support; Expectations;	(None)	(F) Discharge location;	(None)
Stelfox (2016)[137]	Adult	Follow-up	Outcome	Efficient; Safe	Adverse events, readmission, and mortality following discharge; Critical care transition program (nurse liaison, outreach team);	(B) Patient demographic and clinical characteristics;	(F) Critical care transition program; Multidisciplinary team;	(B) Impact of current discharge practices;	(None)
Stelfox (2017)[138]	Adult	Planning; Execution	Process; Structure	Effective; Efficient; Timely	Patient and family needs and experiences during discharge; Availability of complete and accurate discharge information; Standardizing the discharge process; Timeliness of discharge (time of day, delay); Continuity of patient care;	(F) Provider-patient communication; Written communication; Patient/ family treated as a member of healthcare team;	(F) Provider-provider communication; Collaboration between ICU and ward; Written documentation;	(B) Impact of current discharge practices; Costs of healthcare provided; Delay in discharge;	(None)
Stelfox (2018)[139]	Adult	Evaluation; Follow-up	Outcome	Efficient; Safe; Timely	Adverse events, readmission, and mortality following discharge;	(F) Patient demographic and clinical characteristics;	(None)	(F&B) Admission location before ICU; Discharge location;	(None)
Szubski (2014)[140]	Adult	Evaluation	Process	Efficient; Patient centered	Planning for discharge;	(F&B) Patient demographic and clinical characteristics;	(F) Clinical judgement or decision making;	(F) Discharge location;	Prediction tool

Author (Year)	Study Population	Phase(s) of Care	Donabedian[a]	IoM[b]	Themes	Patient & Family Facilitators (F) & Barriers (B)	Provider Facilitators (F) & Barriers (B)	Organizational Facilitators (F) & Barriers (B)	Tool
Taniguchi (2019)[141]	Adult	Evaluation; Follow-up	Process; Outcome	Effective; Safe	Adverse events, readmission, and mortality following discharge; Evaluating patient readiness for discharge;	(B) Patient demographic and clinical characteristics;	(F) Clinical judgement or decision making;	(F) Education/ training of providers; Tools to facilitate discharge; (B) Impact of current discharge practices;	(None)
Tully (2019)[142]	Adult	Execution	Process; Outcome	Safe; Patient centered	Availability of complete and accurate discharge information; Adverse events, readmission, and mortality following discharge; Medication reconciliation;	(None)	(F) Provider-provider communication;	(F) Use of best practices;	(None)
Uppanisakorn (2018)[143]	Adult	Follow-up	Outcome	Safe	Adverse events, readmission, and mortality following discharge;	(B) Patient demographic and clinical characteristics;	(None)	(None)	(None)
van Mol (2017)[144]	Adult	Planning; Execution	Process	Effective; Safe; Patient centered	Patient and family needs and experiences during discharge; Discharge education for patients and families; Planning for discharge; Standardizing the discharge process; Anxiety associated with discharge; Critical care transition program (nurse liaison, outreach team);	(F) Provider-patient communication; Discharge education; Family engagement/ support; Written communication; (B) Feelings of anxiety or embarrassment; Physical and psychological effects of illness;	(F) Provider-provider communication; Critical care transition program; Collaboration between ICU and ward; (B) Workload;	(B) Limited ICU and ward resources;	(None)

Author (Year)	Study Population	Phase(s) of Care	Donabedian[a]	IoM[b]	Themes	Patient & Family Facilitators (F) & Barriers (B)	Provider Facilitators (F) & Barriers (B)	Organizational Facilitators (F) & Barriers (B)	Tool
van Mol (2018)[145]	Adult	Follow-up	Structure; Outcome	Safe; Patient centered	Patient and family needs and experiences during discharge; Critical care transition program (nurse liaison, outreach team); Discharge education for providers;	(F) Provider-patient communication; Discharge education;	(F) Critical care transition program;	(F) Guidelines or policies;	(None)
van Sluisveld (2017)[146]	Adult	Execution; Follow-up	Process; Outcome	Safe	Standardizing the discharge process; Adverse events, readmission, and mortality following discharge;	(None)	(F) Provider-provider communication;	(F) Use of best practices;	(None)
Vollam (2018)[147]	Adult	Execution; Follow-up	Structure; Outcome	Safe	Adverse events, readmission, and mortality following discharge; Timeliness of discharge (time of day, delay);	(None)	(None)	(F&B) Time of discharge;	(None)
Vollam (2020)[148]	Adult	Follow-up	Outcome	Effective; Safe	Adverse events, readmission, and mortality following discharge;	(None)	(None)	(F) Use of best practices; Discharge location;	(None)
Wagner (2013)[149]	Adult	Execution; Follow-up	Structure; Outcome	Safe; Timely	Adverse events, readmission, and mortality following discharge; Timeliness of discharge (time of day, delay); Resource use during discharge;	(F&B) Patient demographic and clinical characteristics;	(None)	(F&B) Impact of current discharge practices; Admission location before ICU; (B) Limited ICU and ward resources; Hospital or ICU capacity;	(None)
White (2019)[150]	Adult	Planning; Execution	Process; Structure	Safe; Patient centered	Patient and family needs and experiences during	(F) Provider-patient communication;	(F) Knowledge/ experience of provider; Clinical	(F) Impact of current	(None)

Author (Year)	Study Population	Phase(s) of Care	Donabedian[a]	IoM[b]	Themes	Patient & Family Facilitators (F) & Barriers (B)	Provider Facilitators (F) & Barriers (B)	Organizational Facilitators (F) & Barriers (B)	Tool
					discharge; Planning for discharge;	(F&B) Expectations;	judgement or decision making; Multidisciplinary team;	discharge practices;	
Won and Son (2020) [151]	Adult	Follow-up	Outcome	Patient centered	Patient and family needs and experiences during discharge; Anxiety associated with discharge;	(F) Expectations; (B) Feelings of anxiety or embarrassment;	(None)	(None)	(None)
Wood (2014)[152]	Adult	Execution	Structure	Timely	Timeliness of discharge (time of day, delay);	(None)	(None)	(F) Time of discharge;	(None)
Xing (2019)[153]	Adult	Follow-up	Process; Outcome	Efficient; Safe	Adverse events, readmission, and mortality following discharge; Timeliness of discharge (time of day, delay);	(F&B) Patient demographic and clinical characteristics;	(None)	(F) Discharge location;	(None)
Yang (2016)[154]	Adult	Execution; Follow-up	Outcome	Safe; Timely	Adverse events, readmission, and mortality following discharge; Timeliness of discharge (time of day, delay);	(None)	(None)	(B) Time of discharge; Delay in discharge;	(None)
Yun (2017)[155]	Adult	Evaluation; Planning; Execution	Process; Structure	Effective	Patient and family needs and experiences during discharge; Planning for discharge; Critical care transition program (nurse liaison, outreach team); Evaluating patient readiness for discharge;	(F) Patient demographic and clinical characteristics; Family engagement/ support; (B) Feelings of anxiety or embarrassment;	(F) Critical care transition program;	(None)	Guideline/ checklist
Zaidi (2019)[156]	Adult	Evaluation; Follow-up	Outcome	Safe	Standardizing the discharge process; Evaluating patient	(F) Patient demographic and	(None)	(F) Discharge location;	(None)

Author (Year)	Study Population	Phase(s) of Care	Donabedian[a]	IoM[b]	Themes	Patient & Family Facilitators (F) & Barriers (B)	Provider Facilitators (F) & Barriers (B)	Organizational Facilitators (F) & Barriers (B)	Tool
					readiness for discharge;	clinical characteristics;			
Zilahi & O'Connor (2019)[157]	Adult	Execution; Follow-up	Process	Effective; Patient centered	Continuity of patient care;	(B) Feelings of anxiety or embarrassment;	(F) Written documentation; Clear roles and responsibilities; Multidisciplinary team; (F&B) (Lack of) Provider-provider communication;	(F) Hospital characteristics; (B) Impact of current discharge practices;	(None)
Tiruvoipati (2017)[158]	Adult; Pediatric	Execution	Structure	Timely	Timeliness of discharge (time of day, delay);	(B) Patient demographic and clinical characteristics;	(None)	(B) Time of discharge; Impact of current discharge practices;	(None)
van Sluisveld (2015)[159]	Adult; Pediatric	Planning; Execution; Follow-up	Process; Outcome	Effective; Efficient; Safe; Timely	Availability of complete and accurate discharge information; Planning for discharge; Standardizing the discharge process; Adverse events, readmission, and mortality following discharge; Critical care transition program (nurse liaison, outreach team); Continuity of patient care;	(None)	(F) Critical care transition program; Written documentation; (B) Lack of provider-provider communication;	(F) Tools to facilitate discharge;	(None)
van Sluisveld (2017)[160]	Adult; Pediatric	Planning; Execution	Process; Structure	Effective; Efficient; Patient centered	Patient and family needs and experiences during discharge; Availability of complete and	(F) Provider-patient communication;	(F) Provider-provider communication; Collaboration between ICU and	(F) Guidelines or policies; Tools to facilitate discharge; (B)	(None)

Author (Year)	Study Population	Phase(s) of Care	Donabedian[a]	IoM[b]	Themes	Patient & Family Facilitators (F) & Barriers (B)	Provider Facilitators (F) & Barriers (B)	Organizational Facilitators (F) & Barriers (B)	Tool
					accurate discharge information; Planning for discharge; Standardizing the discharge process; Resource use during discharge; Medication reconciliation;		ward; Written documentation; (B) Workload; Lack of knowledge/ experience of provider;	Limited ICU and ward resources; Impact of current discharge practices; Costs of healthcare provided; Hospital characteristics; Infrastructure; Lack of education/ training of providers;	
Herbst (2018)[161]	Adult; Pediatric; Neonatal	Planning; Execution; Follow-up	Process; Structure	Effective; Efficient; Safe; Patient centered ; Timely	Patient and family needs and experiences during discharge; Discharge education for patients and families; Standardizing the discharge process; Adverse events, readmission, and mortality following discharge; Timeliness of discharge (time of day, delay); Critical care transition program (nurse liaison, outreach team); Evaluating patient readiness for discharge; Resource use during discharge; Continuity of patient care; Medication reconciliation;	(F) Patient demographic and clinical characteristics; Provider-patient communication; Discharge education; Family engagement/ support; Written communication;	(F) Provider-provider communication; Critical care transition program; Collaboration between ICU and ward; Knowledge/ experience of provider; Clinical judgement or decision making;	(F) Guidelines or policies; Use of best practices; Tools to facilitate discharge; (B) Delay in discharge; Discharge location;	(None)

Author (Year)	Study Population	Phase(s) of Care	Donabedian[a]	IoM[b]	Themes	Patient & Family Facilitators (F) & Barriers (B)	Provider Facilitators (F) & Barriers (B)	Organizational Facilitators (F) & Barriers (B)	Tool
Li (2015)[162]	Adult; Pediatric; Neonatal	Execution	Process; Structure	Effective; Efficient	Availability of complete and accurate discharge information; Timeliness of discharge (time of day, delay); Continuity of patient care;	(F) Provider-patient communication; Family engagement/ support; Written communication;	(F) Provider-provider communication; Collaboration between ICU and ward;	(None)	(None)
Alali (2019)[163]	Pediatric	Planning; Execution; Follow-up	Process; Structure; Outcome	Efficient; Safe; Timely	Planning for discharge; Standardizing the discharge process; Timeliness of discharge (time of day, delay); Evaluating patient readiness for discharge;	(None)	(F) Provider-provider communication; Collaboration between ICU and ward; Written documentation;	(F) Guidelines or policies; Education/ training of providers; Tools to facilitate discharge; (F&B) Impact of current discharge practices; (B) Delay in discharge;	(None)
Baker (2016)[164]	Pediatric	Evaluation; Planning; Execution	Process	Efficient; Patient centered	Discharge education for patients and families; Standardizing the discharge process; Evaluating patient readiness for discharge; Discharge education for providers;	(F) Discharge education; (B) Patient demographic and clinical characteristics;	(None)	(F) Guidelines or policies; Use of best practices; Tools to facilitate discharge;	Educational tool
Bedford & Bench (2018)[165]	Pediatric	Execution	Process; Outcome	Effective; Patient centered	Patient and family needs and experiences during discharge; Discharge education for patients and families; Adverse events, readmission, and mortality following discharge;	(F) Provider-patient communication; Discharge education; Family engagement/ support; (B) Patient demographic and	(None)	(None)	(None)

Author (Year)	Study Population	Phase(s) of Care	Donabedian[a]	IoM[b]	Themes	Patient & Family Facilitators (F) & Barriers (B)	Provider Facilitators (F) & Barriers (B)	Organizational Facilitators (F) & Barriers (B)	Tool
					Anxiety associated with discharge; Continuity of patient care;	clinical characteristics; Feelings of anxiety or embarrassment; Physical and psychological effects of illness;			
Berube (2014)[166]	Pediatric	Execution	Process; Structure	Patient centered	Patient and family needs and experiences during discharge; Discharge education for patients and families; Anxiety associated with discharge; Evaluating patient readiness for discharge;	(F) Provider-patient communication; Discharge education; Family engagement/ support; (B) Feelings of anxiety or embarrassment;	(None)	(None)	(None)
Crowe (2020)[167]	Pediatric	Execution; Follow-up	Structure; Outcome	Safe; Patient centered	Patient and family needs and experiences during discharge; Anxiety associated with discharge; Autonomy;	(F) Discharge education; Family engagement/ support; Patient/ family treated as a member of healthcare team; (B) Feelings of anxiety or embarrassment; Expectations; Logistical barriers;	(None)	(B) Impact of current discharge practices;	(None)
De la Oliva (2018)[168]	Pediatric	Evaluation; Execution	Structure	Efficient; Safe; Timely	Planning for discharge; Standardizing the discharge process; Timeliness of discharge (time of day, delay); Evaluating patient readiness for discharge; Resource use during discharge;	(F&B) Patient demographic and clinical characteristics;	(F) Provider-provider communication;	(F) Guidelines or policies; (B) Delay in discharge;	Guideline/ checklist

Author (Year)	Study Population	Phase(s) of Care	Donabedian[a]	IoM[b]	Themes	Patient & Family Facilitators (F) & Barriers (B)	Provider Facilitators (F) & Barriers (B)	Organizational Facilitators (F) & Barriers (B)	Tool
Ertugrul (2017)[169]	Pediatric	Execution; Follow-up	Structure; Outcome	Safe	Adverse events, readmission, and mortality following discharge;	(None)	(F) Clinical judgement or decision making;	(F) Impact of current discharge practices; Discharge location;	(None)
Foster (2019)[170]	Pediatric	Follow-up	Process	Patient centered	Patient and family needs and experiences during discharge; Anxiety associated with discharge; Continuity of patient care;	(F) Family engagement/ support;	(F) Critical care transition program;	(F) Tools to facilitate discharge;	(None)
Frankel (2019)[171]	Pediatric	Evaluation; Follow-up	Structure	Effective; Efficient; Safe	Availability of complete and accurate discharge information; Planning for discharge; Standardizing the discharge process; Resource use during discharge;	(F) Provider-patient communication; Family engagement/ support;	(F) Provider-provider communication; Collaboration between ICU and ward; Written documentation;	(F) Guidelines or policies; Education/ training of providers; Use of best practices; Impact of current discharge practices; Follow-up clinic or program;	(None)
Garcia (2020)[172]	Pediatric	Planning	Outcome	Patient centered	Patient and family needs and experiences during discharge; Planning for discharge; Evaluating patient readiness for discharge;	(F) Provider-patient communication;	(F) Provider-provider communication; Critical care transition program; Multidisciplinary team;	(F) Guidelines or policies; Use of best practices; Discharge location;	(None)
Goldstein (2019)[173]	Pediatric	Planning; Execution; Follow-up	Process	Effective; Safe; Patient centered	Patient and family needs and experiences during discharge; Discharge education for patients	(F) Discharge education; Expectations;	(F) Knowledge/ experience of provider;	(None)	(None)

Author (Year)	Study Population	Phase(s) of Care	Donabedian[a]	IoM[b]	Themes	Patient & Family Facilitators (F) & Barriers (B)	Provider Facilitators (F) & Barriers (B)	Organizational Facilitators (F) & Barriers (B)	Tool
					and families; Planning for discharge;				
Graham (2019)[174]	Pediatric	Execution	Structure	Effective; Patient centered	Patient and family needs and experiences during discharge; Availability of complete and accurate discharge information; Discharge education for patients and families; Anxiety associated with discharge; Critical care transition program (nurse liaison, outreach team); Discharge education for providers;	(F) Provider-patient communication; Discharge education; Family engagement/ support; (B) Feelings of anxiety or embarrassment;	(F) Critical care transition program;	(F) Discharge location;	(None)
Kennedy & Numa (2020)[175]	Pediatric	Evaluation; Execution	Process	Efficient; Safe; Timely	Timeliness of discharge (time of day, delay); Evaluating patient readiness for discharge;	(None)	(None)	(F&B) Time of discharge; Discharge location; (B) Limited ICU and ward resources; Delay in discharge; Admission location before ICU;	(None)
Kroeger (2017)[176]	Pediatric	Planning; Follow-up	Outcome	Effective; Safe	Planning for discharge; Adverse events, readmission, and mortality following discharge; Evaluating patient readiness for discharge;	(B) Patient demographic and clinical characteristics; ICU or hospital length of stay;	(None)	(B) Hospital characteristics; Hospital or ICU capacity;	(None)

Author (Year)	Study Population	Phase(s) of Care	Donabedian[a]	IoM[b]	Themes	Patient & Family Facilitators (F) & Barriers (B)	Provider Facilitators (F) & Barriers (B)	Organizational Facilitators (F) & Barriers (B)	Tool
Kulesa (2020)[177]	Pediatric	Evaluation; Planning; Execution	Process	Efficient; Patient centered	Planning for discharge; Standardizing the discharge process; Evaluating patient readiness for discharge; Resource use during discharge; Continuity of patient care;	(None)	(F) Provider-provider communication; Collaboration between ICU and ward; Written documentation; Clinical judgement or decision making;	(F) Tools to facilitate discharge; (B) Impact of current discharge practices;	Guideline/ checklist
Leyenaar (2016)[178]	Pediatric	Follow-up	Outcome	Effective; Safe	Adverse events, readmission, and mortality following discharge; Timeliness of discharge (time of day, delay); Resource use during discharge;	(None)	(F) Provider-provider communication; Collaboration between ICU and ward; Written documentation;	(F&B) Impact of current discharge practices; (B) Delay in discharge;	(None)
Lobos (2015)[179]	Pediatric	Execution; Follow-up	Process; Outcome	Effective; Efficient	Critical care transition program (nurse liaison, outreach team); Continuity of patient care;	(F) Patient demographic and clinical characteristics;	(F) Critical care transition program;	(F) Impact of current discharge practices;	(None)
Manente (2017)[180]	Pediatric	Planning	Structure	Patient centered	Patient and family needs and experiences during discharge; Discharge education for patients and families; Planning for discharge;	(F) Discharge education; Written communication; (B) Feelings of anxiety or embarrassment;	(None)	(F) Tools to facilitate discharge;	Transfer brochure
Moore (2015)[181]	Pediatric	Execution	Process	Effective; Patient centered	Patient and family needs and experiences during discharge; Discharge education for patients and families; Continuity of patient care; Autonomy;	(F) Provider-patient communication; Discharge education;	(F) Provider-provider communication;	(F) Use of best practices;	(None)

Author (Year)	Study Population	Phase(s) of Care	Donabedian[a]	IoM[b]	Themes	Patient & Family Facilitators (F) & Barriers (B)	Provider Facilitators (F) & Barriers (B)	Organizational Facilitators (F) & Barriers (B)	Tool
Neupane (2015)[182]	Pediatric	Planning; Follow-up	Process; Structure	Effective; Patient centered	Planning for discharge; Adverse events, readmission, and mortality following discharge; Evaluating patient readiness for discharge; Continuity of patient care;	(F) Patient demographic and clinical characteristics; Provider-patient communication; Family engagement/ support;	(F) Provider-provider communication; Critical care transition program; Clear roles and responsibilities;	(F) Discharge location;	Guideline/ checklist
Noje (2015)[183]	Pediatric	Execution	Process; Structure	Effective; Safe; Patient centered	Patient and family needs and experiences during discharge;	(F) Family engagement/ support;	(F) Empathy;	(F&B) Discharge location;	(None)
Obas (2016)[184]	Pediatric	Execution	Process	Patient centered	Patient and family needs and experiences during discharge; Discharge education for patients and families; Planning for discharge; Continuity of patient care;	(F) Provider-patient communication; Discharge education; (F&B) Expectations; (B) Feelings of anxiety or embarrassment;	(F) Collaboration between ICU and ward;	(None)	(None)
Orenstein (2019)[185]	Pediatric	Planning; Execution	Structure	Effective; Safe; Patient centered	Availability of complete and accurate discharge information; Planning for discharge; Standardizing the discharge process; Timeliness of discharge (time of day, delay); Evaluating patient readiness for discharge; Resource use during discharge; Discharge education for providers;	(None)	(F) Provider-provider communication; Knowledge/ experience of provider; Written documentation;	(F) Education/ training of providers; Tools to facilitate discharge; (B) Impact of current discharge practices;	(None)
Sheth (2016)[186]	Pediatric	Execution	Process; Structure	Efficient; Safe	Standardizing the discharge process;	(None)	(F) Provider-provider	(F) Guidelines or policies;	Transfer tool

Author (Year)	Study Population	Phase(s) of Care	Donabedian[a]	IoM[b]	Themes	Patient & Family Facilitators (F) & Barriers (B)	Provider Facilitators (F) & Barriers (B)	Organizational Facilitators (F) & Barriers (B)	Tool
							communication; Collaboration between ICU and ward;	Education/ training of providers; (B) Impact of current discharge practices;	
Smith (2018)[187]	Pediatric	Follow-up	Outcome	Safe	Adverse events, readmission, and mortality following discharge;	(F&B) Patient demographic and clinical characteristics;	(None)	(None)	(None)
Sobotka (2020)[188]	Pediatric	Execution	Structure	Effective; Safe	Continuity of patient care; Discharge education for providers;	(None)	(F) Provider-provider communication; (F&B) (Lack of) Knowledge/ experience of provider;	(F) Education/ training of providers; (B) Impact of current discharge practices;	(None)
Vollmer (2013)[189]	Pediatric	Planning; Execution	Process	Efficient; Timely	Availability of complete and accurate discharge information; Standardizing the discharge process; Continuity of patient care;	(None)	(F) Written documentation; (F&B) (Lack of) Provider-provider communication;	(F) Guidelines or policies;	(None)
Warrick (2015)[190]	Pediatric	Execution	Process	Effective; Safe	Standardizing the discharge process; Discharge education for providers;	(None)	(F) Provider-provider communication; Written documentation; Clear roles and responsibilities;	(F) Education/ training of providers; Impact of current discharge practices;	(None)
Barone (2020)[191]	Pediatric; Neonatal	Follow-up	Process; Outcome	Patient centered	Patient and family needs and experiences during discharge; Anxiety associated with	(F) Provider-patient communication; Discharge education; Family engagement/	(None)	(F) Education/ training of providers; Follow-up clinic or program;	(None)

**Author (Year)**	**Study Population**	**Phase(s) of Care**	**Donabedian[a]**	**IoM[b]**	**Themes**	**Patient & Family Facilitators (F) & Barriers (B)**	**Provider Facilitators (F) & Barriers (B)**	**Organizational Facilitators (F) & Barriers (B)**	**Tool**
					discharge; Continuity of patient care;	support; (B) Patient demographic and clinical characteristics; Feelings of anxiety or embarrassment;			
Morton K. (2019)[192]	Pediatric; Neonatal	Planning; Execution	Process; Structure	Patient centered	Patient and family needs and experiences during discharge; Autonomy;	(B) Expectations; Lack of provider-patient communication;	(B) Provider anxiety;	(F) Follow-up clinic or program; (B) Discharge location;	(None)
Amirani (2018)[193]	Neonatal	Planning	Process	Patient centered	Discharge education for patients and families; Planning for discharge;	(F) Discharge education; Self-efficacy;	(None)	(None)	(None)
Aydon (2017)[194]	Neonatal	Planning; Execution	Process	Patient centered	Patient and family needs and experiences during discharge; Availability of complete and accurate discharge information; Discharge education for patients and families; Planning for discharge; Anxiety associated with discharge; Autonomy;	(F) Provider-patient communication; Discharge education; Family engagement/ support; Patient/ family treated as a member of healthcare team; Self-efficacy; (B) Feelings of anxiety or embarrassment;	(None)	(None)	(None)
Bapat (2016)[195]	Neonatal	Execution; Follow-up	Process; Outcome	Effective; Safe; Patient centered	Patient and family needs and experiences during discharge; Discharge education for patients and families; Planning for discharge; Adverse events, readmission, and mortality following discharge;	(F) Discharge education; (F&B) Patient demographic and clinical characteristics; (B) ICU or hospital length of stay; Financial obstacles;	(None)	(None)	Guideline/ checklist

**Author (Year)**	**Study Population**	**Phase(s) of Care**	**Donabedian[a]**	**IoM[b]**	**Themes**	**Patient & Family Facilitators (F) & Barriers (B)**	**Provider Facilitators (F) & Barriers (B)**	**Organizational Facilitators (F) & Barriers (B)**	**Tool**
Barkemeyer (2015)[196]	Neonatal	Evaluation; Planning; Execution; Follow-up	Process; Outcome	Safe	Planning for discharge; Evaluating patient readiness for discharge; Continuity of patient care;	(F) Provider-patient communication; (B) Patient demographic and clinical characteristics;	(None)	(F) Use of best practices;	(None)
Bathie (2013)[197]	Neonatal	Execution	Process	Effective; Patient centered	Critical care transition program (nurse liaison, outreach team); Evaluating patient readiness for discharge; Continuity of patient care;	(F) Discharge education; Family engagement/ support;	(F) Critical care transition program;	(None)	Guideline/ checklist
Berman (2019)[198]	Neonatal	Evaluation; Planning; Follow-up	Process; Outcome	Effective; Safe; Patient centered	Patient and family needs and experiences during discharge; Discharge education for patients and families; Planning for discharge; Anxiety associated with discharge; Evaluating patient readiness for discharge; Continuity of patient care;	(F) Provider-patient communication; Discharge education; Family engagement/ support; (F&B) Expectations; (B) Feelings of anxiety or embarrassment;	(F) Provider-provider communication;	(None)	(None)
Bos (2018)[199]	Neonatal	Planning; Follow-up	Process; Outcome	Effective; Patient centered	Patient and family needs and experiences during discharge; Discharge education for patients and families; Planning for discharge; Anxiety associated with discharge;	(F) Discharge education; Family engagement/ support;	(None)	(None)	(None)
Boss & Hobbs (2013)[200]	Neonatal	Execution; Follow-up	Process; Outcome	Effective; Safe; Patient centered	Adverse events, readmission, and mortality following	(F) Patient demographic and clinical characteristics;	(None)	(B) Costs of healthcare provided;	(None)

Author (Year)	Study Population	Phase(s) of Care	Donabedian[a]	IoM[b]	Themes	Patient & Family Facilitators (F) & Barriers (B)	Provider Facilitators (F) & Barriers (B)	Organizational Facilitators (F) & Barriers (B)	Tool
					discharge; Continuity of patient care;	Family engagement/ support;			
Bowles (2016)[201]	Neonatal	Evaluation; Planning	Process	Efficient; Safe; Patient centered	Discharge education for patients and families; Planning for discharge; Continuity of patient care;	(F) Provider-patient communication; Discharge education; Family engagement/ support;	(None)	(F) Tools to facilitate discharge;	Guideline/ checklist
Boykova (2016)[202]	Neonatal	Planning; Follow-up	Outcome	Patient centered	Patient and family needs and experiences during discharge; Discharge education for patients and families;	(F) Discharge education; (B) Expectations;	(None)	(B) Costs of healthcare provided;	(None)
Brodsgaard (2015)[203]	Neonatal	Planning; Execution; Follow-up	Process; Structure	Effective; Patient centered	Patient and family needs and experiences during discharge; Discharge education for patients and families; Planning for discharge; Standardizing the discharge process; Anxiety associated with discharge;	(F) Family engagement/ support; Written communication; Expectations;	(F) Provider-provider communication; Clear roles and responsibilities;	(F) Tools to facilitate discharge;	Guideline/ checklist
Buck (2020)[204]	Neonatal	Evaluation	Process	Patient centered	Patient and family needs and experiences during discharge; Evaluating patient readiness for discharge;	(F) Discharge education; Family engagement/ support; (F&B) Patient demographic and clinical characteristics; (B) Feelings of anxiety or embarrassment; Expectations;	(None)	(F) Tools to facilitate discharge;	Discharge assessment

**Author (Year)**	**Study Population**	**Phase(s) of Care**	**Donabedian[a]**	**IoM[b]**	**Themes**	**Patient & Family Facilitators (F) & Barriers (B)**	**Provider Facilitators (F) & Barriers (B)**	**Organizational Facilitators (F) & Barriers (B)**	**Tool**
Burgoa-Larrañga (2015)[205]	Neonatal	Execution; Follow-up	Process; Outcome	Effective; Safe	Adverse events, readmission, and mortality following discharge;	(F) Provider-patient communication;	(None)	(F) Impact of current discharge practices;	(None)
Burnham (2013)[206]	Neonatal	Planning	Process	Patient centered	Patient and family needs and experiences during discharge; Discharge education for patients and families; Anxiety associated with discharge;	(F) Discharge education; (B) Feelings of anxiety or embarrassment;	(None)	(None)	(None)
Carlos (2015)[207]	Neonatal	Execution	Process	Patient centered	Standardizing the discharge process; Continuity of patient care;	(F&B) Patient demographic and clinical characteristics;	(None)	(F) Hospital characteristics;	(None)
Carty (2018)[208]	Neonatal	Planning	Structure	Patient centered	Patient and family needs and experiences during discharge; Discharge education for patients and families;	(F) Provider-patient communication; Discharge education;	(None)	(None)	Peer support program
Chen (2016)[209]	Neonatal	Planning	Structure	Patient centered	Discharge education for patients and families;	(F) Discharge education;	(None)	(None)	Educational tool
Coughlin (2020)[210]	Neonatal	Execution	Process	Effective	Standardizing the discharge process;	(None)	(None)	(F) Use of best practices; Impact of current discharge practices;	(None)
Cresi (2020)[211]	Neonatal	Evaluation; Execution	Process; Outcome	Effective; Safe	Adverse events, readmission, and mortality following discharge; Evaluating patient readiness for discharge;	(F) Patient demographic and clinical characteristics;	(None)	(F) Tools to facilitate discharge;	Discharge assessment

Author (Year)	Study Population	Phase(s) of Care	Donabedian[a]	IoM[b]	Themes	Patient & Family Facilitators (F) & Barriers (B)	Provider Facilitators (F) & Barriers (B)	Organizational Facilitators (F) & Barriers (B)	Tool
Daicampi (2019)[212]	Neonatal	Evaluation; Execution	Process	Effective; Safe	Evaluating patient readiness for discharge;	(F&B) Patient demographic and clinical characteristics; (B) ICU or hospital length of stay;	(None)	(F) Use of best practices; Time of discharge; Impact of current discharge practices; Tools to facilitate discharge; (B) Delay in discharge;	(None)
Driscoll (2014)[213]	Neonatal	Execution	Structure	Efficient; Timely	Availability of complete and accurate discharge information;	(None)	(F) Provider-provider communication; Written documentation;	(None)	(None)
Enlow (2014)[214]	Neonatal	Planning; Execution; Follow-up	Process; Outcome	Patient centered	Patient and family needs and experiences during discharge;	(F) Provider-patient communication; (B) Patient demographic and clinical characteristics;	(None)	(None)	(None)
Enlow (2017)[215]	Neonatal	Execution; Follow-up	Process	Patient centered ; Equal	Patient and family needs and experiences during discharge; Anxiety associated with discharge;	(F) Family engagement/ support; (B) Feelings of anxiety or embarrassment; Expectations;	(None)	(F) Education/ training of providers;	(None)
Enlow (2019)[216]	Neonatal	Planning	Process	Patient centered	Discharge education for patients and families;	(F) Discharge education; (B) Feelings of anxiety or embarrassment;	(None)	(None)	(None)
Ermarth (2019)[217]	Neonatal	Follow-up	Outcome	Effective; Safe	Adverse events, readmission, and mortality following discharge; Evaluating patient readiness for discharge;	(F) Patient demographic and clinical characteristics;	(None)	(F) Discharge location;	(None)

Author (Year)	Study Population	Phase(s) of Care	Donabedian[a]	IoM[b]	Themes	Patient & Family Facilitators (F) & Barriers (B)	Provider Facilitators (F) & Barriers (B)	Organizational Facilitators (F) & Barriers (B)	Tool
Feehan (2020)[218]	Neonatal	Execution	Process	Effective	Patient and family needs and experiences during discharge; Critical care transition program (nurse liaison, outreach team); Continuity of patient care;	(F) Discharge education; Family engagement/ support;	(F) Critical care transition program; Multidisciplinary team;	(F) Tools to facilitate discharge;	Transfer tool
Fleming (2017)[219]	Neonatal	Planning	Process	Efficient; Patient centered	Planning for discharge; Evaluating patient readiness for discharge;	(None)	(None)	(F) Guidelines or policies;	Guideline/ checklist
Gad (2017)[220]	Neonatal	Follow-up	Process	Efficient; Patient centered ; Timely	Availability of complete and accurate discharge information; Standardizing the discharge process; Discharge education for providers;	(F) Patient demographic and clinical characteristics; Family engagement/ support;	(F) Provider-provider communication; Knowledge/ experience of provider; Written documentation; Clinical judgement or decision making;	(F) Tools to facilitate discharge;	(None)
Garfield (2014)[221]	Neonatal	Planning; Execution; Follow-up	Process; Outcome	Safe	Patient and family needs and experiences during discharge; Planning for discharge; Anxiety associated with discharge; Autonomy;	(F) Provider-patient communication; Coping mechanisms; (B) Patient demographic and clinical characteristics; Feelings of anxiety or embarrassment; Expectations;	(F) Provider-provider communication;	(None)	(None)
Giúdici (2018)[222]	Neonatal	Evaluation; Planning; Execution	Process	Safe; Patient centered	Patient and family needs and experiences during	(F) Discharge education; (B)	(F) Provider-provider communication;	(F) Guidelines or policies;	Guideline/ checklist

Author (Year)	Study Population	Phase(s) of Care	Donabedian[a]	IoM[b]	Themes	Patient & Family Facilitators (F) & Barriers (B)	Provider Facilitators (F) & Barriers (B)	Organizational Facilitators (F) & Barriers (B)	Tool
					discharge; Planning for discharge; Evaluating patient readiness for discharge; Continuity of patient care;	Feelings of anxiety or embarrassment;	Written documentation;		
Goldin (2020)[223]	Neonatal	Evaluation	Structure	Effective; Efficient	Availability of complete and accurate discharge information; Standardizing the discharge process; Timeliness of discharge (time of day, delay); Evaluating patient readiness for discharge; Resource use during discharge;	(None)	(F) Knowledge/ experience of provider; Clinical judgement or decision making;	(F) Guidelines or policies; Education/ training of providers; Use of best practices; (B) Costs of healthcare provided;	(None)
Granero-Molina (2019)[224]	Neonatal	Execution; Follow-up	Process; Outcome	Patient centered	Patient and family needs and experiences during discharge; Anxiety associated with discharge;	(F) Expectations; (B) Patient demographic and clinical characteristics; Feelings of anxiety or embarrassment; Logistical barriers;	(F) Critical care transition program;	(None)	(None)
Gupta (2019)[225]	Neonatal	Evaluation; Planning	Process; Structure	Effective; Patient centered	Patient and family needs and experiences during discharge; Planning for discharge; Anxiety associated with discharge; Evaluating patient readiness for discharge;	(F) Provider-patient communication; Discharge education; Family engagement/ support; (B) Feelings of anxiety or embarrassment;	(None)	(None)	(None)
Hobbs (2017)[226]	Neonatal	Follow-up	Outcome	Effective; Safe; Patient centered	Patient and family needs and experiences during discharge; Availability	(F) Provider-patient communication; Written	(None)	(F) Education/ training of providers;	(None)

**Author (Year)**	**Study Population**	**Phase(s) of Care**	**Donabedian[a]**	**IoM[b]**	**Themes**	**Patient & Family Facilitators (F) & Barriers (B)**	**Provider Facilitators (F) & Barriers (B)**	**Organizational Facilitators (F) & Barriers (B)**	**Tool**
					of complete and accurate discharge information; Discharge education for patients and families; Anxiety associated with discharge; Continuity of patient care;	communication; Coping mechanisms; (B) Feelings of anxiety or embarrassment; Expectations;			
Ingram (2016)[227]	Neonatal	Planning	Process	Patient centered	Discharge education for patients and families; Planning for discharge; Evaluating patient readiness for discharge;	(F) Provider-patient communication; Discharge education; Expectations;	(None)	(None)	Discharge assessment
Jefferies (2014)[228]	Neonatal	Evaluation; Planning	Process	Effective; Safe; Patient centered	Patient and family needs and experiences during discharge; Discharge education for patients and families; Planning for discharge; Evaluating patient readiness for discharge;	(F) Provider-patient communication; Discharge education; Family engagement/ support; Written communication; (B) Patient demographic and clinical characteristics; Socioeconomic factors;	(F) Provider-provider communication; Written documentation;	(F) Follow-up clinic or program;	(None)
Kim (2017)[229]	Neonatal	Follow-up	Outcome	Effective; Safe	Adverse events, readmission, and mortality following discharge; Resource use during discharge;	(F) Patient demographic and clinical characteristics;	(None)	(F) Impact of current discharge practices;	(None)
Lakshmanan (2019)[230]	Neonatal	Execution	Structure	Patient centered	Patient and family needs and experiences during discharge; Discharge education for patients	(F) Provider-patient communication; Discharge education; Family	(None)	(F) Discharge location;	(None)

Author (Year)	Study Population	Phase(s) of Care	Donabedian[a]	IoM[b]	Themes	Patient & Family Facilitators (F) & Barriers (B)	Provider Facilitators (F) & Barriers (B)	Organizational Facilitators (F) & Barriers (B)	Tool
					and families; Anxiety associated with discharge; Autonomy;	engagement/ support; (B) Patient demographic and clinical characteristics; Feelings of anxiety or embarrassment; Financial obstacles;			
Lau (2019)[231]	Neonatal	Execution	Structure	Safe; Patient centered	Patient and family needs and experiences during discharge; Discharge education for patients and families; Anxiety associated with discharge;	(F) Provider-patient communication; Discharge education; (B) Feelings of anxiety or embarrassment; Financial obstacles; Logistical barriers;	(None)	(None)	(None)
Lee (2019)[232]	Neonatal	Follow-up	Outcome	Patient centered	Patient and family needs and experiences during discharge; Discharge education for patients and families; Adverse events, readmission, and mortality following discharge; Autonomy;	(F) Family engagement/ support; (B) Feelings of anxiety or embarrassment;	(None)	(B) Discharge location;	(None)
Litt (2018)[233]	Neonatal	Follow-up	Process	Effective; Patient centered	Standardizing the discharge process; Adverse events, readmission, and mortality following discharge; Anxiety associated with discharge; Resource use during discharge;	(F) Provider-patient communication;	(F) Critical care transition program;	(None)	(None)
Lovejoy-Bluem (2014)[234]	Neonatal	Planning	Process	Patient centered	Patient and family needs and	(F) Discharge education; Family	(F) Critical care transition	(B) Infrastructure;	(None)

Author (Year)	Study Population	Phase(s) of Care	Donabedian[a]	IoM[b]	Themes	Patient & Family Facilitators (F) & Barriers (B)	Provider Facilitators (F) & Barriers (B)	Organizational Facilitators (F) & Barriers (B)	Tool
					experiences during discharge; Discharge education for patients and families; Planning for discharge; Evaluating patient readiness for discharge;	engagement/ support;	program; Knowledge/ experience of provider;		
Lundberg (2016)[235]	Neonatal	Evaluation; Follow-up	Process; Outcome	Safe; Patient centered	Adverse events, readmission, and mortality following discharge; Evaluating patient readiness for discharge;	(B) Patient demographic and clinical characteristics; Feelings of anxiety or embarrassment;	(F) Knowledge/ experience of provider;	(None)	(None)
McGowan (2017)[236]	Neonatal	Evaluation; Follow-up	Process; Outcome	Patient centered ; Equal	Patient and family needs and experiences during discharge; Discharge education for patients and families; Anxiety associated with discharge; Critical care transition program (nurse liaison, outreach team); Evaluating patient readiness for discharge;	(F) Discharge education; (F&B) Patient demographic and clinical characteristics; (B) Feelings of anxiety or embarrassment; Expectations; Lack of familial support;	(F) Critical care transition program;	(F) Tools to facilitate discharge;	(None)
McGowan (2019)[237]	Neonatal	Planning	Process	Patient centered	Patient and family needs and experiences during discharge; Discharge education for patients and families; Planning for discharge; Anxiety associated with discharge; Evaluating patient readiness for discharge;	(F) Provider-patient communication; Family engagement/ support; (B) Patient demographic and clinical characteristics;	(None)	(None)	(None)

Author (Year)	Study Population	Phase(s) of Care	Donabedian[a]	IoM[b]	Themes	Patient & Family Facilitators (F) & Barriers (B)	Provider Facilitators (F) & Barriers (B)	Organizational Facilitators (F) & Barriers (B)	Tool
						Feelings of anxiety or embarrassment;			
Moyer (2014)[238]	Neonatal	Planning; Execution; Follow-up	Process; Structure; Outcome	Safe; Patient centered	Patient and family needs and experiences during discharge; Discharge education for patients and families; Planning for discharge; Adverse events, readmission, and mortality following discharge; Critical care transition program (nurse liaison, outreach team);	(F) Provider-patient communication; Discharge education; Written communication;	(None)	(None)	(None)
Murphy & Ehritz (2020)[239]	Neonatal	Execution	Process; Structure	Safe; Patient centered	Patient and family needs and experiences during discharge; Continuity of patient care; Discharge education for providers;	(F) Discharge education;	(F) Provider-provider communication; Collaboration between ICU and ward;	(None)	(None)
Murray (2016)[240]	Neonatal	Execution	Process	Safe; Patient centered	Discharge education for patients and families; Adverse events, readmission, and mortality following discharge; Continuity of patient care; Autonomy;	(F) Provider-patient communication; Discharge education;	(None)	(F) Education/ training of providers;	(None)
Noble (2018)[241]	Neonatal	Evaluation; Execution	Process	Effective	Patient and family needs and experiences during discharge; Planning for discharge; Evaluating patient readiness for discharge;	(F) Patient demographic and clinical characteristics; Family engagement/ support;	(None)	(F) Use of best practices; Tools to facilitate discharge;	Guideline/ checklist

Author (Year)	Study Population	Phase(s) of Care	Donabedian[a]	IoM[b]	Themes	Patient & Family Facilitators (F) & Barriers (B)	Provider Facilitators (F) & Barriers (B)	Organizational Facilitators (F) & Barriers (B)	Tool
Osorio (2017)[242]	Neonatal	Execution	Process	Patient centered	Patient and family needs and experiences during discharge; Discharge education for patients and families; Anxiety associated with discharge;	(F) Provider-patient communication; Discharge education; Family engagement/ support; Written communication; Excited, joyous to be leaving ICU; (F&B) Patient demographic and clinical characteristics; (B) Feelings of anxiety or embarrassment; Expectations; Logistical barriers;	(F) Provider-provider communication;	(None)	(None)
Peacock (2014)[243]	Neonatal	Execution	Process	Efficient; Patient centered	Planning for discharge; Standardizing the discharge process;	(None)	(F) Provider-provider communication; Written documentation;	(F) Guidelines or policies;	(None)
Peyrovi (2016)[244]	Neonatal	Planning	Process; Outcome	Effective; Patient centered	Patient and family needs and experiences during discharge; Discharge education for patients and families; Planning for discharge; Evaluating patient readiness for discharge; Resource use during discharge; Autonomy;	(F) Provider-patient communication; Discharge education; Family engagement/ support; Written communication;	(None)	(None)	(None)
Pineda (2020)[245]	Neonatal	Follow-up	Structure; Outcome	Effective; Efficient	Critical care transition program (nurse liaison, outreach	(None)	(F) Critical care transition program;	(F) Follow-up clinic or program; (B) Costs of	(None)

Author (Year)	Study Population	Phase(s) of Care	Donabedian[a]	IoM[b]	Themes	Patient & Family Facilitators (F) & Barriers (B)	Provider Facilitators (F) & Barriers (B)	Organizational Facilitators (F) & Barriers (B)	Tool
					team); Resource use during discharge;			healthcare provided;	
Purdy (2015)[246]	Neonatal	Planning; Follow-up	Process	Patient centered	Patient and family needs and experiences during discharge; Discharge education for patients and families; Planning for discharge; Resource use during discharge;	(F) Provider-patient communication; Discharge education; Family engagement/ support;	(None)	(None)	(None)
Quinn (2017)[247]	Neonatal	Planning	Process	Safe; Patient centered	Patient and family needs and experiences during discharge; Discharge education for patients and families; Standardizing the discharge process;	(F) Discharge education; Family engagement/ support; Expectations;	(None)	(F) Guidelines or policies;	(None)
Raffray (2014)[248]	Neonatal	Planning; Execution	Process; Structure	Patient centered ; Equal	Patient and family needs and experiences during discharge; Discharge education for patients and families; Planning for discharge; Continuity of patient care;	(F) Provider-patient communication; Discharge education; (B) Patient demographic and clinical characteristics; Socioeconomic factors;	(F) Knowledge/ experience of provider;	(F) Education/ training of providers; Follow-up clinic or program;	(None)
Raines (2013)[249]	Neonatal	Execution	Process	Patient centered	Patient and family needs and experiences during discharge; Anxiety associated with discharge;	(F) Provider-patient communication; Family engagement/ support; (B) Feelings of anxiety or embarrassment;	(None)	(None)	(None)

Author (Year)	Study Population	Phase(s) of Care	Donabedian[a]	IoM[b]	Themes	Patient & Family Facilitators (F) & Barriers (B)	Provider Facilitators (F) & Barriers (B)	Organizational Facilitators (F) & Barriers (B)	Tool
Raines (2013)[250]	Neonatal	Follow-up	Outcome	Patient centered	Patient and family needs and experiences during discharge; Discharge education for patients and families; Planning for discharge;	(F) Provider-patient communication; Discharge education; Family engagement/ support; Expectations; (B) Feelings of anxiety or embarrassment;	(None)	(None)	(None)
Raines (2017)[251]	Neonatal	Planning	Process	Safe; Patient centered	Patient and family needs and experiences during discharge; Discharge education for patients and families; Planning for discharge; Anxiety associated with discharge;	(F) Discharge education; Family engagement/ support; Expectations;	(None)	(F) Tools to facilitate discharge;	Educationa l tool
Reichert (2014)[252]	Neonatal	Planning	Process; Structure	Patient centered	Patient and family needs and experiences during discharge; Discharge education for patients and families; Planning for discharge;	(F) Provider-patient communication; Discharge education; Family engagement/ support; Expectations;	(F) Written documentation;	(F) Tools to facilitate discharge;	Guideline/ checklist
Sabzevari (2019)[253]	Neonatal	Planning	Structure	Safe; Patient centered	Patient and family needs and experiences during discharge; Discharge education for patients and families; Planning for discharge; Autonomy;	(F) Discharge education; Family engagement/ support; Expectations;	(None)	(None)	(None)
Schell (2016)[254]	Neonatal	Follow-up	Outcome	Safe	Adverse events, readmission, and mortality following discharge;	(B) Patient demographic and clinical characteristics;	(None)	(None)	(None)

Author (Year)	Study Population	Phase(s) of Care	Donabedian[a]	IoM[b]	Themes	Patient & Family Facilitators (F) & Barriers (B)	Provider Facilitators (F) & Barriers (B)	Organizational Facilitators (F) & Barriers (B)	Tool
						Socioeconomic factors;			
Serlachius (2018)[255]	Neonatal	Planning; Execution	Process	Patient centered	Patient and family needs and experiences during discharge; Availability of complete and accurate discharge information; Planning for discharge; Anxiety associated with discharge; Autonomy;	(F) Provider-patient communication; Written communication; (B) Expectations; Lack of provider-patient communication; Feelings of lack of control;	(None)	(None)	(None)
Setiawan (2019)[256]	Neonatal	Planning; Execution; Follow-up	Process; Outcome	Effective; Patient centered	Patient and family needs and experiences during discharge; Availability of complete and accurate discharge information; Discharge education for patients and families; Planning for discharge; Anxiety associated with discharge; Critical care transition program (nurse liaison, outreach team); Continuity of patient care;	(F) Provider-patient communication; Discharge education; Family engagement/ support; Written communication; (B) Patient demographic and clinical characteristics; Feelings of anxiety or embarrassment;	(None)	(None)	(None)
Seyedfarajollah (2018)[257]	Neonatal	Planning; Execution	Process; Structure	Effective; Efficient	Discharge education for patients and families; Planning for discharge; Standardizing the discharge process; Evaluating patient	(F) Discharge education;	(F) Written documentation;	(F) Tools to facilitate discharge;	(None)

**Author (Year)**	**Study Population**	**Phase(s) of Care**	**Donabedian[a]**	**IoM[b]**	**Themes**	**Patient & Family Facilitators (F) & Barriers (B)**	**Provider Facilitators (F) & Barriers (B)**	**Organizational Facilitators (F) & Barriers (B)**	**Tool**
					readiness for discharge;				
Smith (2020)[258]	Neonatal	Evaluation; Planning; Execution	Process; Structure	Effective	Patient and family needs and experiences during discharge; Availability of complete and accurate discharge information; Discharge education for patients and families; Planning for discharge; Standardizing the discharge process; Anxiety associated with discharge; Evaluating patient readiness for discharge; Discharge education for providers;	(F) Provider-patient communication; Discharge education; Family engagement/ support; Written communication; (B) Feelings of anxiety or embarrassment;	(F) Provider-provider communication;	(F) Education/ training of providers; Tools to facilitate discharge;	(None)
Snelling (2014)[259]	Neonatal	Evaluation; Execution; Follow-up	Process; Outcome	Safe; Patient centered	Patient and family needs and experiences during discharge; Critical care transition program (nurse liaison, outreach team); Continuity of patient care;	(F) Family engagement/ support; (B) Patient demographic and clinical characteristics; Feelings of anxiety or embarrassment;	(F) Critical care transition program;	(None)	(None)
Sommer (2015)[260]	Neonatal	Follow-up	Process	Patient centered	Patient and family needs and experiences during discharge; Anxiety associated with discharge;	(B) Feelings of anxiety or embarrassment; Lack of provider-patient communication;	(None)	(None)	(None)
Stanojevic (2018)[261]	Neonatal	Evaluation; Planning;	Process	Effective; Safe;	Patient and family needs and	(F) Discharge education; Family	(None)	(None)	(None)

Author (Year)	Study Population	Phase(s) of Care	Donabedian[a]	IoM[b]	Themes	Patient & Family Facilitators (F) & Barriers (B)	Provider Facilitators (F) & Barriers (B)	Organizational Facilitators (F) & Barriers (B)	Tool
		Execution; Follow-up		Patient centered	experiences during discharge; Discharge education for patients and families; Planning for discharge; Evaluating patient readiness for discharge;	engagement/ support; (F&B) Patient demographic and clinical characteristics; (B) Feelings of anxiety or embarrassment;			
Temple (2015)[262]	Neonatal	Evaluation	Process	Efficient; Timely	Planning for discharge; Evaluating patient readiness for discharge;	(F) Patient demographic and clinical characteristics;	(None)	(F) Impact of current discharge practices; Tools to facilitate discharge;	(None)
Toly (2019)[263]	Neonatal	Planning; Execution; Follow-up	Process; Outcome	Effective; Safe; Patient centered	Patient and family needs and experiences during discharge; Discharge education for patients and families; Planning for discharge; Adverse events, readmission, and mortality following discharge; Anxiety associated with discharge;	(F) Discharge education; Family engagement/ support; Coping mechanisms; (F&B) (Lack of) Provider-patient communication; (B) Feelings of anxiety or embarrassment; Expectations; Financial obstacles; Lack of familial support; Feelings of lack of control;	(F) Knowledge/ experience of provider;	(F) Education/ training of providers; Impact of current discharge practices;	(None)
Toral-Lopez (2016)[264]	Neonatal	Follow-up	Process; Structure	Patient centered	Patient and family needs and experiences during discharge; Anxiety associated with discharge; Critical care transition program (nurse liaison, outreach team);	(F) Provider-patient communication; Family engagement/ support;	(F) Critical care transition program; Clear roles and responsibilities;	(None)	(None)

Author (Year)	Study Population	Phase(s) of Care	Donabedian[a]	IoM[b]	Themes	Patient & Family Facilitators (F) & Barriers (B)	Provider Facilitators (F) & Barriers (B)	Organizational Facilitators (F) & Barriers (B)	Tool
					Continuity of patient care;				
Toral-Lopez (2017)[265]	Neonatal	Planning; Follow-up	Process; Structure	Safe; Patient centered	Discharge education for patients and families; Planning for discharge;	(F) Discharge education; Family engagement/ support;	(None)	(B) Costs of healthcare provided; Delay in discharge;	(None)
Turner (2013)[266]	Neonatal	Planning; Follow-up	Process; Outcome	Patient centered	Patient and family needs and experiences during discharge; Discharge education for patients and families; Anxiety associated with discharge; Autonomy;	(F) Discharge education; Family engagement/ support; Expectations;	(None)	(None)	(None)
Van Kampen (2019)[267]	Neonatal	Execution	Outcome	Safe	Discharge education for patients and families; Standardizing the discharge process;	(F) Discharge education; (B) Patient demographic and clinical characteristics;	(F) Critical care transition program;	(F) Discharge location; (B) Costs of healthcare provided;	(None)
Veronez (2017)[268]	Neonatal	Execution; Follow-up	Process	Patient centered	Patient and family needs and experiences during discharge; Anxiety associated with discharge;	(F) Family engagement/ support; Expectations; (B) Feelings of anxiety or embarrassment;	(None)	(None)	(None)
Vohr (2018)[269]	Neonatal	Follow-up	Outcome	Safe	Adverse events, readmission, and mortality following discharge; Critical care transition program (nurse liaison, outreach team); Continuity of patient care;	(F) Patient demographic and clinical characteristics; Discharge education; Family engagement/ support; Written communication; (B) Socioeconomic factors;	(F) Critical care transition program;	(None)	(None)

**Author (Year)**	**Study Population**	**Phase(s) of Care**	**Donabedian[a]**	**IoM[b]**	**Themes**	**Patient & Family Facilitators (F) & Barriers (B)**	**Provider Facilitators (F) & Barriers (B)**	**Organizational Facilitators (F) & Barriers (B)**	**Tool**
Voie (2018)[270]	Neonatal	Execution	Process	Safe; Patient centered	Continuity of patient care;	(None)	(F&B) (Lack of) Provider-provider communication;	(None)	(None)
Vonderheid (2016)[271]	Neonatal	Planning; Follow-up	Process; Structure; Outcome	Effective; Efficient; Patient centered	Patient and family needs and experiences during discharge; Anxiety associated with discharge; Critical care transition program (nurse liaison, outreach team); Continuity of patient care;	(F) Discharge education; (F&B) Patient demographic and clinical characteristics; (Lack of) Provider-patient communication;	(F) Critical care transition program;	(None)	(None)
Walter (2019)[272]	Neonatal	Planning	Process	Patient centered	Patient and family needs and experiences during discharge; Discharge education for providers;	(F) Family engagement/ support;	(F) Knowledge/ experience of provider;	(F) Education/ training of providers;	Educational tool
Waruingi (2015)[273]	Neonatal	Planning; Follow-up	Process; Outcome	Efficient; Safe	Adverse events, readmission, and mortality following discharge; Continuity of patient care;	(None)	(F) Critical care transition program;	(None)	(None)
Weiqing (2013)[274]	Neonatal	Planning; Execution	Process; Structure	Patient centered	Patient and family needs and experiences during discharge; Discharge education for patients and families;	(F) Provider-patient communication; Discharge education; Family engagement/ support;	(None)	(F) Discharge location;	(None)
Wellington (2016)[275]	Neonatal	Planning; Execution	Process	Effective; Safe	Planning for discharge; Standardizing the discharge process; Resource use during discharge;	(None)	(F) Clinical judgement or decision making;	(F) Guidelines or policies; Use of best practices;	(None)

Author (Year)	Study Population	Phase(s) of Care	Donabedian[a]	IoM[b]	Themes	Patient & Family Facilitators (F) & Barriers (B)	Provider Facilitators (F) & Barriers (B)	Organizational Facilitators (F) & Barriers (B)	Tool
White (2019)[276]	Neonatal	Follow-up	Outcome	Efficient; Safe	Patient and family needs and experiences during discharge; Adverse events, readmission, and mortality following discharge; Critical care transition program (nurse liaison, outreach team);	(F) Provider-patient communication; Discharge education; Family engagement/ support;	(F) Critical care transition program;	(F) Impact of current discharge practices;	(None)
Willard (2018)[277]	Neonatal	Follow-up	Process; Outcome	Effective; Efficient; Patient centered	Patient and family needs and experiences during discharge; Critical care transition program (nurse liaison, outreach team); Continuity of patient care;	(F) Provider-patient communication; Discharge education;	(F) Critical care transition program; Knowledge/ experience of provider;	(None)	(None)
Williams (2018)[278]	Neonatal	Planning; Execution; Follow-up	Structure; Outcome	Effective; Efficient; Safe; Timely	Planning for discharge;	(None)	(F) Critical care transition program; Clinical judgement or decision making;	(F) Use of best practices; Impact of current discharge practices; (B) Costs of healthcare provided;	(None)
Xiu-Xiang (2013)[279]	Neonatal	Planning; Execution	Process	Safe; Patient centered	Patient and family needs and experiences during discharge; Discharge education for patients and families; Anxiety associated with discharge; Continuity of patient care;	(F) Discharge education; (B) Feelings of anxiety or embarrassment;	(None)	(None)	(None)

**Author (Year)**	**Study Population**	**Phase(s) of Care**	**Donabedian[a]**	**IoM[b]**	**Themes**	**Patient & Family Facilitators (F) & Barriers (B)**	**Provider Facilitators (F) & Barriers (B)**	**Organizational Facilitators (F) & Barriers (B)**	**Tool**
Zahn (2020)[280]	Neonatal	Execution	Structure	Effective; Efficient; Safe	Standardizing the discharge process; Adverse events, readmission, and mortality following discharge; Medication reconciliation;	(None)	(F) Knowledge/ experience of provider;	(F) Education/ training of providers; (B) Impact of current discharge practices; Infrastructure;	(None)
Zamanzadeh (2013)[281]	Neonatal	Execution	Process	Effective; Patient centered	Patient and family needs and experiences during discharge; Discharge education for patients and families; St'	(F) Provider-patient communication; Discharge education; Family engagement/ support;	(F) Clinical judgement or decision making;	(F) Guidelines or policies; (B) Impact of current discharge practices; Delay in discharge;	(None)
Zamanzadeh (2013)[282]	Neonatal	Planning	Process	Patient centered	Patient and family needs and experiences during discharge; Discharge education for patients and families; Planning for discharge; Anxiety associated with discharge;	(F) Discharge education; Family engagement/ support; Expectations; (B) Feelings of anxiety or embarrassment;	(None)	(F) Impact of current discharge practices;	(None)
Zimmerman (2017)[283]	Neonatal	Planning; Execution; Follow-up	Process; Outcome	Safe; Patient centered	Patient and family needs and experiences during discharge; Discharge education for patients and families; Planning for discharge; Continuity of patient care; Autonomy;	(F) Provider-patient communication; Discharge education; Family engagement/ support; Written communication; Self-efficacy; Excited, joyous to be leaving ICU; (B) Patient demographic and clinical characteristics;	(None)	(F) Education/ training of providers; (B) Restricted visitation;	(None)

**Author (Year)**	**Study Population**	**Phase(s) of Care**	**Donabedian[a]**	**IoM[b]**	**Themes**	**Patient & Family Facilitators (F) & Barriers (B)**	**Provider Facilitators (F) & Barriers (B)**	**Organizational Facilitators (F) & Barriers (B)**	**Tool**
						Feelings of anxiety or embarrassment; Expectations;			
Attfiled (2018)[284]	Not reported	Execution	Structure	Efficient; Patient centered	Availability of complete and accurate discharge information; Standardizing the discharge process; Evaluating patient readiness for discharge; Medication reconciliation;	(None)	(F) Provider-provider communication; Clear roles and responsibilities;	(None)	Guideline/ checklist
Busico (2019)[285]	Not reported	Execution; Follow-up	Process	Effective; Efficient	Patient and family needs and experiences during discharge; Critical care transition program (nurse liaison, outreach team); Evaluating patient readiness for discharge; Continuity of patient care;	(F&B) Patient demographic and clinical characteristics; (B) Physical and psychological effects of illness;	(F) Critical care transition program;	(F) Follow-up clinic or program;	Guideline/ checklist
Cognet (2014)[286]	Not reported	Execution	Process; Structure	Patient centered	Standardizing the discharge process;	(B) Expectations;	(B) Lack of provider-provider communication;	(None)	(None)
Coombs (2015)[287]	Not reported	Planning; Execution; Follow-up	Process; Structure; Outcome	Effective; Patient centered	Patient and family needs and experiences during discharge; Planning for discharge; Autonomy;	(F) Family engagement/ support; Expectations;	(F) Provider-provider communication; Clinical judgement or decision making;	(F) Guidelines or policies; Use of best practices;	(None)
Cullinane (2013)[288]	Not reported	Execution	Process	Safe; Patient centered	Patient and family needs and experiences during discharge; Discharge education for patients	(F) Provider-patient communication; (B) Feelings of	(None)	(None)	(None)

Author (Year)	Study Population	Phase(s) of Care	Donabedian[a]	IoM[b]	Themes	Patient & Family Facilitators (F) & Barriers (B)	Provider Facilitators (F) & Barriers (B)	Organizational Facilitators (F) & Barriers (B)	Tool
					and families; Planning for discharge; Anxiety associated with discharge;	anxiety or embarrassment;			
de Grood (2019)[289]	Not reported	Execution	Process	Efficient; Patient centered	Availability of complete and accurate discharge information; Standardizing the discharge process;	(None)	(F) Clinical judgement or decision making; Clear roles and responsibilities;	(None)	(None)
Elliott (2013)[290]	Not reported	Follow-up	Outcome	Efficient; Safe	Adverse events, readmission, and mortality following discharge; Critical care transition program (nurse liaison, outreach team);	(None)	(F) Critical care transition program; (B) Workload; Lack of provider-provider communication; Lack of knowledge/ experience of provider;	(B) Limited ICU and ward resources; Impact of current discharge practices; Discharge location; Staffing;	(None)
Frølund (2013)[291]	Not reported	Execution	Process; Structure	Efficient; Safe; Patient centered	Patient and family needs and experiences during discharge; Planning for discharge; Continuity of patient care;	(F) Provider-patient communication;	(F) Provider-provider communication; Critical care transition program; (F&B) (Lack of) Knowledge/ experience of provider; (B) Provider anxiety;	(F) Education/ training of providers;	(None)
Guest (2017)[292]	Not reported	Execution	Process; Structure	Safe; Patient centered	Timeliness of discharge (time of day, delay); Critical care transition program (nurse liaison, outreach team);	(B) Feelings of anxiety or embarrassment; Physical and psychological effects of illness;	(F) Provider-provider communication; Critical care transition program;	(F) Education/ training of providers; (B) Limited ICU and ward resources; Time of discharge;	Guideline/ checklist

Author (Year)	Study Population	Phase(s) of Care	Donabedian[a]	IoM[b]	Themes	Patient & Family Facilitators (F) & Barriers (B)	Provider Facilitators (F) & Barriers (B)	Organizational Facilitators (F) & Barriers (B)	Tool
							Written documentation;		
Häggström (2013)[293]	Not reported	Planning; Execution	Process	Effective; Efficient; Safe	Planning for discharge; Standardizing the discharge process; Resource use during discharge; Continuity of patient care;	(B) Feelings of anxiety or embarrassment;	(F) Critical care transition program; Clinical judgement or decision making;	(F) Use of best practices; Tools to facilitate discharge; (B) Impact of current discharge practices; Reduction in technology and monitoring;	(None)
Häggström & Bäckström (2014)[294]	Not reported	Evaluation; Planning; Execution; Follow-up	Process	Effective; Safe; Patient centered ; Timely	Discharge education for patients and families; Planning for discharge; Anxiety associated with discharge; Timeliness of discharge (time of day, delay); Critical care transition program (nurse liaison, outreach team); Evaluating patient readiness for discharge; Continuity of patient care;	(F) Provider-patient communication; Discharge education; (B) Feelings of anxiety or embarrassment;	(F) Provider-provider communication; Critical care transition program; Collaboration between ICU and ward; Knowledge/ experience of provider; Clear roles and responsibilities;	(B) Delay in discharge;	(None)
Hall (2015)[295]	Not reported	Execution; Follow-up	Process; Outcome	Effective; Safe; Patient centered	Availability of complete and accurate discharge information; Planning for discharge; Standardizing the discharge process;	(None)	(F) Written documentation; (F&B) (Lack of) Provider-provider communication;	(F) Tools to facilitate discharge;	Guideline/ checklist
Johnson (2013)[296]	Not reported	Execution	Process	Efficient; Timely	Timeliness of discharge (time of day, delay);	(None)	(None)	(B) Time of discharge; Costs of healthcare	(None)

Author (Year)	Study Population	Phase(s) of Care	Donabedian[a]	IoM[b]	Themes	Patient & Family Facilitators (F) & Barriers (B)	Provider Facilitators (F) & Barriers (B)	Organizational Facilitators (F) & Barriers (B)	Tool
								provided; Discharge location;	
Kauppi (2018)[297]	Not reported	Execution	Process; Structure	Effective; Efficient	Resource use during discharge;	(None)	(B) Workload; Provider anxiety; Lack of knowledge/ experience of provider;	(B) Limited ICU and ward resources; Impact of current discharge practices; Hospital characteristics;	(None)
Kowitlawakul (2015)[298]	Not reported	Execution	Process; Structure	Patient centered	Availability of complete and accurate discharge information; Standardizing the discharge process;	(None)	(F) Provider-provider communication; Collaboration between ICU and ward; (B) Workload;	(F&B) Time of discharge;	(None)
Kraus (2016)[299]	Not reported	Execution	Structure	Effective	Availability of complete and accurate discharge information; Standardizing the discharge process; Continuity of patient care;	(None)	(F) Provider-provider communication; Collaboration between ICU and ward; Written documentation;	(None)	Discharge letter
Kumar (2015)[300]	Not reported	Execution; Follow-up	Process; Outcome	Efficient; Safe; Timely	Adverse events, readmission, and mortality following discharge; Timeliness of discharge (time of day, delay); Discharge education for providers;	(None)	(F) Collaboration between ICU and ward; (F&B) (Lack of) Provider-provider communication;	(F) Education/ training of providers;	(None)
Kwon (2014)[301]	Not reported	Planning; Follow-up	Process	Patient centered	Patient and family needs and experiences during discharge; Planning	(F) Provider-patient communication; Family	(None)	(None)	(None)

Author (Year)	Study Population	Phase(s) of Care	Donabedian[a]	IoM[b]	Themes	Patient & Family Facilitators (F) & Barriers (B)	Provider Facilitators (F) & Barriers (B)	Organizational Facilitators (F) & Barriers (B)	Tool
					for discharge; Anxiety associated with discharge;	engagement/ support; (F&B) Expectations; (B) Feelings of anxiety or embarrassment;			
Lin (2013)[302]	Not reported	Planning; Execution	Process; Structure	Efficient; Safe; Timely	Planning for discharge; Critical care transition program (nurse liaison, outreach team);	(None)	(F) Critical care transition program; Collaboration between ICU and ward; Written documentation; (B) Workload; Lack of provider-provider communication;	(F&B) Impact of current discharge practices; (B) Delay in discharge;	(None)
Lin (2013)[303]	Not reported	Evaluation; Planning; Execution	Process	Effective; Efficient; Timely	Discharge education for patients and families; Planning for discharge; Timeliness of discharge (time of day, delay); Resource use during discharge;	(None)	(F) Critical care transition program; Collaboration between ICU and ward; Clear roles and responsibilities; (B) Workload; Lack of provider-provider communication;	(B) Delay in discharge; Lack of education/ training of providers;	(None)
Ludin (2014)[304]	Not reported	Execution	Process	Effective	Discharge education for providers;	(None)	(F) Knowledge/ experience of provider;	(F) Education/ training of providers;	(None)
McNett (2019)[305]	Not reported	Execution	Process; Structure; Outcome	Effective; Safe; Timely	Planning for discharge; Adverse events, readmission, and mortality following discharge; Timeliness of discharge (time of day, delay);	(F) Patient demographic and clinical characteristics;	(F) Provider-provider communication; Collaboration between ICU and ward;	(F) Impact of current discharge practices;	(None)

**Author (Year)**	**Study Population**	**Phase(s) of Care**	**Donabedian[a]**	**IoM[b]**	**Themes**	**Patient & Family Facilitators (F) & Barriers (B)**	**Provider Facilitators (F) & Barriers (B)**	**Organizational Facilitators (F) & Barriers (B)**	**Tool**
Messing (2015)[306]	Not reported	Execution	Structure	Efficient	Availability of complete and accurate discharge information; Standardizing the discharge process;	(None)	(F) Provider-provider communication; Written documentation;	(F) Tools to facilitate discharge;	Transfer tool
Morgan (2018)[307]	Not reported	Execution	Process; Outcome	Safe; Timely	Adverse events, readmission, and mortality following discharge; Timeliness of discharge (time of day, delay);	(None)	(None)	(F&B) Time of discharge;	(None)
Nates (2016)[308]	Not reported	Planning; Execution	Process; Structure	Efficient; Safe; Timely	Standardizing the discharge process; Timeliness of discharge (time of day, delay);	(F) Patient demographic and clinical characteristics;	(None)	(F) Guidelines or policies; Use of best practices; Time of discharge; Discharge location; Admission location before ICU;	Guideline/ checklist
Reader (2018)[309]	Not reported	Evaluation	Process; Outcome	Safe; Equal; Timely	Evaluating patient readiness for discharge;	(None)	(F) Knowledge/ experience of provider;	(B) Limited ICU and ward resources; Costs of healthcare provided; Hospital characteristics;	(None)
Teixeira (2018)[310]	Not reported	Follow-up	Process; Structure; Outcome	Safe; Patient centered	Patient and family needs and experiences during discharge; Standardizing the discharge process; Critical care transition program (nurse liaison, outreach	(F) Family engagement/ support; (B) Physical and psychological effects of illness;	(F) Critical care transition program; Collaboration between ICU and ward; (B) Workload;	(F) Tools to facilitate discharge; (B) Costs of healthcare provided;	(None)

Author (Year)	Study Population	Phase(s) of Care	Donabedian[a]	IoM[b]	Themes	Patient & Family Facilitators (F) & Barriers (B)	Provider Facilitators (F) & Barriers (B)	Organizational Facilitators (F) & Barriers (B)	Tool
					team); Resource use during discharge; Medication reconciliation;				
Turnbull (2016)[311]	Not reported	Follow-up	Outcome	Patient centered	Availability of complete and accurate discharge information; Anxiety associated with discharge; Continuity of patient care;	(F) Provider-patient communication; Expectations;	(F) Critical care transition program; Knowledge/ experience of provider; Clinical judgement or decision making; (B) Lack of provider-provider communication;	(None)	(None)
Vijayaraghavan (2018)[312]	Not reported	Follow-up	Structure	Effective; Safe	Adverse events, readmission, and mortality following discharge; Continuity of patient care;	(B) Patient demographic and clinical characteristics;	(F) Provider-provider communication; Collaboration between ICU and ward; Multidisciplinary team;	(F) Use of best practices; Tools to facilitate discharge;	(None)
Wong (2013)[313]	Not reported	Execution	Process	Effective	Availability of complete and accurate discharge information; Continuity of patient care;	(B) Lack of provider-patient communication;	(F) Critical care transition program; Written documentation; (F&B) (Lack of) Provider-provider communication;	(F) Tools to facilitate discharge;	(None)
Yuan (2021)[314]	Not reported	Follow-up	Outcome	Effective; Safe	Adverse events, readmission, and mortality following discharge; Continuity of patient care;	(F) Family engagement/ support; (B) Patient demographic and clinical	(F) Knowledge/ experience of provider;	(F) Guidelines or policies; (B) Impact of current discharge practices;	(None)

Author (Year)	Study Population	Phase(s) of Care	Donabedian[a]	IoM[b]	Themes	Patient & Family Facilitators (F) & Barriers (B)	Provider Facilitators (F) & Barriers (B)	Organizational Facilitators (F) & Barriers (B)	Tool
						characteristics; Physical and psychological effects of illness;			

a. Donabedian framework elements include the process, structure, and outcome of discharge.

b. IoM = Institute of Medicine. This framework identifies the six domains of health care quality: Efficient, effective, safe, patient centered, timely, and equitable.

References:

1. Abitagaoglu S, Demiroluk O, Koksal C, Sezgin D, Turan G: Transition to home mechanical ventilation for geriatric patients with prolonged weaning. Turk Geriatri Dergisi. 2019, 22(1):18-24.
2. Academia EC, Gabriel CJ, Mueller A, Schwarz KA, Bartels K, Valuck RJ *et al*: Opioid prescribing after discharge in a previously mechanically ventilated, opioid-naive cohort. Ann Pharmacother. 2020, 54(11):1065-1072.
3. Agard AS, Hofhuis JGM, Koopmans M, Gerritsen RT, Spronk PE, Engelberg RA *et al*: Identifying improvement opportunities for patient- and family-centered care in the ICU: Using qualitative methods to understand family perspectives. J Crit Care. 2019, 49:33-37.
4. Alberto L, Zotarez H, Canete AA, Niklas JEB, Enriquez JM, Geronimo MR *et al*: A description of the ICU liaison nurse role in Argentina. Intensive Crit Care Nurs. 2014, 30(1):31-37.
5. Allum L, Connolly B, McKeown E: Meeting the needs of critical care patients after discharge home: a qualitative exploratory study of patient perspectives. Nurs Crit Care. 2018, 23(6):316-323.
6. Amass TH, Villa G, Omahony S, Badger JM, McFadden R, Walsh T *et al*: Family care rituals in the ICU to reduce symptoms of post-traumatic stress disorder in family members-A multicenter, multinational, before-and-after intervention trial. Crit Care Med. 2020, 48(2):176-184.
7. Anthes AM, Harinstein LM, Smithburger PL, Seybert AL, Kane-Gill SL: Improving adverse drug event detection in critically ill patients through screening intensive care unit transfer summaries. Pharmacoepidemiol Drug Saf. 2013, 22(5):510-516.
8. Azevedo LCP, de Souza IA, Zygun DA, Stelfox HT, Bagshaw SM: Association between nighttime discharge from the intensive care unit and hospital mortality: A multi-center retrospective cohort study. BMC Health Serv Res. 2015, 15:378.
9. Bagshaw SM, Tran DT, Opgenorth D, Wang X, Zuege DJ, Ingolfsson A *et al*: Assessment of costs of avoidable delays in intensive care unit discharge. JAMA Netw Open. 2020, 3(8):e2013913.
10. Baldwin MR, Wunsch H, Reyfman PA, Narain WR, Blinderman CD, Schluger NW *et al*: High burden of palliative needs among older intensive care unit survivors transferred to post-acute care facilities. a single-center study. Ann Am Thorac. 2013, 10(5):458-465.
11. Balshi AN, Huwait BM, Noor ASN, Alharthy AM, Madi AF, Ramadan OE *et al*: Modified Early Warning Score as a predictor of intensive care unit readmission within 48 hours: a retrospective observational study. Rev Bras Ter Intensiva. 2020, 32(2):301-307.
12. Basmaji J, Lau V, Lam J, Priestap F, Ball IM: Lessons learned and new directions regarding Discharge Direct from Adult Intensive Care Units Sent Home (DISH): A narrative review. J Intensive Care Soc. 2019, 20(2):165-170.
13. Bench S, Day T, Griffiths P: Effectiveness of critical care discharge information in supporting early recovery from critical illness. Crit Care Nurse. 2013, 33(3):41-52.
14. Bench SD, Heelas K, White C, Griffiths P: Providing critical care patients with a personalised discharge summary: a questionnaire survey and retrospective analysis exploring feasibility and effectiveness. Intensive Crit Care Nurs. 2014, 30(2):69-76.

15. Bench S, Day T, Heelas K, Hopkins P, White C, Griffiths P: Evaluating the feasibility and effectiveness of a critical care discharge information pack for patients and their families: a pilot cluster randomised controlled trial. BMJ Open. 2015, 5(11):e006852.
16. Bench S, Cornish J, Xyrichis A: Intensive care discharge summaries for general practice staff: a focus group study. Br J Gen Pract. 2016, 66(653):e904-e912.
17. Bloom SL, Stollings JL, Kirkpatrick O, Wang L, Byrne DW, Sevin CM *et al*: Randomized clinical trial of an ICU recovery pilot program for survivors of critical illness. Crit Care Med. 2019, 47(10):1337-1345.
18. Bose S, Johnson AEW, Moskowitz A, Celi LA, Raffa JD: Impact of intensive care unit discharge delays on patient outcomes: A retrospective cohort study. J Intensive Care Med. 2019, 34(11-12):924-929.
19. Bosma LBE, Hunfeld NGM, Quax RAM, Meuwese E, Melief PHGJ, van Bommel J *et al*: The effect of a medication reconciliation program in two intensive care units in the Netherlands: a prospective intervention study with a before and after design. Ann Intensive Care. 2018, 8(1):19.
20. Bosma LBE, van Rein N, Hunfeld NGM, Steyerberg EW, Melief PHGJ, van den Bemt PMLA: Development of a multivariable prediction model for identification of patients at risk for medication transfer errors at ICU discharge. PLoS One. 2019, 14(4):e0215459.
21. Boyd JM, Roberts DJ, Parsons Leigh J, Stelfox HT: Administrator perspectives on ICU-to-ward transfers and content contained in existing transfer tools: A cross-sectional survey. J Gen Intern Med. 2018, 33(10):1738-1745.
22. Brown SES, Ratcliffe SJ, Halpern SD: An empirical derivation of the optimal time interval for defining ICU readmissions. Med Care. 2013, 51(8):706-714.
23. Brown KN, Leigh JP, Kamran H, Bagshaw SM, Fowler RA, Dodek PM *et al*: Transfers from intensive care unit to hospital ward: a multicentre textual analysis of physician progress notes. Crit Care. 2018, 22(1):19.
24. Chaboyer W, Lin F, Foster M, Retallick L, Panuwatwanich K, Richards B: Redesigning the ICU nursing discharge process: A quality improvement study. Worldviews Evid Based Nurs. 2012, 9(1):40-48.
25. Chatterjee S, Sinha S, Todi SK: Transfer time from the intensive care unit and patient outcome: A retrospective analysis from a tertiary care hospital in India. Indian J Crit Care Med. 2019, 23(3):115-121.
26. Choi S, Lee J, Shin Y, Lee J, Jung J, Han M *et al*: Effects of a medical emergency team follow-up programme on patients discharged from the medical intensive care unit to the general ward: a single-centre experience. J Eval Clin Pract. 2016, 22(3):356-362.
27. Choi J, Lingler JH, Donahoe MP, Happ MB, Hoffman LA, Tate JA: Home discharge following critical illness: A qualitative analysis of family caregiver experience. Heart Lung. 2018, 47(4):401-407.
28. Choi J, Tate JA, Son Y-J: Challenges experienced by family caregivers of the adult intensive care unit patients in Korea: An integrative review. Clin Nurs Res. 2020:1054773820918433.
29. Churpek MM, Wendlandt B, Zadravecz FJ, Adhikari R, Winslow C, Edelson DP: Association between intensive care unit transfer delay and hospital mortality: A multicenter investigation. J Hosp Med. 2016, 11(11):757-762.
30. Coombs M, Long-Sutehall T, Darlington AS, Richardson A: Doctors' and nurses' views and experience of transferring patients from critical care home to die: A qualitative exploratory study. Palliative Medicine. 2015, 29(4):354-362.
31. Coombs MA, Darlington A-SE, Long-Sutehall T, Pattison N, Richardson A: Transferring patients home to die: what is the potential population in UK critical care units? BMJ Support Palliat Care. 2017, 7(1):98-101.
32. Coon EA, Kramer NM, Fabris RR, Burkholder DB, Klaas JP, Graff-Radford J *et al*: Structured handoff checklists improve clinical measures in patients discharged from the neurointensive care unit. Neurol Clin Pract. 2015, 5(1):42-49.
33. Correa TD, Ponzoni CR, Filho RR, Neto AS, Chaves RCdF, Pardini A *et al*: Nighttime intensive care unit discharge and outcomes: A propensity matched retrospective cohort study. PLoS One. 2018, 13(12):e0207268.

34. Coughlin DG, Kumar MA, Patel NN, Hoffman RL, Kasner SE: Preventing early bouncebacks to the neurointensive care unit: A retrospective analysis and quality improvement pilot. Neurocrit Care. 2018, 28(2):175-183.
35. Corner EJ, Soni N, Handy JM, Brett SJ: Construct validity of the Chelsea critical care physical assessment tool: an observational study of recovery from critical illness. Crit Care. 2014, 18(2):R55.
36. Cypress BS: Transfer out of intensive care: an evidence-based literature review. Dimens Crit Care Nurs. 2013, 32(5):244-261.
37. D'Angelo RG, Rincavage M, Tata AL, Millstein LS, Gulati MS, Flurie RW *et al*: Impact of an antipsychotic discontinuation bundle during transitions of care in critically ill patients. J Intensive Care Med. 2019, 34(1):40-47.
38. Darlington A-SE, Long-Sutehall T, Richardson A, Coombs MA: A national survey exploring views and experience of health professionals about transferring patients from critical care home to die. Palliat Med. 2015, 29(4):363-370.
39. de Grood C, Leigh JP, Bagshaw SM, Dodek PM, Fowler RA, Forster AJ *et al*: Patient, family and provider experiences with transfers from intensive care unit to hospital ward: a multicentre qualitative study. CMAJ. 2018, 190(22):E669-E676.
40. Detsky ME, Ailon J, Weinerman AS, Amaral AC, Bell CM: A two-site survey of clinicians to identify practices and preferences of intensive care unit transfers to general medical wards. J Crit Care. 2015, 30(2):358-362.
41. Ebrahimian A, Ghasemian-Nik H, Ghorbani R, Fakhr-Movahedi A: Development a reverse triage system based on modified sequential organ failure assessment for increasing the critical care surge capacity. Indian J Crit Care Med. 2018, 22(8):575-579.
42. Edenharter G, Gartner D, Heim M, Martin J, Pfeiffer U, Vogt F *et al*: Delay of transfer from the intensive care unit: a prospective observational analysis on economic effects of delayed in-house transfer. Eur J Med Res. 2019, 24(1):30.
43. El Hadidi S, Samir Bazan N, Byrne S, Darweesh E, Bermingham M: Heart failure prescribing quality at discharge from a critical care unit in Egypt: The impact of multidisciplinary care. Pharmacy. 2020, 8(3).
44. Enger R, Andershed B: Nurses' experience of the transfer of ICU patients to general wards: A great responsibility and a huge challenge. J Clin Nurs. 2018, 27(1-2):e186-e194.
45. Fabes J, Seligman W, Barrett C, McKechnie S, Griffiths J: Does the implementation of a novel intensive care discharge risk score and nurse-led inpatient review tool improve outcome? A prospective cohort study in two intensive care units in the UK. BMJ open. 2017, 7(12):e018322.
46. Fergusson NA, Ahkioon S, Ayas N, Dhingra VK, Chittock DR, Sekhon MS *et al*: Association between intensive care unit occupancy at discharge, afterhours discharges, and clinical outcomes: a historical cohort study. Can J Anaesth. 2020, 67(10):1359-1370.
47. Forster GM, Bihari S, Tiruvoipati R, Bailey M, Pilcher D: The association between discharge delay from intensive care and patient outcomes. Am J Respir Crit Care Med. 2020, 202(10):1399-1406.
48. Gantner D, Farley K, Bailey M, Huckson S, Hicks P, Pilcher D: Mortality related to after-hours discharge from intensive care in Australia and New Zealand, 2005-2012. Intensive Care Med. 2014, 40(10):1528-1535.
49. Garland A, Connors AF, Jr.: Optimal timing of transfer out of the intensive care unit. Am J Crit Care. 2013, 22(5):390-397.
50. Gilbert B, Morales JR, Searcy RJ, Johnson DW, Ferreira JA: Evaluation of neuroleptic utilization in the intensive care unit during transitions of care. J Intensive Care Med. 2017, 32(2):158-162.
51. Gimpel D, Shanbhag S, Srivastava T, MacLeod M, Conaglen P, Kejriwal N *et al*: Early discharge from intensive care after cardiac surgery is feasible with an adequate fast track, stepdown unit: Waikato experience. Heart Lung Circ. 2019, 28(12):1888-1895.
52. Goldstein A, Shahar Y, Orenbuch E, Cohen MJ: Evaluation of an automated knowledge-based textual summarization system for longitudinal clinical data, in the intensive care domain. Artif Intell Med. 2017, 82:20-33.
53. Gotur DB, Masud F, Paranilam J, Zimmerman JL: Analysis of rothman index data to predict postdischarge adverse events in a medical intensive care unit. J Intensive Care Med. 2018, 35(6):606-610.

54. Haggstrom M, Asplund K, Kristiansen L: Important quality aspects in the transfer process. Int J Health Care Qual Assur. 2014, 27(2):123-139.
55. Haggstrom M, Fjellner C, Ohman M, Rising Holmstrom M: Ward visits- one essential step in intensive care follow-up. An interview study with critical care nurses' and ward nurses'. Intensive Crit Care Nurs. 2018, 49:21-27.
56. Hajalizadeh A, Ahmadinejad M, Dehghan M, Arab M: Informational needs of the families of patients discharged from intensive care units: A case of Iran. Int Q Community Health Educ. 2020:272684X20972641.
57. Halvorson S, Wheeler B, Willis M, Watters J, Eastman J, O'Donnell R *et al*: A multidisciplinary initiative to standardize intensive care to acute care transitions. Int J Qual Health Care. 2016, 28(5):615-625.
58. Haraldsson L, Christensson L, Conlon L, Henricson M: The experiences of ICU patients during follow-up sessions - A qualitative study. Intensive Crit Care Nurs. 2015, 31(4):223-231.
59. Harlan EA, Valley TS: Navigating discharges from intensive care unit to ward. Ann Am Thorac. 2020, 17(7):807-808.
60. Herling SF, Brix H, Andersen L, Jensen LD, Handesten R, Knudsen H *et al*: Patient and spouses experiences with transition from intensive care unit to hospital ward - qualitative study. Scand J Caring Sci. 2020, 34(1):206-214.
61. Herve MEW, Zucatti PB, Lima MADDS: Transition of care at discharge from the Intensive Care Unit: a scoping review. Rev Lat Am Enfermagem. 2020, 28:e3325.
62. Heselmans A, van Krieken J, Cootjans S, Nagels K, Filliers D, Dillen K *et al*: Medication review by a clinical pharmacist at the transfer point from ICU to ward: a randomized controlled trial. J Clin Pharm Ther. 2015, 40(5):578-583.
63. Hoffman RL, Saucier J, Dasani S, Collins T, Holena DN, Fitzpatrick M *et al*: Development and implementation of a risk identification tool to facilitate critical care transitions for high-risk surgical patients. Int J Qual Health Care. 2017, 29(3):412-419.
64. Holland DE, Rhudy LM, Vanderboom CE, Bowles KH: Feasibility of discharge planning in intensive care units: A pilot study. Am J Crit Care. 2012, 21(4):e94-e101.
65. James S, Quirke S, McBride-Henry K: Staff perception of patient discharge from ICU to ward-based care. Nurs Crit Care. 2013, 18(6):297-306.
66. Jensen EK, Poulsen LM, Mathiesen O, Estrup S: Healthcare providers' knowledge and handling of impairments after intensive unit treatment: A questionnaire survey. Acta Anaesthesiol Scand. 2020, 64(4):532-537.
67. Jeong B-H, Na SJ, Lee D-S, Chung CR, Suh GY, Jeon K: Readmission and hospital mortality after ICU discharge of critically ill cancer patients. PLoS One. 2019, 14(1):e0211240.
68. Jolley SEMDM, Angus DCMDMPH, Clermont GMD, Hough CLMDM, Jolley SE, Angus DC *et al*: Discharge destination as a marker of mobility impairment in survivors of acute respiratory distress syndrome. Crit Care Med. 2019, 47(10):e814-e819.
69. Jonasdottir RJ, Klinke ME, Jonsdottir H: Integrative review of nurse-led follow-up after discharge from the ICU. J Clin Nurs. 2016, 25(1-2):20-37.
70. Jonasdottir RJ, Jones C, Sigurdsson GH, Jonsdottir H: Structured nurse-led follow-up for patients after discharge from the intensive care unit: Prospective quasi-experimental study. J Adv Nurs. 2018, 74(3):709-723.
71. Kastrup M, Powollik R, Balzer F, Röber S, Ahlborn R, von Dossow-Hanfstingl V *et al*: Predictive ability of the stability and workload index for transfer score to predict unplanned readmissions after ICU discharge*. Crit Care Med. 2013, 41(7):1608-1615.
72. Kheir F, Shawwa K, Nguyen D, Alraiyes AH, Simeone F, Nielsen ND: A 24-hour postintensive care unit transition-of-care model shortens hospital stay. J Intensive Care Med. 2016, 31(9):597-602.
73. King J, O'Neill B, Ramsay P, Linden MA, Darweish Medniuk A, Outtrim J *et al*: Identifying patients' support needs following critical illness: a scoping review of the qualitative literature. Crit Care. 2019, 23(1):187.
74. Klepstad PK, Nordseth T, Sikora N, Klepstad P: Use of national early warning score for observation for increased risk for clinical deterioration during post-ICU care at a surgical ward. Ther Clin Risk Manag. 2019, 15:315-322.

75. Kohn R, Harhay MO, Bayes B, Song H, Halpern SD, Kerlin MP *et al*: The influence of geographic dispersion on outcomes of hospitalized pulmonary service patients. Am J Respir Crit Care Med. 2019, 199(9).
76. Kram BL, Schultheis JM, Kram SJ, Cox CE: A pharmacy-based electronic handoff tool to reduce discharge prescribing of atypical antipsychotics initiated in the intensive care unit: A quality improvement initiative. J Pharm Pract. 2019, 32(4):434-441.
77. Kram BL, Kram SJ, Brooks KR: Implications of atypical antipsychotic prescribing in the intensive care unit. J Crit Care. 2015, 30(4):814-818.
78. Kuang-Ming L, Chung-Han H, Chih-Cheng L, Chien-Ming C, Chong-Chi C, Shyh-Ren C *et al*: The association between depression and length of stay in the intensive care unit. Medicine. 2020, 99(23):1-7.
79. Kucukosman G, Kocakulak D, Ayoglu H: Assessment of patients discharged from the intensive care unit with tracheostomy: A retrospective analysis of single-center data. Anestezi Dergisi. 2020, 28(1):52-58.
80. Lam JNH, Lau VI, Priestap FA, Basmaji J, Ball IM: Patient, family, and physician satisfaction with planning for direct discharge to home from intensive care units: Direct from ICU sent home study. J Intensive Care Med. 2017, 35(1):82-90.
81. Lau VI, Priestap FA, Lam JNH, Ball IM: Factors associated with the increasing rates of discharges directly home from intensive care units-A direct from ICU sent home study. J Intensive Care Med. 2018, 33(2):121-127.
82. Lau VI, Priestap F, Lam JNH, Basmaji J, Ball IM: Clinical predictors for unsafe direct discharge home patients from intensive care units. J Intensive Care Med. 2018, 35(10):1067-1073.
83. Lau VI, Lam JNH, Basmaji J, Priestap FA, Ball IM: Survival and safety outcomes of ICU patients discharged directly home-A direct from ICU sent homes study. Crit Care Med. 2018, 46(6):900-906.
84. Lee S, Oh H, Suh Y, Seo W: A tailored relocation stress intervention programme for family caregivers of patients transferred from a surgical intensive care unit to a general ward. J Clin Nurs. 2017, 26(5-6):784-794.
85. Lekwijit S, Chan CW, Green LV, Liu VX, Escobar GJ: The impact of step-down unit care on patient outcomes after ICU discharge. Crit Care Explor. 2020, 2(5):e0114.
86. Lin Y, Myall M, Jarrett N: Uncovering the decision-making work of transferring dying patients home from critical care units: An integrative review. J Adv Nurs. 2017, 73(12):2864-2876.
87. Lin Y-W, Zhou Y, Faghri F, Shaw MJ, Campbell RH: Analysis and prediction of unplanned intensive care unit readmission using recurrent neural networks with long short-term memory. PLoS One. 2019, 14(7):e0218942.
88. Major ME, van Nes F, Ramaekers S, Engelbert RHH, van der Schaaf M: Survivors of critical illness and their relatives. A qualitative study on hospital discharge experience. Ann Am Thorac. 2019, 16(11):1405-1413.
89. Martin CM, Lam M, Allen B, Richard L, Lau V, Ball IM *et al*: Determinants of direct discharge home from critical care units: A population-based cohort analysis. Crit Care Med. 2020, 48(4):475-483.
90. Martin ND, Pisa M, Collins T, Robertson M, Sicoutris C, Bushan N *et al*: Advanced practitioner-driven critical care outreach to reduce intensive care unit readmission mortality. Int J Acad Med. 2015, 1(1):3-8.
91. McCairn AJ, Jones C: Does time of transfer from critical care to the general wards affect anxiety? A pragmatic prospective cohort study. Intensive Crit Care Nurs. 2014, 30(4):219-225.
92. McWilliams CJ, Lawson DJ, Santos-Rodriguez R, Gilchrist ID, Champneys A, Gould TH *et al*: Towards a decision support tool for intensive care discharge: machine learning algorithm development using electronic healthcare data from MIMIC-III and Bristol, UK. BMJ Open. 2019, 9(3):e025925.
93. Milton A, Schandl A, Soliman I, Joelsson-Alm E, van den Boogaard M, Wallin E *et al*: ICU discharge screening for prediction of new-onset physical disability-A multinational cohort study. Acta Anaesthesiol Scand. 2020, 64(6):789-797.

94. Milton A, Schandl A, Soliman IW, Meijers K, van den Boogaard M, Larsson IM *et al*: Development of an ICU discharge instrument predicting psychological morbidity: a multinational study. Intensive Care Med. 2018, 44(12):2038-2047.
95. Mion M, Al-Janabi F, Islam S, Magee N, Balasubramanian R, Watson N *et al*: Care after REsuscitation: Implementation of the United Kingdom's first dedicated multidisciplinary follow-up program for survivors of out-of-hospital cardiac arrest. Ther Hypothermia Temp Manag. 2020, 10(1):53-59.
96. Moore AJ, Castle L, Russell R, Manuel A: Discharging patients from ICU. J Intensive Care Soc. 2013, 14(3):271-272.
97. Moreira HE, Verga F, Barbato M, Burghi G: Prognostic impact of the time of admission and discharge from the intensive care unit. Rev Bras Ter Intensiva. 2017, 29(1):63-69.
98. Morris NA, Batra A, Biffi A, Cohen AB: Transfer delays from the neurologic intensive care unit: A prospective cohort study. Neurohospitalist. 2016, 6(2):59-63.
99. Morton B, Penston V, McHale P, Hungerford D, Dempsey G: Clinician perception of long-term survival at the point of critical care discharge: a prospective cohort study. Anaesthesia. 2020, 75(7):896-903.
100. Murray NM, Joshi AN, Kronfeld K, Hobbs K, Bernier E, Hirsch KG *et al*: A standardized checklist improves the transfer of stroke patients from the neurocritical care unit to hospital ward. Neurohospitalist. 2020, 10(2):100-108.
101. Ng YH, Pilcher DV, Bailey M, Bain CA, MacManus C, Bucknall TK: Predicting medical emergency team calls, cardiac arrest calls and re-admission after intensive care discharge: creation of a tool to identify at-risk patients. Anaesth Intensive Care. 2018, 46(1):88-96.
102. Niven DJ, Bastos JF, Stelfox HT: Critical care transition programs and the risk of readmission or death after discharge from an ICU: a systematic review and meta-analysis. Crit Care Med. 2014, 42(1):179-187.
103. Ofoma UR, Chandra S, Kashyap R, Herasevich V, Ahmed A, Gajic O *et al*: Findings from the implementation of a validated readmission predictive tool in the discharge workflow of a medical intensive care unit. Ann Am Thorac. 2014, 11(5):737-743.
104. Ofoma UR, Dong Y, Gajic O, Pickering BW: A qualitative exploration of the discharge process and factors predisposing to readmissions to the intensive care unit. BMC Health Serv Res. 2018, 18(1):6.
105. Ofoma UR, Montoya J, Saha D, Berger A, Kirchner HL, McIlwaine JK *et al*: Associations between hospital occupancy, intensive care unit transfer delay and hospital mortality. J Crit Care. 2020, 58:48-55.
106. Oh H, Lee S, Kim J, Lee E, Min H, Cho O *et al*: Clinical validity of a relocation stress scale for the families of patients transferred from intensive care units. J Clin Nurs. 2015, 24(13-14):1805-1814.
107. Ostergaard KL, Simonsen J, Hertzum M: The handover from intensive care unit to general ward: **B**aseline performance and participatory design of an electronic follow-up plan. Stud Health Technol Inform. 2019, 264:1303-1307.
108. Oud L, Chan YM: Predictors and variation of routine home discharge in critically ill adults with cystic fibrosis. Heart Lung. 2018, 47(5):511-515.
109. Parenmark F, Karlstrom G, Nolin T, Fredrikson M, Walther SM: Reducing night-time discharge from intensive care. A nationwide improvement project with public display of ICU outcomes. J Crit Care. 2019, 49:7-13.
110. Patel PM, Fiorella MA, Zheng A, McDonnell L, Yasuoka M, Yoo EJ: Characteristics and outcomes of patients discharged directly home from a medical intensive care unit: A retrospective cohort study. J Intensive Care Med. 2020:885066620960637.
111. Pattison N, O'Gara G, Rattray J: After critical care: patient support after critical care. A mixed method longitudinal study using email interviews and questionnaires. Intensive Crit Care Nurs. 2015, 31(4):213-222.
112. Peltonen L-M, McCallum L, Siirala E, Haataja M, Lundgren-Laine H, Salantera S *et al*: An Integrative literature review of organisational factors associated with admission and discharge delays in critical care. Biomed Res Int. 2015, 2015:868653.
113. Peters JS: Role of transitional care measures in the prevention of readmission after critical illness. Crit Care Nurse. 2017, 37(1):e10-e17.

114. Powell M, Brown D, Davis C, Walsham J, Calleja P, Nielsen S *et al*: Handover practices of nurses transferring trauma patients from intensive care units to the ward: A multimethod observational study. Aust Crit Care. 2020, 33(6):538-545.
115. Rai S, Anthony L, Needham DM, Georgousopoulou EN, Sudheer B, Brown R *et al*: Barriers to rehabilitation after critical illness: a survey of multidisciplinary healthcare professionals caring for ICU survivors in an acute care hospital. Aust Crit Care. 2020, 33(3):264-271.
116. Ramos A, Benitez-Gutierrez L, Asensio A, Ruiz-Antoran B, Folguera C, Sanchez-Romero I *et al*: Erratum to antimicrobial stewardship in patients recently transferred to a ward from the ICU. Rev Esp Quimioter. 2014, 27(2):134-139.
117. Ramsay P, Huby G, Thompson A, Walsh T: Intensive care survivors' experiences of ward-based care: Meleis' theory of nursing transitions and role development among critical care outreach services. J Clin Nurs. 2014, 23(5-6):605-615.
118. Ramsay P, Huby G, Merriweather J, Salisbury L, Rattray J, Griffith D *et al*: Patient and carer experience of hospital-based rehabilitation from intensive care to hospital discharge: mixed methods process evaluation of the RECOVER randomised clinical trial. BMJ open. 2016, 6(8):e012041.
119. Ranzani OT, Zampieri FG, Taniguchi LU, Forte DN, Azevedo LCP, Park M: The effects of discharge to an intermediate care unit after a critical illness: a 5-year cohort study. J Crit Care. 2014, 29(2):230-235.
120. Rath K, Kreitzer N, Schlichter E, Lopez O, Ferioli S, Ngwenya LB *et al*: The experience of a neurocritical care admission and discharge for patients and their families: A qualitative analysis. J Neurosci Nurs. 2020, 52(4):179-185.
121. Reineck LA, Le TQ, Seymour CW, Barnato AE, Angus DC, Kahn JM: Effect of public reporting on intensive care unit discharge destination and outcomes. Ann Am Thorac. 2015, 12(1):57-63.
122. Rice M, Lear A, Kane-Gill S, Seybert AL, Smithburger PL: Pharmacy personnel's involvement in transitions of care of intensive care unit patients: A systematic review. J Pharm Pract. 2020:897190020911524.
123. Rizvi MS, Nei AM, Gajic O, Mara KC, Barreto EF: Continuation of newly initiated midodrine therapy after intensive care and hospital discharge: A single-center retrospective study. Crit Care Med. 2019, 47(8):e648-e653.
124. Rojas JC, Carey KA, Edelson DP, Venable LR, Howell MD, Churpek MM: Predicting intensive care unit readmission with machine learning using electronic health record data. Ann Am Thorac. 2018, 15(7):846-853.
125. Safavi KC, Furtado J, Zenteno Langle AC, Scheinker D, Schmidt U, Daily B *et al*: Non-clinical delays in transfer out of the surgical ICU are associated with increased hospital length of stay and delayed progress of care. J Crit Care. 2019, 50:126-131.
126. Sanson G, Marino C, Valenti A, Lucangelo U, Berlot G: Is my patient ready for a safe transfer to a lower-intensity care setting? Nursing complexity as an independent predictor of adverse events risk after ICU discharge. Heart Lung. 2020, 49(4):407-414.
127. Santamaria JD, Duke GJ, Pilcher DV, Cooper DJ, Moran J, Bellomo R *et al*: The timing of discharge from the intensive care unit and subsequent mortality. A prospective, multicenter study. Am J Respir Crit Care Med. 2015, 191(9):1033-1039.
128. Santhosh L, Lyons PG, Rojas JC, Ciesielski TM, Beach S, Farnan JM *et al*: Characterising ICU-ward handoffs at three academic medical centres: process and perceptions. BMJ Qual Saf. 2019, 28(8):627-634.
129. Sauro KM, Soo A, De Grood C, Yang MMH, Wierstra B, Benoit L *et al*: Adverse events after transition from ICU to hospital ward: A multicenter cohort study. Crit Care Med. 2020:946-953.
130. Sayde GE, Stefanescu A, Conrad E, Nielsen N, Hammer R: Implementing an intensive care unit (ICU) diary program at a large academic medical center: Results from a randomized control trial evaluating psychological morbidity associated with critical illness. Gen Hosp Psychiatry. 2020, 66:96-102.
131. Sevin CM, Bloom SL, Jackson JC, Wang L, Ely EW, Stollings JL: Comprehensive care of ICU survivors: Development and implementation of an ICU recovery center. J Crit Care. 2018, 46:141-148.
132. Sevin CM, Jackson JC: Post-ICU clinics should be staffed by ICU clinicians. Crit Care Med. 2019, 47(2):268-272.

133. Shimogai T, Izawa KP, Kawada M, Kuriyama A: Factors affecting discharge to home of medical patients treated in an intensive care unit. Int J Environ Res Public Health. 2019, 16(22).
134. Siddiqui S: Patients readmitted to the intensive care unit: can they be prevented? Int Arch Med. 2013, 6(1):18.
135. Sirvent JM, Gil M, Alvarez T, Martin S, Vila N, Colomer M *et al*: Lean techniques to improve the flow of critically ill patients in a health region with its epicenter in the intensive care unit of a reference hospital. Med Intensiva. 2016, 40(5):266-272.
136. Smith D, Hall D, Parke G, Lane R, Gray A: No place like home: A case study of a patient discharged from an Intensive Care Unit for end of life care at home. J Intensive Care Soc. 2018, 19(3):259-263.
137. Stelfox HT, Bastos J, Niven DJ, Bagshaw SM, Turin TC, Gao S: Critical care transition programs and the risk of readmission or death after discharge from ICU. Intensive Care Med. 2016, 42(3):401-410.
138. Stelfox HT, Parsons Leigh J, Dodek PM, Turgeon AF, Forster AJ, Lamontagne F *et al*: A multi-centre prospective cohort study of patient transfers from the intensive care unit to the hospital ward. Am J Respir Crit Care Med. 2017, 195.
139. Stelfox HT, Soo A, Niven DJ, Fiest KM, Wunsch H, Rowan KM *et al*: Assessment of the safety of discharging select patients directly home from the intensive care unit: A multicenter population-based cohort study. JAMA Intern Med. 2018, 178(10):1390-1399.
140. Szubski CR, Tellez A, Klika AK, Xu M, Kattan MW, Guzman JA *et al*: Predicting discharge to a long-term acute care hospital after admission to an intensive care unit. Am J Crit Care. 2014, 23(4):e46-53.
141. Taniguchi LU, Ramos FJdS, Momma AK, Martins Filho APR, Bartocci JJ, Lopes MFD *et al*: Subjective score and outcomes after discharge from the intensive care unit: a prospective observational study. J Int Med Res. 2019, 47(9):4183-4193.
142. Tully AP, Hammond DA, Li C, Jarrell AS, Kruer RM: Evaluation of medication errors at the transition of care from an ICU to non-ICU location. Crit Care Med. 2019, 47(4):543-549.
143. Uppanisakorn S, Bhurayanontachai R, Boonyarat J, Kaewpradit J: National Early Warning Score (NEWS) at ICU discharge can predict early clinical deterioration after ICU transfer. J Crit Care. 2018, 43:225-229.
144. van Mol M, Nijkamp M, Markham C, Ista E: Using an intervention mapping approach to develop a discharge protocol for intensive care patients. BMC Health Serv Res. 2017, 17(1):837.
145. van Mol M, Ista E, van Dijk M: Implementation and evaluation of a follow-up programme after intensive care treatment: A practice development project. Intensive Crit Care Nurs. 2018, 49:6-13.
146. van Sluisveld N, Bakhshi-Raiez F, de Keizer N, Holman R, Wester G, Wollersheim H *et al*: Variation in rates of ICU readmissions and post-ICU in-hospital mortality and their association with ICU discharge practices. BMC Health Serv Res. 2017, 17(1):281.
147. Vollam S, Dutton S, Lamb S, Petrinic T, Young JD, Watkinson P: Out-of-hours discharge from intensive care, in-hospital mortality and intensive care readmission rates: a systematic review and meta-analysis. Intensive Care Med. 2018, 44(7):1115-1129.
148. Vollam S, Harrison DA, Young JD, Watkinson PJ: Does delaying discharge from intensive care until after tracheostomy removal affect 30-day mortality? Propensity score matched cohort study. BMJ open. 2020, 10(6):e037762.
149. Wagner J, Gabler NB, Ratcliffe SJ, Brown SES, Strom BL, Halpern SD: Outcomes among patients discharged from busy intensive care units. Ann Intern Med. 2013, 159(7):447-455.
150. White L: Going home to die from critical care: A case study. Nurs Crit Care. 2019, 24(4):235-240.
151. Won MH, Son Y-J: Development and psychometric evaluation of the Relocation Stress Syndrome Scale-Short Form for patients transferred from adult intensive care units to general wards. Intensive Crit Care Nurs. 2020, 58:102800.
152. Wood SD, Coster S, Norman I: Comparing the monitoring of patients transferred from a critical care unit to hospital wards at after-hours with day transfers: an exploratory, prospective cohort study. J Adv Nurs. 2014, 70(12):2757-2766.

153. Xing S, Roshdy A, Radhakrishnan J, Kiff K, Collins J: Discharge home from critical care: safety assessment in a resource constrained system. J R Coll Physicians Edinb. 2019, 49(1):23-25.
154. Yang S, Wang Z, Liu Z, Wang J, Ma L: Association between time of discharge from ICU and hospital mortality: a systematic review and meta-analysis. Crit Care. 2016, 20(1):390.
155. Yun SH, Oh EG, Yoo YS, Kim SS, Jang YS: Development and effects of a transition nursing program for patients and family caregivers at a neurological ICU in Korea. Clin Nurs Res. 2017, 26(1):27-46.
156. Zaidi H, Bader-El-Den M, McNicholas J: Using the National Early Warning Score (NEWS/NEWS 2) in different Intensive Care Units (ICUs) to predict the discharge location of patients. BMC Public Health. 2019, 19(1):1231.
157. Zilahi G, O'Connor E: Information sharing between intensive care and primary care after an episode of critical illness; A mixed methods analysis. PLoS One. 2019, 14(2):e0212438.
158. Tiruvoipati R, Botha J, Fletcher J, Gangopadhyay H, Majumdar M, Vij S *et al*: Intensive care discharge delay is associated with increased hospital length of stay: A multicentre prospective observational study. PLoS ONE. 2017, 12(7):e0181827.
159. van Sluisveld N, Hesselink G, van der Hoeven JG, Westert G, Wollersheim H, Zegers M: Improving clinical handover between intensive care unit and general ward professionals at intensive care unit discharge. Intensive Care Med. 2015, 41(4):589-604.
160. van Sluisveld N, Oerlemans A, Westert G, van der Hoeven JG, Wollersheim H, Zegers M: Barriers and facilitators to improve safety and efficiency of the ICU discharge process: a mixed methods study. BMC Health Serv Res. 2017, 17(1):251.
161. Herbst LA, Desai S, Benscoter D, Jerardi K, Meier KA, Statile AM *et al*: Going back to the ward-transitioning care back to the ward team. Transl Pediatr. 2018, 7(4):314-325.
162. Li P, Boyd JM, Ghali WA, Stelfox HT: Stakeholder views regarding patient discharge from intensive care: Suboptimal quality and opportunities for improvement. Can Respir J. 2015, 22(2):109-118.
163. Alali H, Kazzaz Y, Alshehri A, Antar M, Alhamouieh O, Hasan Z *et al*: Reducing unnecessary delays during the transfer of patients from the paediatric intensive care unit to the general ward: a quality improvement project. BMJ Open Qual. 2019, 8(3):e000695.
164. Baker CD, Martin S, Thrasher J, Moore HM, Baker J, Abman SH *et al*: A standardized discharge process decreases length of stay for ventilator-dependent children. Pediatrics. 2016, 137(4).
165. Bedford ZC, Bench S: A review of interventions supporting parent's psychological well-being after a child's intensive care unit discharge. Nurs Crit Care. 2019, 24(3):153-161.
166. Berube KM, Fothergill-Bourbonnais F, Thomas M, Moreau D: Parents' experience of the transition with their child from a pediatric intensive care unit (PICU) to the hospital ward: searching for comfort across transitions. J Pediatr Nurs. 2014, 29(6):586-595.
167. Crowe L, Simpson E, Reinhardt Z, Rankin J: Parental responsibility for pediatric ventricular assist devices: Views of families on the acceptability of hospital discharge. Pediatr Transplant. 2020, 24(1):e13636.
168. de la Oliva P, Cambra-Lasaosa FJ, Quintana-Diaz M, Rey-Galan C, Sanchez-Diaz JI, Martin-Delgado MC *et al*: Admission, discharge and triage guidelines for paediatric intensive care units in Spain. An Pediatr (Barc). 2018, 42(4):235-246.
169. Ertugrul A, Bayrakci B, Ertugrul I, Kesici S, Gunes Yalcin E: Clinical evaluation of invasive home mechanical ventilation dependent pediatric patients. Iran J Pediatr. 2017, 27(4):e9531.
170. Foster K, Mitchell R, Young A, Van C, Curtis K: Parent experiences and psychosocial support needs 6 months following paediatric critical injury: A qualitative study. Injury. 2019, 50(5):1082-1088.
171. Frankel LR, Hsu BS, Yeh TS, Simone S, Agus MSD, Arca MJ *et al*: Criteria for critical care infants and children: PICU admission, discharge, and triage practice statement and levels of care guidance. Pediatr Crit Care Med. 2019, 20(9):847-887.

172. Garcia X, Frazier E, Kane J, Jones A, Brown C, Bryant T *et al*: Pediatric cardiac critical care transport and palliative care: A case series. Am J Hosp Palliat Care. 2021, 38(1):94-97.
173. Goldstein RF, Malcolm WF: Care of the neonatal intensive care unit graduate after discharge. Pediatr Clin North Am. 2019, 66(2):489-508.
174. Graham RJ, Amar-Dolan LR, Roussin CJ, Weinstock PH: Bridging the stressful gap between ICU and home: medical simulation for pediatric patients and their families. Pediatr Crit Care Med. 2019, 20(4):e221-e224.
175. Kennedy TK, Numa A: Factors associated with discharge delay and direct discharge home from paediatric intensive care. J Paediatr Child Health. 2020, 56(7):1101-1107.
176. Kroeger AR, Morrison J, Smith AH: Predicting unplanned readmissions to a pediatric cardiac intensive care unit using predischarge Pediatric Early Warning Scores. Congenit Heart Dis. 2018, 13(1):98-104.
177. Kulesa JT, Balsara SL, Ghebremariam ET, Colyer J: Designing a process for cardiology patient transfers: A quality improvement, descriptive study on interprovider communication and resident education. Pediatr Qual Saf. 2020, 5(3):e300.
178. Leyenaar JK, Desai AD, Burkhart Q, Parast L, Roth CP, McGalliard J *et al*: Quality measures to assess care transitions for hospitalized children. Pediatrics. 2016, 138(2).
179. Lobos A-T, Fernandes R, Willams K, Ramsay C, McNally JD: Routine medical emergency team assessments of patients discharged from the PICU: Description of a medical emergency team follow-up program. Pediatr Crit Care Med. 2015, 16(4):359-365.
180. Manente L, McCluskey T, Shaw R: Transitioning patients from the intensive care unit to the general pediatric unit: A piece of the puzzle in family-centered care. Pediatr Nurs. 2017, 43(2):77-82.
181. Moore M, Robinson G, Mink R, Hudson K, Dotolo D, Gooding T *et al*: Developing a family-centered care model for critical care after pediatric traumatic brain injury. Pediatr Crit Care Med. 2015, 16(8):758-765.
182. Neupane B, McFeeters M, Johnson E, Hickey H, Pandya H: Transitioning children requiring long-term ventilation from hospital to home: A practical guide. Paediatr Child Health. 2015, 25(4):187-191.
183. Noje C, Bernier ML, Costabile PM, Klein BL, Kudchadkar SR: Pediatric critical care transport as a conduit to terminal extubation at home: A case series. Pediatr Crit Care Med. 2017, 18(1):e4-e8.
184. Obas KA, Leal JM, Zegray M, Rennick JE: Parental perceptions of transition from intensive care following a child's cardiac surgery. Nurs Crit Care. 2016, 21(3):e1-9.
185. Orenstein EW, Ferro DF, Bonafide CP, Landrigan CP, Gillespie S, Muthu N: Hidden health IT hazards: a qualitative analysis of clinically meaningful documentation discrepancies at transfer out of the pediatric intensive care unit. JAMIA open. 2019, 2(3):392-398.
186. Sheth S, McCarthy E, Kipps AK, Wood M, Roth SJ, Sharek PJ *et al*: Changes in efficiency and safety culture after integration of an I-PASS-supported handoff process. Pediatrics. 2016, 137(2):e20150166.
187. Smith AH, Anand V, Banerjee M, Bates KE, Brunetti MA, Cooper DS *et al*: Variation in case-mix adjusted unplanned pediatric cardiac ICU readmission rates. Crit Care Med. 2018, 46(12):e1175-e1182.
188. Sobotka SA, Dholakia A, Agrawal RK, Berry JG, Brenner M, Graham RJ *et al*: Discharge practices for children with home mechanical ventilation across the United States. Key-informant perspectives. Ann Am Thorac. 2020, 17(11):1424-1430.
189. Vollmer A-M, Skonetzki-Cheng S, Prokosch H-U: Analysis of the interface and data transfer from ICU to normal wards in a German University Hospital. Stud Health Technol Inform. 2013, 192:1104.
190. Warrick D, Gonzalez-del-Rey J, Hall D, Statile A, White C, Simmons J *et al*: Improving resident handoffs for children transitioning from the intensive care unit. Hospital Pediatrics. 2015, 5(3):127-133.

191. Barone S, Boss RD, Raisanen JC, Shepard J, Donohue PK: Our life at home: Photos from families inform discharge planning for medically complex children. Birth. 2020.
192. Morton KE, Richardson A, Coombs MA, Darlington A-SE: Transferring critically ill babies and children home to die from intensive care. Nurs Crit Care. 2019, 24(4):222-228.
193. Amirani L, Namnabati M, Hajiheidari M: Examining the effect of discharge plan on enabling mothers of preterm babies admitted to the neonatal intensive care unit (NICU). Ann Trop Med Public Health. 2018, 13(Special Issue):SX746.
194. Aydon L, Hauck Y, Murdoch J, Siu D, Sharp M: Transition from hospital to home: Parents' perception of their preparation and readiness for discharge with their preterm infant. J Clin Nurs. 2018, 27(1-2):269-277.
195. Bapat R, McClead R, Shepherd E, Ryshen G, Bartman T: Challenges, successes and opportunities for reducing readmissions in a referral-based children's hospital NICU. J Neonatal Perinatal Med. 2016, 9(4):433-440.
196. Barkemeyer BM: Discharge planning. Pediatr Clin North Am. 2015, 62(2):545-556.
197. Bathie J, Shaw J: Early discharge home from the neonatal unit with the support of naso-gastric tube feeding. J Neonatal Nurs. 2013, 19(4):213-216.
198. Berman L, Raval MV, Ottosen M, Mackow AK, Cho M, Goldin AB: Parent perspectives on readiness for discharge home after neonatal intensive care unit admission. J Pediatr. 2019, 205:98-104.e104.
199. Bos LK, Shorey S, Kulantaipian TS, Sng JSP, Tam WWS, Koh SSL: Effectiveness of the neonatal discharge program for very low-birth-weight infants on parental efficacy and psychological distress. J Perinat Neonatal Nurs. 2018, 32(4):E11-E21.
200. Boss RD, Hobbs JE: Continuity of care for NICU graduates. Contemp Pediatr. 2013, 30(10):28-34.
201. Bowles JD, Jnah AJ, Newberry DM, Hubbard CA, Roberston T: Infants with technology dependence: facilitating the road to home. Adv Neonatal Care. 2016, 16(6):424-429.
202. Boykova M: Transition from hospital to home in preterm infants and their families. J Perinat Neonatal Nurs. 2016, 30(3):270-272.
203. Brodsgaard A, Zimmermann R, Petersen M: A preterm lifeline: Early discharge programme based on family-centred care. J Spec Pediatr Nurs. 2015, 20(4):232-243.
204. Buck CO, Tucker R, Vohr B, McGowan EC: Predictors of parenting readiness in fathers of high-risk infants in the neonatal intensive care unit. J Pediatr. 2020, 217:192-195.e191.
205. Burgoa-Larranaga SL, Iglesias-Leboreiro J, Bernardez-Zapata I: Early discharge of the healthy newborn from the nursery of the Hospital Español de México. Rev Med Inst Mex Seguro Soc. 2016, 54(6):696-705.
206. Burnham N, Feeley N, Sherrard K: Parents' perceptions regarding readiness for their infant's discharge from the NICU. Neonatal Netw. 2013, 32(5):324-334.
207. Carlos C, Hageman J, Pellerite M, McEntire B, Cote A, Raoux A *et al*: Neonatal intensive care unit discharge of infants with cardiorespiratory events: Tri-country comparison of academic centers. J Neonatal Perinatal Med. 2015, 8(4):307-311.
208. Carty CL, Soghier LM, Kritikos KI, Tuchman LK, Jiggetts M, Glass P *et al*: The giving parents support study: A randomized clinical trial of a parent navigator intervention to improve outcomes after neonatal intensive care unit discharge. Contemp Clin Trials. 2018, 70:117-134.
209. Chen Y, Zhang J, Bai J: Effect of an educational intervention on parental readiness for premature infant discharge from the neonatal intensive care units. J Adv Nurs. 2016, 72(1):135-146.
210. Coughlin K, Posencheg M, Orfe L, Zachritz W, Meadow J, Yang K *et al*: Reducing Variation in the Management of Apnea of Prematurity in the Intensive Care Nursery. Pediatrics. 2020, 145(2).
211. Cresi F, Cocchi E, Maggiora E, Pirra A, Logrippo F, Ariotti MC *et al*: Pre-discharge cardiorespiratory monitoring in preterm infants. the CORE study. Front Pediatr. 2020, 8:234.

212. Daicampi C, Comoretto RI, Soriani N, Lolli E, Boschetto S, Cecchin M *et al*: Protocols for early discharging of premature infants: an empirical assessment on safety and savings. J Perinat Med. 2019, 47(8):885-893.
213. Driscoll C, El Metwally D: A daily huddle facilitates patient transports from a neonatal intensive care unit. BMJ Qual Improv Rep. 2014, 3(1).
214. Enlow E, Herbert SL, Jovel IJ, Lorch SA, Anderson C, Chamberlain LJ: Neonatal intensive care unit to home: the transition from parent and pediatrician perspectives, a prospective cohort study. J Perinatol. 2014, 34(10):761-766.
215. Enlow E, Faherty LJ, Wallace-Keeshen S, Martin AE, Shea JA, Lorch SA: Perspectives of low socioeconomic status mothers of premature infants. Pediatrics. 2017, 139(3).
216. Enlow E, Gray MM, Wallace-Keeshen S, D'Agostino JA, Abbasi S, Lorch SA: Health literacy of parents of very preterm infants at NICU admission and discharge: a prospective cohort study. J Perinatol. 2019, 39(6):866-875.
217. Ermarth A, Thomas D, Ling CY, Cardullo A, White BR: Effective tube weaning and predictive clinical characteristics of NICU patients with feeding dysfunction. JPEN J Parenter Enteral Nutr. 2020, 44(5):920-927.
218. Feehan K, Kehinde F, Sachs K, Mossabeb R, Berhane Z, Pachter LM *et al*: Development of a multidisciplinary medical home program for NICU graduates. Matern Child Health J. 2020, 24(1):11-21.
219. Fleming PJ, Ingram J, Johnson D, Blair PS: Estimating discharge dates using routinely collected data: improving the preparedness of parents of preterm infants for discharge home. Arch Dis Child Fetal Neonatal Ed. 2017, 102(2):F170-F172.
220. Gad A, Parkinson E, Khawar N, Elmeki A, Narula P, Hoang D: Perspectives and attitudes of pediatricians concerning post-discharge care practice of premature infants. J Neonatal Perinatal Med. 2017, 10(1):99-107.
221. Garfield CF, Lee Y, Kim HN: Paternal and maternal concerns for their very low-birth-weight infants transitioning from the NICU to home. J Perinat Neonatal Nurs. 2014, 28(4):305-312.
222. Giudici L, Rodriguez D, Alonso MM, Bertani G, Cattaino A, Fernandez P *et al*: Guidelines for discharge planning of the high risk newborn. Arch Argent Pediatr. 2018, 116(4):S77-S81.
223. Goldin AB, Raval MV, Thurm CW, Hall M, Billimoria Z, Juul S *et al*: The resource use inflection point for safe NICU discharge. Pediatrics. 2020, 146(2).
224. Granero-Molina J, Fernandez Medina IM, Fernandez-Sola C, Hernandez-Padilla JM, Jimenez Lasserrotte MDM, Lopez Rodriguez MDM: Experiences of mothers of extremely preterm infants after hospital discharge. J Pediatr Nurs. 2019, 45:e2-e8.
225. Gupta M, Pursley DM, Smith VC: Preparing for discharge from the neonatal intensive care unit. Pediatrics. 2019, 143(6).
226. Hobbs JE, Tschudy MM, Hussey-Gardner B, Jennings JM, Boss RD: "I don't know what I was expecting": Home visits by neonatology fellows for infants discharged from the NICU. Birth. 2017, 44(4):331-336.
227. Ingram JC, Powell JE, Blair PS, Pontin D, Redshaw M, Manns S *et al*: Does family-centred neonatal discharge planning reduce healthcare usage? A before and after study in South West England. BMJ Open. 2016, 6(3):e010752.
228. Jefferies AL, Canadian Paediatric Society F, Newborn C: Going home: Facilitating discharge of the preterm infant. Paediatr Child Health. 2014, 19(1):31-42.
229. Kim JS, Shim JW, Lee JH, Chang YS, Committee on Data C, Statistical Analysis tKSoN: Comparison of follow-up courses after discharge from neonatal intensive care unit between very low birth weight infants with and without home oxygen. J Korean Med Sci. 2017, 32(8):1295-1303.
230. Lakshmanan A, Kubicek K, Williams R, Robles M, Vanderbilt DL, Mirzaian CB *et al*: Viewpoints from families for improving transition from NICU-to-home for infants with medical complexity at a safety net hospital: a qualitative study. BMC Pediatr. 2019, 19(1):223.
231. Lau R, Crump RT, Brousseau DC, Panepinto JA, Nicholson M, Engel J *et al*: Parent preferences regarding home oxygen sse for infants with bronchopulmonary dysplasia. J Pediatr. 2019, 213:30-37.e33.

232. Lee SY, Chau JPC, Choi KC, Lo SHS: Feasibility of a guided participation discharge program for very preterm infants in a neonatal intensive care unit: a randomized controlled trial. BMC Pediatr. 2019, 19(1):402.
233. Litt JS, Agni M, Jacobi-Polishook T, Melvin P, McCormick MC, Stewart JE *et al*: The acceptability and feasibility of emailed parent questionnaires for medical and developmental surveillance after NICU discharge. J Perinatol. 2018, 38(4):392-401.
234. Lovejoy-Bluem A: Neonatal intensive care unit discharge transitioning: Nursing practices, perspectives, and perceptions. Brandman University; 2014.
235. Lundberg B, Lindgren C, Palme-Kilander C, Örtenstrand A, Bonamy AKE, Sarman I: Hospital-assisted home care after early discharge from a Swedish neonatal intensive care unit was safe and readmissions were rare. Acta Paediatrica. 2016, 105(8):895-901.
236. McGowan EC, Du N, Hawes K, Tucker R, O'Donnell M, Vohr B: Maternal mental health and neonatal intensive care unit readiness in mothers of preterm infants. J Pediatr. 2017, 184:68-74.
237. McGowan EC, Abdulla LS, Hawes KK, Tucker R, Vohr BR: Maternal immigrant status and readiness to transition to home from the NICU. Pediatrics. 2019, 143(5).
238. Moyer VA, Papile L-A, Eichenwald E, Giardino AP, Khan MM, Singh H: An intervention to improve transitions from NICU to ambulatory care: quasi-experimental study. BMJ Qual Saf. 2014, 23(12):1-7.
239. Murphy S, Ehritz C: Clinical nurse specialist practice strategies for children with medical complexity. Clin Nurse Spec. 2021, 35(1):38-43.
240. Murray CH, Joseph RA: Transition from NICU to home: are the parents ready to manage any emergency? An evidence-based project. Neonatal Netw. 2016, 35(3):151-155.
241. Noble LM, Okogbule-Wonodi AC, Young MA: ABM Clinical Protocol #12: Transitioning the breastfeeding preterm infant from the neonatal intensive care unit to home. Breastfeed Med. 2018, 13(4):230-236.
242. Osorio Galeano SP, Ochoa Marin SC, Semenic S: Preparing for post-discharge care of premature infants: Experiences of parents. Investigacion y educacion en enfermeria. 2017, 35(1):100-106.
243. Peacock JJ: Discharge summary for medically complex infants transitioning to primary care. Neonatal Netw. 2014, 33(4):204-207.
244. Peyrovi H, Mosayebi Z, Mohammad-Doost F, Chehrzad M-M, Mehran A: The effect of empowerment program on "perceived readiness for discharge" of mothers of premature infants. J Matern Fetal Neonatal Med. 2016, 29(5):752-757.
245. Pineda R, Heiny E, Nellis P, Smith J, McGrath JM, Collins M *et al*: The Baby Bridge program: A sustainable program that can improve therapy service delivery for preterm infants following NICU discharge. PLoS One. 2020, 15(5):e0233411.
246. Purdy IB, Craig JW, Zeanah P: NICU discharge planning and beyond: recommendations for parent psychosocial support. J Perinatol. 2015, 35 Suppl 1:S24-28.
247. Quinn JM, Sparks M, Gephart SM: Discharge criteria for the late preterm infant: A review of the literature. Adv Neonatal Care. 2017, 17(5):362-371.
248. Raffray M, Semenic S, Galeano SO, Ochoa Marin SC: Barriers and facilitators to preparing families with premature infants for discharge home from the neonatal unit: Perceptions of health care providers. Investigacion & Educacion en Enfermeria. 2014, 32(3):379-392.
249. Raines DA: Mothers' stressor as the day of discharge from the NICU approaches. Adv Neonatal Care. 2013, 13(3):181-187.
250. Raines DA: Preparing for NICU discharge: mothers' concerns. Neonatal Netw. 2013, 32(6):399-403.
251. Raines DA: Simulation as part of discharge teaching for parents of infants in the neonatal intensive care unit. MCN Am J Matern Child Nurs. 2017, 42(2):95-100.
252. Reichert J, Eulerich-Gyamerah S, Poets C, Kribs A, Roth B, Kuhn T *et al*: Psychological sociomedical care in neonatology. Monatsschr Kinderheilkd. 2014, 162(11):1010-1017.
253. Sabzevari S, Pouraboli B, Zade ZA, Nematollahi M, Dehesh T: Assessing the implementation of discharge planning on mothers' hope and satisfaction of preterm newborns in NICU. i-Managers Journal on Nursing. 2019, 8(4):1-9.

254. Schell S, Kase JS, Parvez B, Shah SI, Meng H, Grzybowski M *et al*: Maturational, comorbid, maternal and discharge domain impact on preterm rehospitalizations: a comparison of planned and unplanned rehospitalizations. J Perinatol. 2016, 36(4):317-324.
255. Serlachius A, Hames J, Juth V, Garton D, Rowley S, Petrie KJ: Parental experiences of family-centred care from admission to discharge in the neonatal intensive care unit. J Paediatr Child Health. 2018, 54(11):1227-1233.
256. Setiawan J, Mannix T, Sweet L: Understanding the effects of neonatal early discharge on parents: A literature review. J Perinat Neonatal Nurs. 2019, 33(2):170-188.
257. Seyedfarajollah S, Nayeri F, Kalhori SRN, Ghazisaeedi M, Keikha L: The Framework of NICU-discharge plan system for preterm infants in Iran: Duties, components and capabilities. Acta Inform Med. 2018, 26(1):46-50.
258. Smith VC: Discharge planning considerations for the neonatal intensive care unit. Arch Dis Child Fetal Neonatal Ed. 2020.
259. Snelling CH: Homeward Bound. OT Practice. 2014, 19(6):14-17.
260. Sommer CM, Cook CM: Disrupted bonds - parental perceptions of regionalised transfer of very preterm infants: a small-scale study. Contemp Nurse. 2015, 50(2-3):256-266.
261. Stanojevic M: School for parents - Preparation of preterm infants for discharge from the hospital. Paediatr Croat Suppl. 2018, 62(Supplement 1):80-85.
262. Temple MW, Lehmann CU, Fabbri D: Predicting discharge dates from the NICU using progress note data. Pediatrics. 2015, 136(2):e395-405.
263. Toly VB, Blanchette JE, Alhamed A, Musil CM: Mothers' voices related to caregiving: The transition of a technologydependent infant from the NICU to home. Neonatal Netw. 2019, 38(2):69-79.
264. Toral-Lopez I, Fernandez-Alcantara M, Gonzalez-Carrion P, Cruz-Quintana F, Rivas-Campos A, Perez-Marfil N: Needs perceived by parents of preterm infants: Integrating care Into the early discharge process. J Pediatr Nurs. 2016, 31(2):e99-e108.
265. Toral-Lopez I, Gonzalez-Carrion MP, Rivas-Campos A, Lafuente-Lorca J, Castillo-Vera J, de Casas C *et al*: Evolution of care indicators after an early discharge intervention in preterm infants. Enferm Clin. 2017, 27(4):235-240.
266. Turner M, Winefield H, Chur-Hansen A: The emotional experiences and supports for parents with babies in a neonatal nursery. Adv Neonatal Care. 2013, 13(6):438-446.
267. van Kampen F, de Mol A, Korstanje J, Groof FM-d, van Meurs-Asseler L, Stas H *et al*: Early discharge of premature infants < 37 weeks gestational age with nasogastric tube feeding: the new standard of care? Eur J Pediatr. 2019, 178(4):497-503.
268. Veronez M, Borghesan NAB, Correa DAM, Higarashi IH: Experience of mothers of premature babies from birth to discharge: notes of field journals. Rev Gaucha Enferm. 2017, 38(2):e60911.
269. Vohr B, McGowan E, Keszler L, O'Donnell M, Hawes K, Tucker R: Effects of a transition home program on preterm infant emergency room visits within 90 days of discharge. J Perinatol. 2018, 38(2):185-190.
270. Voie MP, Tunby J, Stromsvik N: Collaboration challenges faced by nurses when premature infants are discharged. Nursing Children and Young People. 2018, 30(2):33-38.
271. Vonderheid Sc RKNKVRHSW-TR: Health care use outcomes of an integrated hospital-to-home mother-preterm infant intervention. JOGNN - J Obstet Gynecol Neonatal Nurs. 2016, 45(5):625.
272. Walter L, Robb M: Promoting discharge readiness through staff education: A family-centered approach. J Nurses Prof Dev. 2019, 35(3):132-136.
273. Waruingi W, Iyer S, Collin M: Improving health care usage in a very low birth weight population. World J Pediatr. 2015, 11(3):239-244.
274. Weiqing S, Jianglan Y, Hong XU: Application of family - centered care model in discharge guidance of premature infants. Chinese Nursing Research. 2013, 27(4A):867-869.
275. Wellington G, Campbell AJ, Elder DE: Oximetry for preterm infants at neonatal discharge: What is current practice in New Zealand and Australia? J Paediatr Child Health. 2016, 52(3):333-337.

276. White BR, Ermarth A, Thomas D, Arguinchona O, Presson AP, Ling CY: Creation of a standard model for tube feeding at neonatal intensive care unit discharge. JPEN J Parenter Enteral Nutr. 2020, 44(3):491-499.
277. Willard A, Brown E, Masten M, Brant M, Pouppirt N, Moran K *et al*: Complexsurgical infants benefit from postdischarge telemedicine visits. Adv Neonatal Care. 2018, 18(1):22-30.
278. Williams G, Bada H, Chesnut L, Ferrell E, Mays GP: Examining the trade-off between NICU length of stay and postdischarge monitoring: An instrumental variables approach. J Healthc Manag. 2018, 63(5):301-311.
279. Xiu-Xiang H, Shun-Ying W: A nursing experience of assisting discharge planning for a mother of a premature infant. Tzu Chi Nursing Journal. 2013, 12(3):110-119.
280. Zahn SO, Tobison JH, Place AJ, Casey JN, Hess L, Foster T: Impact of a process change on prevalence of prescribed unmeasurable liquid doses in a neonatal intensive care unit. J Pediatr Pharmacol Ther. 2020, 25(2):96-103.
281. Zamanzadeh V, Namnabati M, Valizadeh L, Badiee Z: Professional's efforts to simultaneously discharge infants and mother from neonatal intensive care unit in Iran: A qualitative study. J Caring Sci. 2013, 2(1):39-45.
282. Zamanzadeh V, Namnabati M, Valizadeh L, Badiee Z: Mothers' experiences of infants discharge in Iranian NICU culture: a qualitative study. Adv Neonatal Care. 2013, 13(4):E1-7.
283. Zimmerman K: Discharge readiness for families with a premature infant living in Appalachia. East Tennessee State University; 2017.
284. Attfield E, Swankhuizen MP, Bruchet N, Slavik R, Gorman SK: Improving the clinical pharmacist handover process in the intensive care unit with a pharmacotherapy-specific tool: The I-HAPPY study. Can J Hosp Pharm. 2018, 71(2):111-118.
285. Busico M, das Neves A, Carini F, Pedace M, Villalba D, Foster C *et al*: Follow-up program after intensive care unit discharge. Med Intensiva. 2019, 43(4):243-254.
286. Cognet S, Coyer F: Discharge practices for the intensive care patient: a qualitative exploration in the general ward setting. Intensive Crit Care Nurs. 2014, 30(5):292-292.
287. Coombs MA, Darlington A-S, Long-Sutehall T, Richardson A: Transferring critically ill patients home to die: developing a clinical guidance document. Nurs Crit Care. 2015, 20(5):264-270.
288. Cullinane JP, Plowright CI: Patients' and relatives' experiences of transfer from intensive care unit to wards. Nurs Crit Care. 2013, 18(6):289-296.
289. de Grood C, Job McIntosh C, Boyd JM, Zjadewicz K, Parsons Leigh J, Stelfox HT: Identifying essential elements to include in Intensive Care Unit to hospital ward transfer summaries: A consensus methodology. J Crit Care. 2019, 49:27-32.
290. Elliott M, Worrall-Carter L, Page K: Factors contributing to adverse events after ICU discharge: a survey of liaison nurses. Aust Crit Care. 2013, 26(2):76-80.
291. Frølund C, Beedholm K: Erfaring og kompleksitet -- en kvalitativ undersøgelse af betydningsfulde forhold hos sygeplejerskerne ved overflytning af patienter fra intensiv til sengeafdeling. Experience and complexity - A qualitative study of conditions of significance for nurses during patient transition from the intensive care unit to the general ward. Vard Nord Utveckl Forsk. 2013, 33(4):9-13.
292. Guest M: Patient transfer from the intensive care unit to a general ward. Nurs Stand. 2017, 32(10):45-51.
293. Häggström M, Asplund K, Kristiansen L: To reduce technology prior discharge from intensive care - important but difficult? A grounded theory. Scand J Caring Sci. 2013, 27(3):506-515.
294. Haggstrom M, Backstrom B: Organizing safe transitions from intensive care. Nurs Res Pract. 2014, 2014:175314.
295. Hall W, Keane P, Wang S, Debell F, Allana A, Karia P: Intensive care discharges: improving the quality of clinical handover through changes to discharge documentation. BMJ Qual Improv Rep. 2015, 4(1).

296. Johnson DW, Schmidt UH, Bittner EA, Christensen B, Levi R, Pino RM: Delay of transfer from the intensive care unit: a prospective observational study of incidence, causes, and financial impact. Crit Care. 2013, 17(4):R128.
297. Kauppi W, Proos M, Olausson S: Ward nurses' experiences of the discharge process between intensive care unit and general ward. Nurs Crit Care. 2018, 23(3):127-133.
298. Kowitlawakul Y, Leong BSH, Lua A, Aroos R, Wong JJ, Koh N *et al*: Observation of handover process in an intensive care unit (ICU): barriers and quality improvement strategy. Int J Qual Health Care. 2015, 27(2):99-104.
299. Kraus S, Castellanos I, Albermann M, Schuettler C, Prokosch H-U, Staudigel M *et al*: Using Arden Syntax for the generation of intelligent intensive care discharge letters. Stud Health Technol Inform. 2016, 228:471-475.
300. Kumar P: Improving timely medical reviews for patients discharged from intensive care. BMJ Qual Improv Rep. 2015, 4(1).
301. Kwon H-K, Song M: Role adaptation processes of family caregivers with patients transferred from intensive care unit to general ward: Becoming almost a nurse with hope and fear. Korean Journal of Adult Nursing. 2014, 26(6):603-613.
302. Lin F, Chaboyer W, Wallis M: Understanding the distributed cognitive processes of intensive care patient discharge. J Clin Nurs. 2014, 23(5-6):673-682.
303. Lin F, Chaboyer W, Wallis M, Miller A: Factors contributing to the process of intensive care patient discharge: an ethnographic study informed by activity theory. Int J Nurs Stud. 2013, 50(8):1054-1066.
304. Ludin SM, Parker S, Arbon P: A survey of Malaysian critical intensive care unit nurses' awareness of patients' transition experiences (PE) and transitional care practice (TCP). Intensive Crit Care Nurs. 2014, 30(4):196-203.
305. McNett M, McLaughlin D: Transitions of care for patients with neurologic diagnoses transition from the intensive care unit to the floor. Nurs Clin North Am. 2019, 54(3):347-355.
306. Messing J: Improving handover from intensive care to ward medical teams with simple changes to paperwork. BMJ Qual Improv Rep. 2015, 4(1).
307. Morgan DJR, Ho KM: Impact of intensive care unit admission and discharge times on adverse outcomes after planned surgical cases. Response to Br J Anaesth 2018; 120: 1153-4. Br J Anaesth. 2018, 121(4):981-982.
308. Nates JL, Nunnally M, Kleinpell R, Blosser S, Goldner J, Birriel B *et al*: ICU admission, discharge, and triage guidelines: A framework to enhance clinical operations, development of institutional policies, and further research. Crit Care Med. 2016, 44(8):1553-1602.
309. Reader TW, Reddy G, Brett SJ: Impossible decision? An investigation of risk trade-offs in the intensive care unit. Ergonomics. 2018, 61(1):122-133.
310. Teixeira C, Rosa RG: Post-intensive care outpatient clinic: is it feasible and effective? A literature review. Rev Bras Ter Intensiva. 2018, 30(1):98-111.
311. Turnbull AE, Davis WE, Needham DM, White DB, Eakin MN: Intensivist-reported facilitators and barriers to discussing post-discharge outcomes with intensive care unit surrogates. A qualitative study. Ann Am Thorac. 2016, 13(9):1546-1552.
312. Vijayaraghavan BKT, Willaert X, Cuthbertson BH: Should ICU clinicians follow patients after ICU discharge? No. Intensive Care Med. 2018, 44(9):1542-1544.
313. Wong DJ, Wickham AJ: A survey of intensive care unit discharge communication practices in the UK. J Intensive Care Soc. 2013, 14(4):330-333.
314. Yuan C, Timmins F, Thompson DR: Post-intensive care syndrome: A concept analysis. Int J Nurs Stud. 2020, 114:103814.
