# Supplementary material for: Patient discharge from intensive care: an updated scoping review to identify tools and practices to inform high-quality care
Source: Crit Care. 2021 Dec 17;25:438. doi: 10.1186/s13054-021-03857-2 (PMC8684123; doi:10.1186/s13054-021-03857-2)
Supplement: Supplementary file 5 — Additional file 5. Discharge themes of articles included in the review [file 13054_2021_3857_MOESM5_ESM.pdf]

Additional File 5 - Discharge themes of articles included in the review.

**Theme**	**All studies, n(%) (N=314)**
Adverse events, readmission, and mortality following discharge	116 (36.9)
Patient and family needs and experiences during discharge	112 (35.7)
Planning for discharge	95 (30.3)
Continuity of patient care	84 (26.8)
Discharge education for patients and families	72 (22.9)
Standardizing the discharge process	60 (19.1)
Availability of complete and accurate discharge information	58 (18.5)
Evaluating patient readiness for discharge	58 (18.5)
Anxiety associated with discharge	56 (17.8)
Timeliness of discharge (time of day of discharge, delay in discharge)	53 (16.9)
Resource use during discharge	41 (13.1)
Critical care transition program (nurse liaison, outreach team)	39 (12.4)
Medication reconciliation	24 (7.6)
Autonomy	20 (6.4)
Discharge education for providers	17 (5.4)

Note: Responses are not mutually exclusive, and therefore, add up to more than 100%
